# Determination of potential thresholds for N-ethyl-N-nitrosourea and ethyl methanesulfonate based on a multi-endpoint genotoxicity assessment platform in rats

**DOI:** 10.1007/s11356-022-21605-z

**Published:** 2022-07-06

**Authors:** Xuejiao Zhu, Jiao Huo, Zhu Zeng, Yunjie Liu, Ruirui Li, Yiyi Chen, Lishi Zhang, Jinyao Chen

**Affiliations:** 1 Department of Nutrition and Food Safety, Chongqing Center for Disease Control and Prevention, Chongqing, China; 2 Department of Nutrition, Food Safety and Toxicology, West China School of Public Health, Sichuan University, Chengdu, Sichuan China; 3 West China School of Basic Medical Sciences and Forensic Medicine, Sichuan University, Chengdu, Sichuan China; 4 Chengdu Fifth People’s Hospital, Chengdu, Sichuan China; 5 Food Safety Monitoring and Risk Assessment Key Laboratory of Sichuan Province, Chengdu, Sichuan China

**Keywords:** Points of departure, *Pig-a* gene mutation assay, Micronucleus assay, Comet assay, Critical effect sizes, Benchmark dose modeling

## Abstract

The main goal of the study was to investigate the genotoxic response of N-ethyl-N-nitrosourea (ENU) and ethyl methanesulfonate (EMS) at low doses in a multi-endpoint genotoxicity assessment platform in rats and to derive potential thresholds and related metrics. Male Sprague–Dawley rats were treated by daily oral gavage for 28 consecutive days with ENU (0.25 ~ 8 mg/kg bw) and EMS (5 ~ 160 mg/kg bw), both with six closely spaced dose levels. *Pig-a* gene mutation assay, micronucleus test, and comet assay were performed in several timepoints. Then, the dose–response relationships were analyzed for possible points of departure (PoD) using the no observed genotoxic effect level and benchmark dose (BMD) protocols with different critical effect sizes (CES, 0.05, 0.1, 0.5, and 1SD). Overall, dose-dependent increases in all investigated endpoints were found for ENU and EMS. PoDs varied across genetic endpoints, timepoints, and statistical methods, and selecting an appropriate lower 95% confidence limit of BMD needs a comprehensive consideration of the mode of action of chemicals, the characteristics of tests, and the model fitting methods. Under the experimental conditions, the PoDs of ENU and EMS were 0.0036 mg/kg bw and 1.7 mg/kg bw, respectively.

**Supplementary Information:**

The online version contains supplementary material available at 10.1007/s11356-022-21605-z.

## Introduction

In genotoxic risk assessment, dichotomous qualitative evaluations have been traditionally employed, i.e., classification of chemicals as either a positive or negative for their genotoxicity. This qualitative classification method is based on the “single-hit” theory that even a single molecule, interacting with DNA, could result in DNA alterations and subsequent tumor initiation. However, in recent years, the tendency of genotoxic assessment has moved from “qualitative” to “quantitative.” There is an increasing appreciation of using quantitative methods to describe genotoxic dose–response relationships and then to derive potential thresholds that can be used as point of departure (PoD) (Johnson et al. 2014; Spassova 2018), which could further be used for human health risk assessment and regulatory decision-making.

PoD can be determined by different approaches such as the no observed genotoxic effect level (NOGEL), the breakpoint dose (BPD), and the benchmark dose (BMD) method based on different principles (Macgregor et al. 2015a, b). Compared with NOGEL and BPD, the BMD method fully considers all data and dose groups and is least affected by dose design spacing, and only three dose groups are needed for fitting. Therefore, BMD is recommended by the International Workshop on Genotoxicity Testing Workgroup (IWGT) on quantitative risk assessment of genetic toxicology as the primary choice used to define the PoD values. The BMD method was defined as the dose produces a predetermined change in response rate of an adverse effect compared with the background response induced by the tested substance (Hardy et al. 2017; Zeller et al. 2017). The change is called benchmark response (BMR) for BMDS or critical effect size (CES) for PROAST, depending on the software used. The choice of CES is crucial for the results obtained through PROAST. Several suggestions for setting CES of non-genotoxic endpoints have been published (Macgregor et al. 2015a, b). However, setting CES of genotoxic endpoints is more complex, and there is no consensus yet. In this study, several ways of deriving CES, such as CES 0.1, 0.5, and 1 (corresponding to 10, 50, and 100% increase over control) as well as CES 1SD (i.e., an increase of one standard deviation of the concurrent controls), were used to explore which CES values can better reflect the dose–response relationship of genotoxicity in each assay.

Alkylating agents are classical genotoxic carcinogens that induce damage mainly by acting directly with DNA. N-ethyl-N-nitrosourea (ENU) and ethyl methanesulfonate (EMS) are both monofunctional alkylating agent commonly used as positive agents in genotoxicity studies. Attributing to different mutagenic potencies, different adduct spectrums, and repair capacities, there may also be genotoxic thresholds for alkylating agents. In 2007, Viracept (nelfinavir mesilate), an HIV protease inhibitor provided by Roche, was contaminated at the time of manufacture, and the patients were exposed to EMS at a maximum dose of 0.055 mg/kg bw for 3 months. Due to lack of EMS data from human studies, a series of pilot studies were conducted to quantitatively assess the human carcinogenicity risk. It was established that EMS does not pose a health risk to humans at the accidental exposed doses (European Medicines Agency 2008; Gocke and Wall 2009; Müller et al. 2009). This contamination event became a typical example of the first practical application of quantitative genotoxicity risk assessment after actual human exposure. Research have discussed the thresholds of ENU and EMS (Gocke and Müller 2009; Guérard et al. 2017; Kraus et al. 2019), but the data are still insufficient. To minimize the genotoxic risk posed by unavoidable exposure, several working groups and consortia acknowledged the need for the integration of quantitative data and recommendations on different approaches (Gollapudi et al. 2013). The PoDs of ENU and EMS obtained from quantitative genotoxicity assessment can infer the acceptable risk to a certain hazard, and benefit for risk managers to give priority to evaluation and management of substances with high genotoxicity risk.

We used the *Pig-a* mutation assay and micronucleus test that we previously established (Huo et al. 2017, 2019; Liu et al. 2019; Zeng et al. 2020; Chen et al. 2021). On this basis, an in vivo multi-endpoint genotoxicity assessment platform was adopted by integrating the comet test and the above assays into 28-day repeat dose toxicology studies*.* To provide more insight into the quantitative genotoxicity threshold studies, the dose–response relationship of ENU and EMS was analyzed at different genotoxic endpoints. PoDs were determined by two ways including the NOGEL and BMD by the software PROAST with different critical effect size (CES 0.1, 0.5, 1, and 1SD). In this study, the assessment platform has the advantage that more information could be gathered in a shorter time period with significantly less animals, which allows it to be applied extensively in the practical work of genotoxic risk assessment of chemicals.

## Materials and methods

### Chemicals and reagents

ENU (CAS no. 759–73-9) and EMS (CAS no. 62–50-0), heparin sodium, low melting point agarose, Triton X-100, dimethyl sulfoxide were purchased from Sigma-Aldrich (Shanghai, China). Anti-CD59-APC and anti-CD71-FITC were obtained from BD Biosciences (San Jose, CA, USA). Anti-CD61-PE and anti-CD45-PE were purchased from eBiosciences (San Diego, CA, USA). DRAQ5 was provided by Abcam (Cambridge, UK). SYTO13 and propidium iodide (PI) were supplied by Invitrogen (Thermo Fisher Scientific, Inc., Waltham, MA, USA). Phosphate-buffered saline (PBS) and Hanks balanced salt mixture (HBSS) and fetal bovine serum (FBS) were purchased from Gibco, Thermo Fisher Scientific (Waltham, MA, USA). Normal melting point agarose and ethylenediaminetetraacetic acid (EDTA) and Tris base were purchased from Amresco (Shanghai, China).

### Animals and treatment

All experimental procedures were conducted on animals in compliance with guidelines of the Ethical Committee for Research on Laboratory Animals of Sichuan University. Five-week-old SPF-grade male SD rats were purchased from the Laboratory Animal Reproduction Center in Chengdu (Certificate No. SCXK 2015–030) and raised in No. 4 West China Teaching Hospital of Sichuan University (Certificate number: SYXK2018-011). Rats were housed in cages with a 12-h day and night cycle at room temperature between 20 ℃ and 23 ℃ and relative humidity of 60–65%, and received water and food ad libitum. Before dose administration, rats were acclimated for at least 5 days. Each treatment group consisted of six randomly selected rats, and rats were treated with ENU at 0 (PBS, PH = 6.0), 0.25, 0.5, 1, 2, 4, and 8 mg/kg/day, or with EMS at 0 (H_2_O, PH = 7.0), 5, 10, 20. 40, 80, and 160 mg/kg/day over 29 consecutive days. The dose levels were chosen according to references (Gollapudi et al. 2013) and preliminary experimental results (Ma et al. 2017). All treatments were via oral gavage at 10 mL/kg body weight, based on weekly recorded body weight.

### Blood and tissue collection

**Fig. 1:**
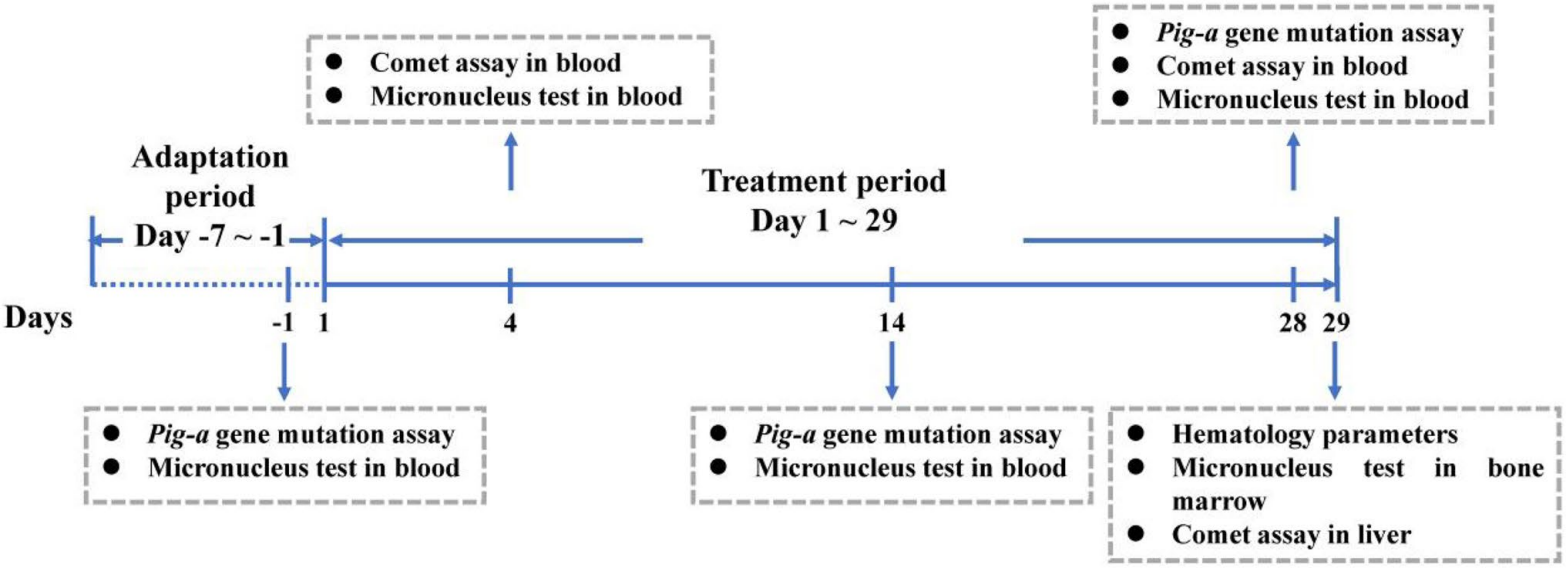
Total planning of experiment

The overview of the experimental protocol was presented in Fig. 1. Blood samples for various endpoints were collected according to the protocol-defined schedules. Blood samples were obtained from a tail vein using a 1-mL syringe fitted with a 25-gauge needle. Approximately 50 μL of free-flowing blood was collected directly drawn into a tube containing 10 µL heparin sodium (concentration 1000 U/mL). Micronucleus and comet assay blood and tissue were collected ~ 3 h after dosing. Blood samples for the comet assay were kept on ice and processed immediately, while samples for *Pig-a* assay and micronucleus assay were stored at 4 ℃ in the dark and processed within 2 h. After blood collection, animals were disinfected and hemostatic.

On day 29, all animals were killed 3 h after the last administration by exsanguination via the arteria femoralis under isofluorane anesthesia, and liver samples were excised at necropsy. Approximately 1000 μL of blood was collected for hematology evaluation. A middle section of the right lateral liver lobe (about 2 cm^3^) was minced in chilled mincing buffer (20 mmol/L EDTA in HBSS [Ca2 + and Mg2 + free] with 10% DMSO) prior to placement in fresh buffer to generate a single-cell suspension and held over ice until unwinding (within 2 h). Bone marrow for the micronucleus assay was prepared from both femurs of each animal and kept at 4 ℃ and processed within 2 h.

### Hematology evaluation

Hematology parameters were analyzed using a Medonic CA620 Hematology Analyzer (Stockholm, Sweden). The following parameters for hematology were analyzed: white blood cell (WBC) and granulocyte percent rate (GPR), lymphocyte percent rate (LPR), red blood cell (RBC) count, hemoglobin concentration (HGB), hematocrit (HCT), mean cell volume (MCV), mean cell hemoglobin (MCH), mean cell hemoglobin concentration (MCHC), red blood cell distribution width-coefficient of variation (RDW-CV), red blood cell distribution width-standard deviation (RDW-SD), platelet count (PLT), plateletocrit (PCT), mean platelet volume (MPV), platelet distribution width (PDW).

### Pig-a gene mutation assay

The methods have been reported in detail elsewhere (Huo et al. 2017, 2019). Refer to the methods recommended by the IWGT (Gollapudi et al. 2015). Cells were stained and modified as previously reported (Bryce et al. 2008; Phonethepswath et al. 2008). *Pig-a* analyses were performed on blood samples collected on days − 1 (before study), 14, and 28. About 20 µL blood from each tube was transferred to a new tube (containing 15 µL CD59-APC, 5 µL CD61-PE, 78 µL PBS, and 2 µL FBS) and protected from light throughout the experimental period. After incubating for 30 min, each tube was transferred to new tube containing 10 mL PBS, followed by centrifugation at 300 g/min for 5 min. The resulting supernatants were removed, and cells were resuspended, then labeled with 1 mL nucleic acid dye solution (150 nmol/L SYTO13). After incubating for 30 min at 37 ℃, samples were placed on ice until flow cytometric analysis (within 2 h).

Mutant mimic cells stained with SYTO 13 but not with antibody solution were prepared for instrument calibration before each batch of analysis. It is used to adjust compensation and to define the gating line for distinguishing mutant and wild-type cells.

Flow cytometric analysis was performed through a BD FACSuite Software Bundle v1.0. on a FACS Verse flow cytometer (BD Biosciences, NJ, USA) which provides 488 and 633-nm excitation. To obtain signal parameters, forward scatter (FSC), side scatter (SSC), FITC, APC, and PE channels were used. For each animal, the frequency of RBC^CD59−^ within the 1 × 10^6^ erythrocyte and the frequency of RET^CD59−^ within the 0.3 × 10^6^ reticulocyte, as well as the proportion of RETs in total RBCs, were calculated.

### Peripheral blood micronucleus assay

The test method was in accordance with OECD Guideline No. 474 and the China National Standards for Food Safety (GB 15,193.5–2014). More details of this method can be found in the references that we established (Liu et al. 2019; Zeng et al. 2020; Chen et al. 2021). The induction of MN in peripheral blood RET was determined on days − 1, 4, 14, and 28. About 20 µL blood from each tube was transferred to new tubes (containing 4 µL CD71-FITC, 2.5 µL CD45-PE, 91.5 µL PBS, and 2 µL FBS). After incubating for 30 min, each tube was transferred to new tube containing 10 mL PBS, followed by centrifugation at 300 g/min for 5 min. The resulting supernatants were removed, and cells were resuspended, then labeled with 1 mL nucleic acid dye solution (20 μM/L DRAQ5). After incubating for 30 min at 37 ℃, specimens were centrifuged and aspirated as previously described twice. Samples were placed on ice until flow cytometric analysis (within 4 h). BD FACSVerse flow cytometer running the BD FACSuite Software Bundle v1.0 (BD Biosciences, NJ, USA) was used for data acquisition and analysis. The analysis indexes were the number of micronucleus in reticulocytes (MN-RET). Approximately 20,000 CD71-positive RETs per blood sample were evaluated for the presence of micronuclei. The %RETs for each sample were recorded as an index of a cytotoxicity to the hematopoietic system.

### Bone marrow micronucleus assay

The induction of MN in bone marrow RET was determined on day 29. Using a syringe and needle, marrow was flushed from the bone cavity with fetal bovine serum (FBS). Followed by centrifugation at 300 g/min for 5 min, the resulting supernatants were removed. Approximately 20 µL marrow from each tube was transferred to new tubes (containing 5 µL CD45-PE, 4 µL CD71-FITC, 89 µL PBS, and 2 µL FBS). The rest of the procedures were the same as described for peripheral blood micronucleus assay.

### Alkaline comet assay in blood leukocytes, liver

Refer to the guideline of the Organization for Economic Co-operation and Development (OECD 2016). Blood samples for comet assays were collected 3 h after treatment on days 4 and 28. Approximately 25 µL of per blood samples was mixed with 400 µL 0.5% low melting agarose and applied to glass microscope slides (three slides/animal) that were previously coated with 1% normal melting agarose. The slides were kept at 4 ℃ for 10 min to solidify and then applied the 0.5% low melting agarose to solidify again. After removing the coverslips, the slides were immersed in cold lysis solution (2.5-M NaCl, 100-mM Na_2_EDTA, 10-mM Tris base, 200-mM NaOH, pH 10) overnight. After lysis, slides were washed by cold ddH_2_O three times, followed by unwinding for 30 min at 4 ℃ in the dark with cold electrophoresis buffer (300 mM NaOH and 1 mM Na_2_EDTA; pH > 13). Electrophoresis was conducted for 30 min at 300 mA, 24 V. After electrophoresis, the slides were removed and washed with neutralization buffer in three changes, then dehydrated with 200-proof ethanol, air dried, and stored at room temperature.

Livers (the left lateral lobe) were sampled during necropsy, 3 h after the last treatment on day 29. A small left liver lobe was excised and put in the mincing solution (20 mM Na_2_EDTA, 10% DMSO, and 89% HBSS) and immediately cut into small pieces to release the cells. The supernatant (200 µL) was transferred into a tube containing 150 µL PBS, then mixed with 300 µL 1% low melting agarose and applied to prepare glass microscope slides. The procedures of solidification, lysis, unwinding, electrophoresis were as previously described. The only difference from the above procedure was the time for unwinding, i.e., 20 min.

A total of 150 cells per animal (50 cells/slide) were scored using the Comet Assay Software Project (CASP, University of Wroclaw, Poland). %Tail DNA (the tail DNA intensity) was calculated from the 150 cells, and group averages were reported.

### Statistical analyses

Statistical analyses were implemented in SPSS v22.0. The frequencies of all endpoints and issues per detected timepoint were expressed in the form of $\overline{x }$±s (mean value ± standard deviation). The data of body weight, hematology parameters, and the ratio of RETs were performed for homogeneity of variance test (Levene’s test, *P* < 0.05). When homogeneity was observed, statistical comparisons of control and treated groups were performed using one-way analysis of variance (ANOVA). When homogeneity was not observed, the Kruskal–Wallis H was performed. When significance was observed, Dunnett *t* test used to compare each group to the corresponding vehicle control (alpha = 0.05, two-sided tests). After the data of RBC^CD59−^, RET^CD59−^, MN-RETs, and TI log (10) transformed, a nonparametric one-way analysis of variance (ANOVA) was performed on all data, followed by Dunnett *t* test to compare each group to the corresponding vehicle control (alpha = 0.05, one-sided tests). To avoid zero values, the values of zero were replaced by 0.1 before analysis. Linear trend analyses (ANOVA for trend test) were performed for all data, and the level of significance was set at *P* < 0.05.

### Determination of PoDs metrics

No observed genotoxic effect level (NOGEL) and the benchmark dose (BMD) approaches were used to derive the PoDs for the all qualified endpoints on all timepoints. The NOGEL was defined as the highest dose level that did not show statistically significant increase in mutations using Dunnett’s approach. BMD is calculated by the PROAST Software (version 70.1, developed by the Dutch National Institute for Public Health and the Environment, RIVM). Model averaging was used in PROAST reduce selection bias. The BMDL (the lower 95% confidence limit of BMD) was calculated for each data set and can be used as the PoD. The CES is a percent change in the mean response and is roughly comparable to the benchmark response in the US EPAs BMD software. Several ways of deriving CES, such as CES 0.05, 0.1, and 0.5 (corresponding to 5, 10, and 50% increase over control) as well as CES 1SD (i.e., an increase of one standard deviation of the concurrent controls), were used (Zeller et al. 2017; Gi et al. 2018; White et al. 2020). All included data are continuous. If data points with value zero occurred, a constant of 0.1 was added to values, and covariates were not considered for the PROAST analysis. A model result is excluded from consideration where the BMDL values appear unreliable, such as when the BMDU/BMDL ratio is > 100, the BMDL value is estimated as greater than the BMDU, BMDL is zero, or BMDU is infinity.

## Results

### General condition and body weight

**Fig. 2:**
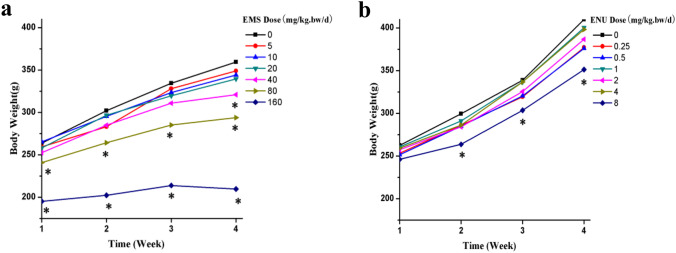
Body weight of rats

General toxicity was evaluated based on body weight (Fig. 2) over the treatment period and clinical observations (data not shown). In ENU groups, the average body weight in all dose groups increased during the administration. Starting from second week, a statistically significance decrease was observed in the treatment group of 8 mg/kg/day compared with the data of the control group (*P* < 0.05). No other treatment-related clinical signs or abnormality was observed in all groups.

In EMS groups, decreased activity, shed and disordered coat, dried fecal pellet were observed among rats in the treatment groups of 80 and 160 mg/kg/day starting from the third week. Starting from the first week, significantly decreased body weight compared with the data of the control group were observed in the treatment groups of 80 and 160 mg/kg/day (*P* < 0.05). At the fourth week, statistical significance could be observed starting from the group of 40 mg/kg/day, compared with the data of the control group (*P* < 0.05), and weight loss was observed in the treatment groups of 160 mg/kg/day.

### Hematology examination

Results showed that ENU affected the hematology parameters including RBC, HGB, and MCHC. RBC of rats in the treatment groups of 4 and 8 mg/kg/day and HGB of rats in 8 mg/kg/day dose group were decreased significantly compared with the data of the control group (*P* < 0.05). The MCHC of rats in the 2, 4, and 8 mg/kg/day dose groups was increased significantly compared with the value of the control group (*P* < 0.05). No statistical significance was observed for other parameters.

Results showed that EMS affected all hematology parameters, mostly with dose-dependent relationship (*P* < 0.05). RBC, Hb, HCT of 160 mg/kg/day dose group were decreased significantly compared with the values of the control group (*P* < 0.05). MCV, MCH, RDW-SD of 160 mg/kg/day dose groups were increased significantly compared with the data of the control group (*P* < 0.05). The MCHC from 20 mg/kg/day dose group, RDW-CV from 80 mg/kg/day dose group were increased significantly compared with the data of the control group (*P* < 0.05). WBC was decreased significantly starting from 20 mg/kg/day dose group compared with the data of the control group (*P* < 0.05). GPR was increased significantly starting from 40 mg/kg/day dose group compared with the data of the control group (*P* < 0.05). LPR was decreased significantly starting from 80 mg/kg/day dose group compared with the data of the control group (*P* < 0.05). The platelet-related parameters including PLT, PCT, MVP, and PDW were decreased significantly in the treatment groups of 80 and 160 mg/kg/day compared with the data of the control group (*P* < 0.05). P-LCR was decreased significantly in 160 mg/kg/day dose group compared with the data of the control group (*P* < 0.05).

Further details of the hematology parameters are shown in Supplementary material 1.

### Pig-a gene mutation assay

**Fig. 3:**
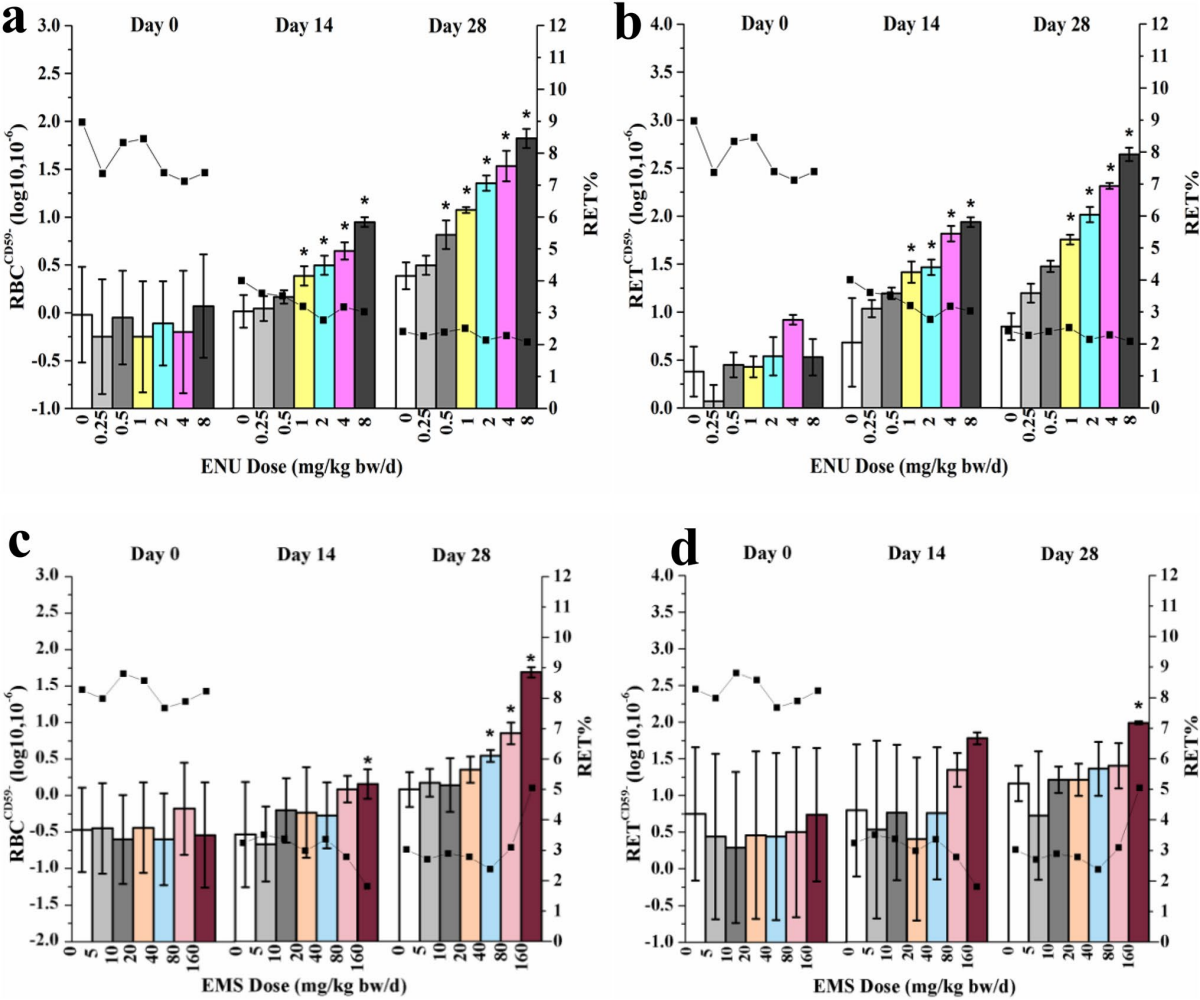
Results of Pig-a gene mutation assay. **a** Results of mutant RBC in *Pig-a* gene mutation assay of ENU as a function of time (*P < 0.05), column: RBC^CD59−^ (mutant red blood cells), line: RET% (the proportion of RETs in total RBCs); **b** results of mutant RET in *Pig-a* gene mutation assay of ENU as a function of time (*P < 0.05), column: RBC^CD59−^, line: RET%; **c** results of mutant RBC in *Pig-a* gene mutation assay of EMS as a function of time (*P < 0.05), column: RBC^CD59−^, line: RET%; **d** results of mutant RET in *Pig-a* gene mutation assay of EMS as a function of time (*P < 0.05), column: RBC^CD59−^, line: RET%

Results of genotoxicity tests for ENU are shown in Fig. 3a, b. On day 14, the RBC^CD59−^ and RET^CD59−^ of 1 to 8 mg/kg/day dose groups were increased significantly compared with the data of the control group (*P* < 0.05). On day 28, the RBC^CD59−^ of 0.5 to 8 mg/kg/day dose groups and the RET^CD59−^ of 1 to 8 mg/kg/day dose groups were increased significantly compared with the data of the control group (*P* < 0.05). On day 14, the %RET of 1 to 8 mg/kg/day dose groups was decreased significantly compared with the data of the control group (*P* < 0.05). On day 28, the %RET of each dose group still slightly decreased, but without statistical difference.

Results of EMS groups are shown in Fig. 3c, d. On day 14, statistical significance could be observed with RBC^CD59−^ and RET^CD59−^ at 160 mg/kg/day dose group compared with the data of the control group (*P* < 0.05), and RBC^CD59−^ increased distinctly compared with the values in the vehicle control group (*P* < 0.05). On day 28, a statistically significant increase of RBC^CD59−^ was observed in 40 mg/kg/day dose group compared with the data of the control group (*P* < 0.05), and RET^CD59−^ was observed in 160 mg/kg/day dose group compared with the data of the control group (*P* < 0.05). %RETs were dose-dependent decreased compared with the data of the control group (*P* < 0.05). On day 14, a statistically significant decrease of %RETs was observed in 160 mg/kg/day dose group compared with the data of the control group (*P* < 0.05). Probably due to the compensation of bone marrow hematopoietic regulation on day 28, %RETs of 160 mg/kg/day dose group were increased significantly compared with the data of the control group (*P* < 0.05).

### Micronucleus assay

**Fig. 4:**
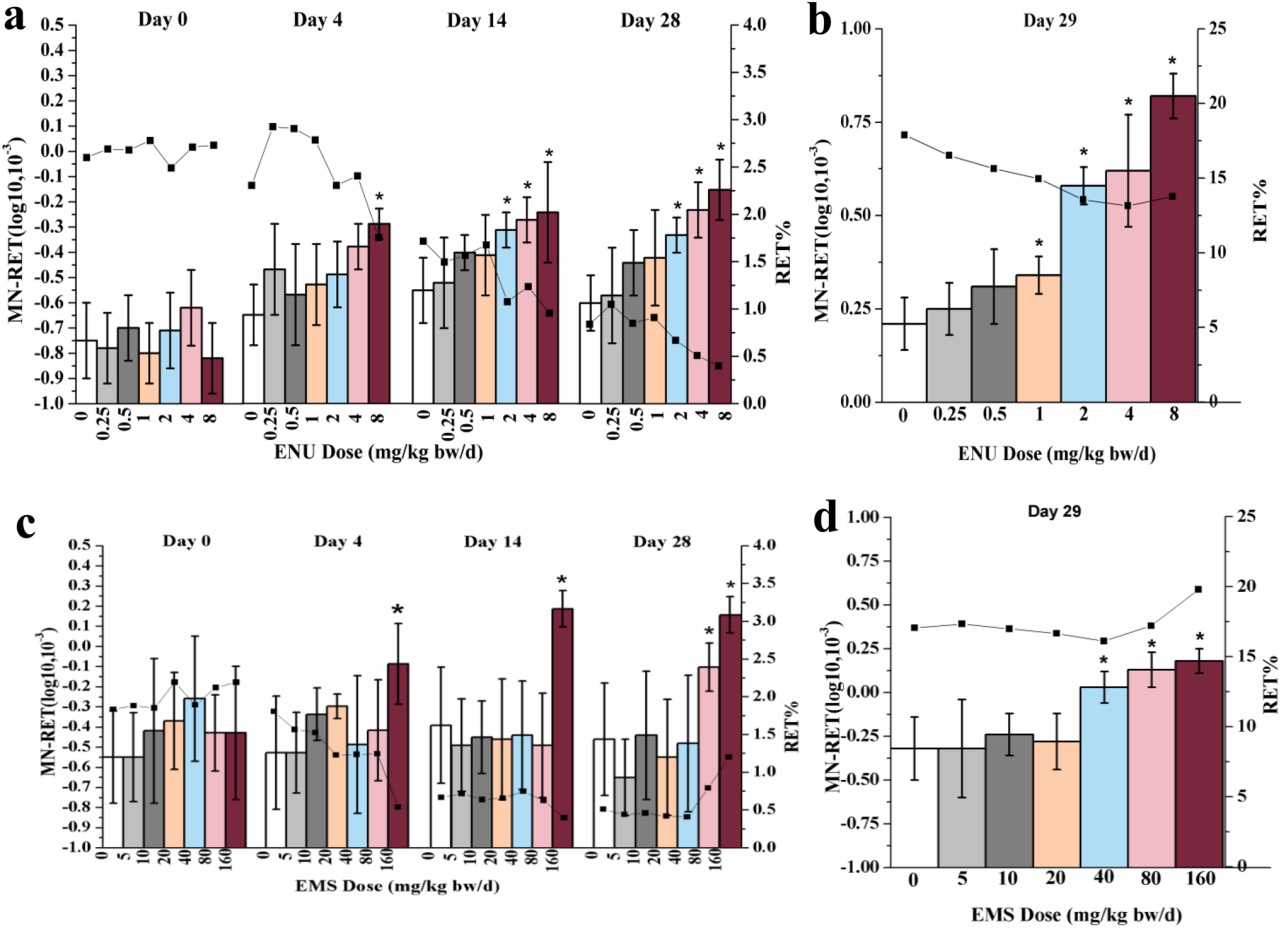
Results of micronucleus assay. **a** Results of MN-RET (the number of micronucleus in reticulocytes) in micronucleus assay of ENU as a function of time (*P < 0.05), column: MN-RET, line: RET% (the proportion of RETs in total RBCs); b results of MN-PCE (the number of micronucleus in polychromatic erythrocyte) in micronucleus assay of ENU as a function of time (*P < 0.05), column: MN-RET, line: RET%; c results of MN-RET in micronucleus assay of EMS as a function of time (*P < 0.05), column: MN-RET, line: RET%; d results of MN-PCE in micronucleus assay of EMS as a function of time (*P < 0.05), column: MN-RET, line: RET%

Results of MN-RETs, %RETs of ENU were illustrated in Fig. 4a. On day 4, MN-RET of 8 mg/kg/day dose group had a significant increase compared with the data of the control group (*P* < 0.05). On days 14 and 28, MN-RET of 2, 4, and 8 mg/kg/day dose groups had a significant increase compared with the data of the control group (*P* < 0.05). %RET weakly decreased over time. %RET on day 14 compared with the data of the control group had a significant decrease starting at 2 mg/kg/day dose group, and on day 28 starting at 4 mg/kg/day dose group. Data of MN-PCE (the number of micronucleus in polychromatic erythrocyte) was illustrated in Fig. 4b. On day 29, the MN-PCE of 1 to 8 mg/kg/day dose group had a significant increase compared with the data of the control group, and the %RET of 2 to 8 mg/kg/day dose groups had a significant decrease compared with the value of the control group.

The MN-RETs, %RETs of EMS were illustrated in Fig. 4c. On days 4 and 14, MN-RET of 160 mg/kg/day dose group had a significant increase compared with the data of the control group (*P* < 0.05). On day 28, MN-RET of 80 and 160 mg/kg/day dose groups had a significant increase compared with the data of the control group (*P* < 0.05). %RET on day 4 had a significant decrease starting at 40 mg/kg/day dose group compared with the data of the control group (*P* < 0.05). As the background level of %RET decreased with age of animal, and hematopoietic system may adapt to chemicals, the decline trend of %RET slowed down on day 14, only 160 mg/kg/day dose group decreased significantly compared with the data of the control group (*P* < 0.05), and on day 28, %RET compared with the data of the control group increased significantly due to compensation (*P* < 0.05). The MN-PCE of EMS was illustrated in Fig. 4d. On day 29, the MN-PCE starting at 40 mg/kg/day dose group had a significant increase, and the %RET at 160 mg/kg/day dose group had a significant increase, compared with the data of the control group (*P* < 0.05).

### Comet assay

**Fig. 5:**
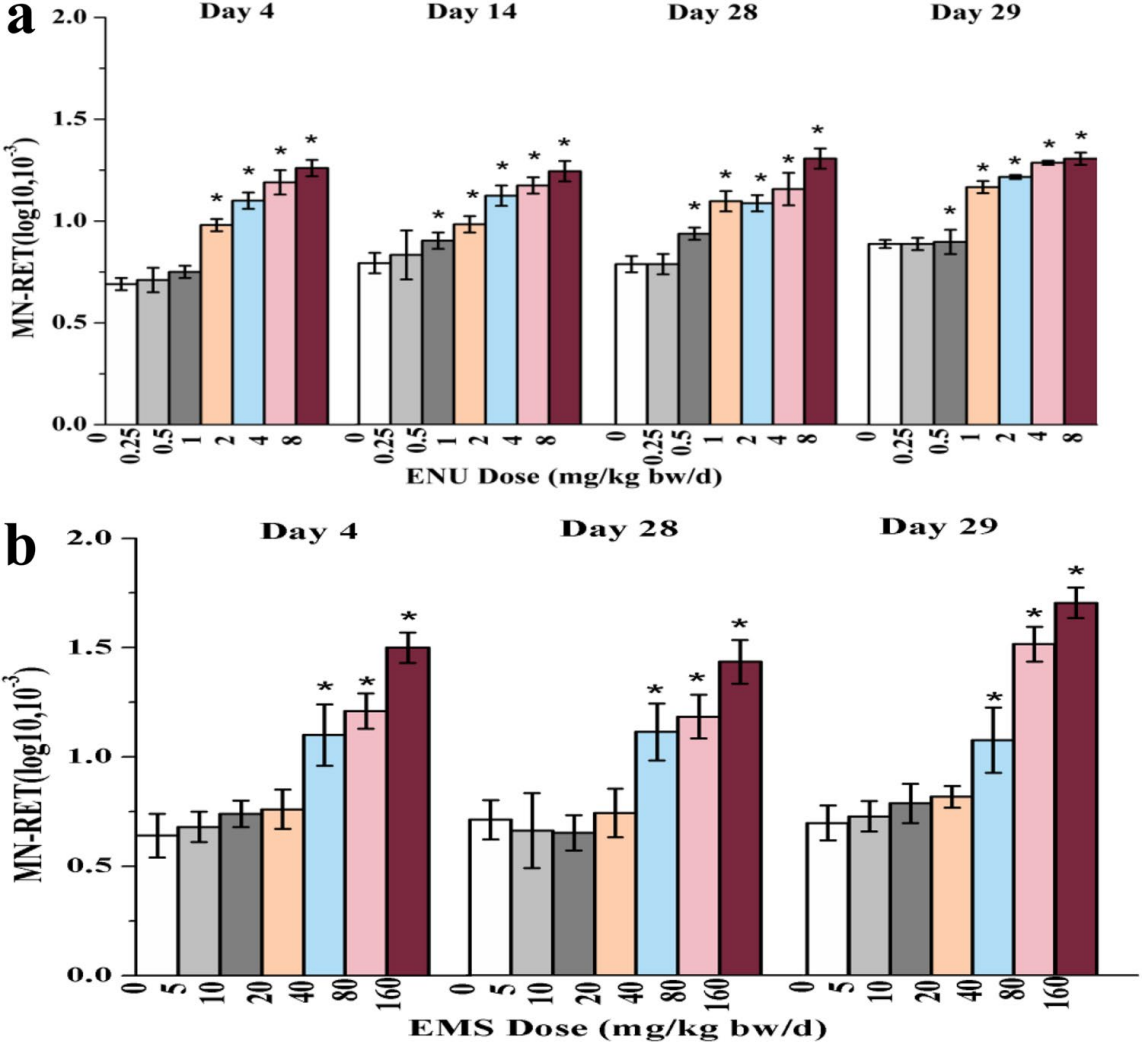
Results of comet assay. **a** Results of %Tail DNA (the tail DNA intensity) in comet assay of ENU as a function of time (*P < 0.05), column: %Tail DNA, line: RET% (the proportion of RETs in total RBCs); b results of %Tail DNA in comet assay of EMS as a function of time (*P < 0.05), column: %Tail DNA, line: RET%

In ENU groups (Fig. 5a), the peripheral blood %Tail DNA of 1 to 8 mg/kg/day dose groups had a significant increase compared with the data of the control group on day 4 (*P* < 0.05), and on day 14 and 28, the %Tail DNA of 0.5 to 8 mg/kg/day dose groups had a significant increase compared with the data of the control group (*P* < 0.05). On day 29, the liver %Tail DNA of 0.5 to 8 mg/kg/day dose groups had a significant increase compared with the data of the control group (*P* < 0.05).

In EMS groups (Fig. 5b), on day 4 and 28, a dose-dependent increase could be observed with %Tail DNA starting at 40 mg/kg/day dose group compared with the data of the control group (*P* < 0.05). On day 29, the liver %Tail DNA starting at 40 mg/kg/day dose group had a significant increase compared with the data of the control group (*P* < 0.05).

### PoDs metrics

Results of PoDs metrics were illustrated in Tables 1 and 2. For ENU groups, the minimum NOGEL was 0.25 mg/kg bw/day (comet assay of peripheral blood cell %Tail DNA on day 14 and 28; comet assay of liver cell %Tail DNA on day 29; *Pig-a* gene mutation assay of peripheral blood RBC^CD59−^ on day 28). The highest NOGEL was 4 mg/kg bw/day (micronucleus assay of peripheral blood RET^CD59−^ on day 4). For EMS groups, the minimum NOGEL was 20 mg/kg bw/day (comet assay of peripheral blood cell %Tail DNA; *Pig-a* gene mutation assay of peripheral blood RBC^CD59−^ on day 28; micronucleus assay of bone marrow RET^CD59−^ on day 29). The highest NOGEL was 160 mg/kg bw/day (*Pig-a* gene mutation assay of peripheral blood RET^CD59−^ on day 14).

**Table 1:** Summary on PoD values of genotoxic endpoints of ENU

Tests	Target endpoint	Timepoint	PoD (mg/kg bw/d)				
			NOGEL	BMDL_0.05CES_^a^	BMDL_0.1CES_	BMDL_0.5CES_	BMDL_CES1SD_
*Pig−a* gene mutation assay	Blood RBC	14	0.5	0.003	0.011	0.17	0.094
		28	0.25	0.0094	0.021	0.12	0.19
	Blood RET	14	0.5	NT	NT	NT^b^	0.87
		28	0.5	NT	NT	0.0036	0.66
Micronucleus assay	Blood RET	4	4	NT	NT^c^	0.15	NT
		14	1	NT	NT	0.13	NT
		28	1	NT	NT	0.16	NT
	Bone marrow RET	29	0.5	0.01	0.032	0.45	0.14
Comet assay	Blood cell	4	0.5	0.11	0.18	0.55	0.34
		14	0.25	0.038	0.087	0.56	0.85
		28	0.25	0.004	0.018	0.34	0.32
	Liver cell	29	0.25	0.17	0.25	0.62	0.52

Abbreviations: *PoD* points of departure, *RBC* red blood cell, *RET* reticulocytes, *NOGEL* no observed genotoxic effect level, *BMDL* the lower bound of benchmark dose, *CES* critical effect sizes

^a^Raw data was used for BMDL fitting. CES set at 0.05, 0.1, 0.5, and 1SD. NT: Averaging model failed to fit (*P* < 0.1)

^b^BMDU/BMDL = 223

^c^BMDU/BMDL = 259 × 10^2^

**Table 2:** Summary on PoD values of genotoxic endpoints of EMS

Tests	Target endpoint	Timepoint	PoD (mg/kg bw/d)				
			NOGEL	BMDL_**0.05CES**_^**a**^	BMDL_**0.1CES**_^**b**^	BMDL_**0.5CES**_^**c**^	BMDL_**CES1SD**_^**d**^
*Pig-a* gene mutation assay	Blood RBC	14	40	NT	NT	NT^b^	2.1
		28	20	1.1	2.3	12	14
	Blood RET	14	160	NT^c^	NT^d^	1.7	53
		28	80	NT^e^	0.3	4.7	110
Micronucleus assay	Blood RET	4	80	3.8	12	52	25
		14	80	19	27	63	43
		28	40	1.1	2.6	16	7.9
	Bone marrow RET	29	20	0.99	2.6	15	5.8
Comet assay	Blood cell	4	20	0.86	1.9	11	22
		28	20	1	2.1	13	23
	Liver cell	29	20	6.2	8.9	22	28

Abbreviations: *PoD* points of departure, *RBC* red blood cell, *RET* reticulocytes, *NOGEL* no observed genotoxic effect level, *BMDL* the lower bound of benchmark dose, *CES*. critical effect sizes

a Raw data was used for BMDL fitting. CES set at 0.05, 0.1, 0.5, and 1SD. NT: Averaging model failed to fit (*P*. <0.1)

b.BMDU / BMDL =12×10^2^

c. BMDU / BMDL =893

d. BMDU / BMDL =347

e. BMDU / BMDL =424

BMDL with CES set at 0.05, 0.1, and 0.5 was always lower than the corresponding NOGELs in all endpoints. BMDL with CES set at 1SD varied greatly, even marginally exceeds the NOGEL. The analysis details with the PROAST of ENU and EMS were shown in Supplementary material 2.

## Discussion

Recently, the focus of genotoxic assessment has moved from “qualitative” to “quantitative.” For quantitative genotoxic risk assessment, the IWGT Working Group on Quantitative Approaches to Genetic Toxicology Risk Assessment (QWG) recommended the use of benchmark dose (BMD) over no observed genotoxic effect level (NOGEL) for PoD calculation (Macgregor et al. 2015a, b). The QWG also highlighted the importance of the selection of endpoints and tissues in vivo, considering metabolism, exposure, and genotoxic mode of action. Therefore, we used an in vivo multi-endpoint genotoxicity assessment platform, which not only measured the dose–response relationship between each endpoint and tissue quickly and easily, but also met the requirement for a high efficiency and high sensitivity assay for in vivo genotoxicity tests. Then, we quantitative analyzed dose–response relationship and the potential thresholds to define genotoxicity PoDs using the updated BMD software PROAST with model averaging.

Following 28 days of treatment, positive genotoxic responses that ENU and EMS induced were observed in all endpoints and tissues evaluated. Consistent effects were observed in comet assay for any given timepoint and doses, while endpoint parameters in MNT and *Pig-a* assay presented a dose- and time-dependent increase. Comet test was mainly used to detect DNA damage within a short period of time, consistent results indicating that low-dose repeated exposure did not affect the ability of comet assay to detect DNA damage (Tice et al. 2000). But DNA damage detected by comet assay could be repaired before it is converted to heritable DNA damage. The test is of limited reference when used to extrapolate the carcinogenicity risks of chemicals to humans; therefore, it is not used as the main reference when calculating the threshold (International Council on Harmonisation of Technical Requirements for Pharmaceuticals for Human Use 2011).

Micronucleus test was mainly used to detect chromosome breakage and aneuploidy. Bone marrow or peripheral blood could be the detection object based on the development of flow cytometry. Bone marrow cells have the advantages of low spontaneous mutation rate and good stability (Tice et al. 2000), and the peripheral blood can collect target cells repeatedly at different timepoints, which is more feasible to the integration of micronucleus test with other genotoxicity tests and general toxicity tests. In our study, ENU groups observed a significant increase in 8 mg/kg bw dose group on day 4 compared with the data of the control group, while on days 14 and 28, statistical significance showed began in 2 mg/kg bw dose group. For EMS, a significant increase was observed in 160 mg/kg bw dose group on day 4 and 14 compared with the data of the control group, while on day 28, statistical significance showed began in 80 mg/kg bw dose group. This may be caused by two reasons: (1) hyperactivity of hematopoiesis was aroused with consequent elevation of micronuclei; (2) the generation rate of micronucleus was faster than its disappearance rate after repeated dose exposure. However, some researchers found that micronucleus was difficult to observe for some chemicals, such as Mitomycin C, after long-term exposure due to tissue tolerance. Therefore, short-term and long-term detection timepoints for micronucleus test are both significant for different purposes (Hamada et al. 2001).

*Pig-a* mutation assay was mainly used to detect gene mutation. The method using three dyeing method (CD59-APC/CD61-PE/SYTO13) had higher sensitivity and with low background mutation rate. It is suitable for genotoxicity evaluation on low-dose exposure, so it is beneficial to identify the linear or sub-linear dose–response relationship of genotoxicity substances (Huo et al. 2017, 2019). The mutant frequency of RET^CD59−^ in low-dose group of ENU and EMS was observed to increase at earlier time compared with the data of the control group, but not statistically significant. The results showed that RBC^CD59−^ was more sensitive and stable at all detection timepoints. This might be due to the update cycle of RET that needs several days, its mutation level mainly reflects the genotoxicity in the short term (Dobrovolsky et al. 2020). The updation cycle of RBC needs several weeks, thus covering mutational events over a longer duration. And a higher number of RBCs was evaluated for the mutant frequency relative to RETs (approximately 1 × 10^7^ versus 3 × 10^5^). Therefore, the mutant rate of RBCs often has a higher sensitivity and advantage when applied to low-dose effects, which has been demonstrated by several studies (Zeller et al. 2016; Guérard et al. 2017).

In this study, two quantitative methods were used to derive the threshold of genotoxicity, NOGEL and BMD. About NOGEL, the PoDs of different genotoxicity endpoints obtained range from 0.25 to 4 mg/kg bw for ENU, 20 mg/kg bw to 160 mg/kg bw for EMS. The range was rather wide, which was highly dependent on the dose design and limited to statistical analyzing ability.

BMD was calculated as the dose at which a particular response level change relative to background was imputed based on specific software and models (Haber et al. 2018). The choice of the critical effect size (CES), also known as the benchmark response (BMR), is crucial for the results obtained through PROAST. Several ways of deriving CES, such as CES 0.05, 0.1, and 0.5 (corresponding to 5, 10, and 50% increase over control) as well as CES 1SD, were used which is primarily recommended based on the non-genotoxic endpoints rather than of genotoxicity (Haber et al. 2018). For non-genotoxic endpoints, multiple recommendations to set CES have been published (Zeller et al. 2016; Gollapudi et al. 2020). For example, for continuous toxicological endpoints such as body weight and hematological parameters, the European Food Safety Authority (EFSA) recommends a CES of 5%, and the United States Environmental Protection Agency (USEPA) recommends a CES of 1SD from the control mean. For quantal endpoints such as cancer, EFSA and USEPA recommend a CES of 10% (The European Food Safety Authority 2009; The United States Environmental Protection Agency 2012). However, for genotoxic endpoints such as mutation and chromosome damage, the situation is more complex, and there is still no consensus about which CES values should be applied to best reflect the dose–response relationship in the different type of genotoxicity test (Zeller et al. 2017).

Under this experimental condition, the BMDL with CES at 0.05, 0.1, 0.5, 1SD obtained ranges from 0.003 to 0.87 mg/kg bw for ENU, 0.3 to 110 mg/kg bw for EMS. We observed the results of BMDL at the same endpoints and timepoints increased with the CES at 0.05, 0.1, 0.5. But the results of BMDL with CES1SD were typically much closer to the NOGEL, even marginally exceed the NOGEL, and could be higher or lower than BMDL with 0.5CES, even lower than 0.1CES. This might be due to deriving CES from one standard deviation of the concurrent vehicle controls. Among different genotoxicity endpoints, the lowest and highest CES1SD were 0.058 and 9.39 for ENU, 0.21 and 10.51 for EMS. The data demonstrated that little changes of variability of the controls can have considerable impact on the results of dose–response modeling. We could see in Fig. 5, the results of %Tail DNA in comet assay were with low response variability, and the results of BMD _CES1SD_ were more consistent and higher, whereby it is unlikely that a 1SD change from control could be deemed adverse. Conversely, for endpoints with high control variability, the 1SD approach will yield larger CES values, that is, the percentage increase corresponding to a 1SD increase above control will be relatively higher. Higher CES values will yield higher BMD values, which may be less suitable for regulatory. Therefore, from this point of view, 1SD is not suitable for genetic endpoint calculation of BMD.

In the same choice of CES, lower PoD values were obtained in peripheral blood *Pig-a* assay compared to those obtained in the MNT and comet assay, such as the BMDL_0.05CES_ of 0.003 mg/kg bw in *Pig-a* assay (RBC, day 14, ENU) vs. BMDL_0.05CES_ of 0.038 mg/kg bw in comet assay (blood, day 14, ENU). This might be due to the fact that the test has a relatively high sensitivity and could detect adverse effects at low doses, at the same time having a very low spontaneous background values, and can increase several dozens or even 100-fold over the negative control group. We also observed that, for some endpoints in micronucleus test and *Pig-a* assay in this study, the detected values showed less than twofold increase at the low dose compared with control groups. Further analysis showed that CES of 5 or 10% for these endpoints was within the CES1SD values for historical data, which means that they were almost undistinguishable from the background noise (data not shown). This was consistent with the previous analysis that Zeller et al. (2017) and Guérard et al. (2017) demonstrated that increases of 5 or 10% over mean controls are over-conservative CES levels as they do not reflect the performance for genetic mutations endpoints.

The ratio of BMDL and BMDU values reflects the precision of the BMD and is conducive to regulatory decision-making (Benford 2016). White et al. (2019) noted that large BMDU/BMDL ratios (e.g., > 100) suggest that the results may not be suitable for regulatory use. In this study, the ratios of BMDU/BMDL are usually below 50 except for RET^CD59−^ on day 14 (BMDL_0.5CES_ = 223), %MN-RET on day 4 (BMDL_0.1CES_ = 259 × 10^2^) for ENU, and RBC^CD59−^ on day 14 (BMDL_0.5CES_ = 12 × 10^2^), RET^CD59−^ on day 14 (BMDL_0.05CES_ = 893, BMDL_0.1CES_ = 347) and on day 28 (BMDL_0.05CES_ = 424), indicating lower BMD precision for these endpoints.

At present, there were few studies on the possible genotoxicity thresholds under low-dose exposure in vivo of ENU and EMS (Delft et al. 1998; Gocke and Wall 2009; Bhalli et al. 2011; Dobo et al. 2011; Cao et al. 2014; O'Brien et al. 2015)*.* Relevant results were summarized in Tables 3 and 4. It could be observed that the genotoxicity threshold results of ENU and EMS obtained in different studies were different, which might be caused by different species, animal strains, detection tissues, genotoxic endpoints, statistical methods, and so on. Similar to Dobo’s study (Dobo et al. 2011), we got the same genotoxicity thresholds of ENU with NOGEL in same breed rat and same genotoxicity endpoint. It was also found that the genotoxic thresholds of ENU and EMS in rats were slightly lower than that in mice. This might be caused by species difference. Some researchers have found that the DNA damage of rats is more serious at the same dose when compared with that in mice (Gerson et al. 1986); in other words, rats are more sensitive to mutagenic induction of ENU and EMS. It also can be observed that the thresholds of gene mutation were generally lower than that of micronucleus. This was related to the mode of action (MOA) of ENU and EMS. ENU mainly forms O^6^-alkylguanine (O^6^AlkG) adducts by interacting with DNA, resulting in gene mutation, while EMS forms mainly N^7^-alkylguanine (N^7^AlkG) adducts (Gerson et al. 1986; Kaina et al. 2007). O^6^AlkG is a DNA adduct most likely to cause gene mutation, which also leads to a relatively low genotoxicity threshold of ENU in this study.

**Table 3:** Summary of genetic toxicity thresholds in vivo of ENU

Reference	Animals	Tissue	Endpoint	PoD (mg/kg bw/d)	
				NOGEL	BMDL
Delft et al	lacZ transgenic mice	Intestine	lacZ _gene mutation_	10	5.46
			Dlb-1 _gene mutation_	_**NT**_^a^	1.55
		Spleen	lacZ _gene mutation_	25	11.7
Bhalli et al	C57BL/6	Peripheral blood	^Pig−a^_gene mutation RET_	10	1.46
	mice		^Pig−a^_gene mutation RBC_	45	0.95
			Micronucleus RET	10	4.03
O'Brien et al	Muta™ mice	Testis	lacZ _gene mutation_	2	1
Dobo et al	SD _Rat_	Peripheral blood	^Pig−a^_gene mutation_	0.25	-

Abbreviations: *RBC* red blood cell, *RET* reticulocytes, *NOGEL* no observed genotoxic effect level, *BMDL* the lower bound of benchmark dose

^**a**^NT: All model failed to fit ( *P* < 0.1)

**Table 4:** Summary of genetic toxicity thresholds in vivo of EMS

Reference	Animals	Tissue	Endpoint	PoD (mg/kg bw/d)	
				NOGEL	BMDL
Cao et al	^gpt−^delta mice	Lung	^gpt^ gene mutation	5	0.56
		Kidney	^gpt^ gene mutation	5	0.59
		Spleen	^gpt^ gene mutation	13	0.35
		Bone marrow	^gpt^ gene mutation	20	0.37
		Small intestine	^gpt^ gene mutation	20	0.55
		Liver	Micronucleus	55	2.3
		Peripheral blood	^Pig−a^ gene mutation RET	5	1.18
			^Pig−a^ gene mutation RBC	20	4.67
			Micronucleus RET on 13d	20	6.79
			Micronucleus RET on 29d	55	8.26
Gocke et al	Muta_**TM**_mice	Bone marrow	^lac^Z gene mutation	50	9.29
		Liver	^lac^Z gene mutation	50	41
		Small intestine	^lac^Z gene mutation	25	12.3
	CD1 mice	Bone marrow	Micronucleus RET on 7d	80	58.7

Abbreviations: *RBC* red blood cell, *RET* reticulocytes, *NOGEL* no observed genotoxic effect level, *BMDL* the lower bound of benchmark dose

Genotoxicity data in risk assessment mainly focuses on the extrapolation from the PoD in order to establish acceptable human exposure levels. When selecting the appropriate threshold for human genotoxicity risk extrapolated by PoD, the selection should be based on animals, genetic endpoints, possible human exposure patterns, and mode of action of the chemical. And in order to adequately protect humans against all adverse outcomes, usually the lowest reference point was recommended which is both conservative and protective on the basis of biological relevance. Based on the above discussion, PoD values for genotoxicity could be derived according to the following points: (1) the choice of CES1SD may not be suitable for genetic endpoint calculation of BMD; (2) comet test often used to indicate whether chemicals can interact directly with DNA and limited use in extrapolating the carcinogenicity risks of chemicals to humans; (3) the CES parameters of 0.05 and 0.1 could be not suitable for BMD calculations in genotoxicity tests; (4) the MOA of ENU and EMS mainly forms DNA adduct which can cause genetic mutations. Thus, it could be seen the relatively lower PoD values of ENU and EMS could be determined to be 0.0036 mg/kg bw based on RET^CD59−^ at Day 28 with CES0.5 and 1.7 mg/kg bw based on RET^CD59−^ at Day 14 with CES0.5, respectively.

Collectively, our study used the in vivo multi-endpoint genotoxicity assessment platform in rats to analyze the genotoxic dose–response relationship of ENU and EMS, followed by the estimation of the PoD values using NOGEL and BMD with different CES (0.05, 0.1, 0.5, and 1SD) with PROAST. And the PoD values of 0.0036 mg/kg bw for ENU and 1.7 mg/kg.bw for EMS were derived under the experimental conditions. It should be noted that PoDs varied across genetic endpoints, timepoints, and statistical methods. Considering both biological relevance and protection of human health, conservative results were selected, and the lowest BMDL was selected from the results calculated by multiple models of multiple endpoints. The choice of CES is critically important for determination of BMD values to be used for risk assessment and regulatory decision-making. Thus, it can be seen the validity of “one size fits all” approach is worthy of further investigation. Our study provides valuable data for quantitative genotoxic assessment of ENU and EMS as well as defining CES values of BMD of genotoxic endpoints.

## Supplementary Information

Below is the link to the electronic supplementary material.

Supplementary file1 (DOCX 20884 KB)

## Data Availability

Not applicable.
