# Supplementary material for: Determination of potential thresholds for N-ethyl-N-nitrosourea and ethyl methanesulfonate based on a multi-endpoint genotoxicity assessment platform in rats
Source: Environ Sci Pollut Res Int. 2022 Jul 6;29(56):85128–42. doi: 10.1007/s11356-022-21605-z (PMC9646607; doi:10.1007/s11356-022-21605-z)
Supplement: Supplementary file 1 — Supplementary file1 (DOCX 20884 KB) [file 11356_2022_21605_MOESM1_ESM.docx]

**Supplementary material 1: data associated with this article**

Table 1 Body weight of rats with ENU. ^a^

| **Doses (mg/kg.bw/d)** | **Body weight (g)** | | | |
| --- | --- | --- | --- | --- |
|  | D7 | D14 | D21 | D28 |
| 0 | 262.5±7.7 | 299.5±7.4 | 338.8±9.3 | 409.9±7.5 |
| 0.25 | 253.3±12.1 | 285.9±8.2 | 319.4±14.4 | 377.1±24.0 |
| 0.5 | 251.6±9.6 | 284.9±9.1 | 320.5±19.8 | 376.0±23.9 |
| 1 | 260.6±14.0 | 291.1±8.4 | 336.8±10.3 | 400.1±16.4 |
| 2 | 257.3±9.3 | 284.2±11.3 | 325.6±17.1 | 386.7±24.1 |
| 4 | 259.0±11.8 | 286.1±16.5 | 336.7±21.7 | 398.1±35.5 |
| 8 | 246.0±7.8 | 263.6±11.6 * | 303.5±14.0 * | 351.3±22.9 * |
| **P value for Trend** | 0.058 | 0.0001^#^ | 0.01^#^ | 0.005^#^ |

Abbreviation: ENU, N-ethyl-N-nitrosourea.

^a^ Mean ± S.D., n = 5.

* Statistically different from those in the vehicle control group (*P* < 0.05).

^#^ *P* < 0.05 in trend analysis.

Table 2 Body weight of rats with EMS. ^a^

| **Doses (mg/kg.bw/d)** | **Body weight (g)** | | | |
| --- | --- | --- | --- | --- |
|  | D7 | D14 | D21 | D28 |
| 0 | 263.7±9.2 | 302.0±12.2 | 334.5±12.5 | 359.5±7.9 |
| 5 | 259.3±11.7 | 283.1±22.8 | 328.1±15.8 | 349.0±14.9 |
| 10 | 265.2±15.6 | 295.5±11.8 | 323.5±9.8 | 344.0±13.7 |
| 20 | 258.2±11.8 | 296.8±13.6 | 319.7±14.4 | 339.4±18.1 |
| 40 | 252.6±11.8 | 284.9±13.9 | 310.9±13.2 | 321.0±13.5 * |
| 80 | 240.7±7.5 * | 264.1±8.9 * | 285.2±8.3 * | 293.8±7.3 * |
| 160 | 195.1±18.1 * | 202.2±18.9 * | 213.8±26.8 * | 209.7±13.1 * |
| **P value for Trend** | 0.0001^#^ | 0.0001^#^ | 0.0001^#^ | 0.0001^#^ |

Abbreviation: EMS, Ethyl Methanesulfonate.

^a^  Mean ± S.D., n = 5.

* Statistically different from those in the vehicle control group (*P* < 0.05).

^#^ *P* < 0.05 in trend analysis.

Table 3 Hematology parameters in rats treated with ENU. ^a^

| **Parameters** | **Doses（mg/kg.bw/d）** | | | | | | | *P* value for Trend |
| --- | --- | --- | --- | --- | --- | --- | --- | --- |
|  | 0 | 0.25 | 0.5 | 1 | 2 | 4 | 8 |  |
| WBC ( ×109/L ) | 10.24±0.49 | 10.23±0.7 | 9.58±1.37 | 9.13±0.66 | 8.73±1.29 | 8.85±1.19 | 9.10±0.73 | 0.0001^#^ |
| GPR ( % ) | 12.80±0.84 | 14.00±2.76 | 13.83±2.23 | 12.17±2.23 | 14.83±1.83 | 14.33±1.51 | 13.33±1.51 | 0.0001^#^ |
| LPR ( % ) | 84.60±1.52 | 83.83±1.72 | 84.00±2.45 | 86.17±2.04 | 83.83±2.14 | 83.83±0.98 | 85.17±2.23 | 0.0001^#^ |
| RBC ( ×109/L ) | 9.10±0.32 | 8.89±0.29 | 9.17±0.38 | 8.88±0.47 | 8.66±0.41 | 8.43±0.36 * | 8.39±0.23 * | 0.0001^#^ |
| HGB ( g/L ) | 187.80±2.95 | 183.83±7.19 | 186.33±7.87 | 181.00±10.55 | 182.83±3.76 | 179.33±5.13 | 174.00±4.34 * | 0.0001^#^ |
| HCT ( L/L ) | 48.02±1.99 | 49.10±2.24 | 51.08±2.41 | 48.78±3.31 | 48.15±0.96 | 49.98±2.11 | 48.53±2.18 | 0.0001^#^ |
| MCV ( fL ) | 54.40±0.97 | 54.42±1.55 | 54.90±1.44 | 55.38±1.52 | 55.03±1.37 | 56.05±1.60 | 56.42±1.63 | 0.0001^#^ |
| MCH ( pg ) | 20.04±0.66 | 20.13±0.73 | 20.32±0.65 | 20.35±0.56 | 20.72±0.52 | 21.10±0.74 | 20.38±0.86 | 0.0001^#^ |
| MCHC ( g/L ) | 362.60±5.90 | 364.33±3.01 | 364.83±3.06 | 367.67±0.82 | 379.67±4.13 * | 376.17±5.71 * | 372.33±3.88 * | 0.0001^#^ |
| RDW-CV ( % ) | 15.76±0.71 | 15.92±0.52 | 16.07±0.69 | 15.95±0.61 | 16.18±0.72 | 16.05±0.60 | 16.33±0.68 | 0.0001^#^ |
| RDW-SD ( fL ) | 25.52±0.30 | 25.68±0.88 | 25.68±0.64 | 25.40±0.97 | 25.83±1.43 | 25.98±0.96 | 26.08±1.05 | 0.0001^#^ |
| PLT ( ×109/L ) | 463.6±53.61 | 402.33±80.44 | 350.50±90.92 | 414.67±92.32 | 369.33±117.36 | 368.33±84.81 | 346.67±64.42 | 0.0001^#^ |
| PCT ( % ) | 0.38±0.04 | 0.33±0.11 | 0.29±0.11 | 0.28±0.04 | 0.30±0.06 | 0.30±0.14 | 0.26±0.11 | 0.0001^#^ |
| MPV ( fL ) | 7.86±0.29 | 8.30±0.38 | 8.23±0.34 | 8.13±0.53 | 7.92±0.39 | 7.63±0.35 | 7.57±0.23 | 0.0001^#^ |
| PDW ( % ) | 9.98±0.74 | 9.73±0.64 | 9.58±1.04 | 9.22±0.66 | 9.15±0.54 | 8.73±0.64 | 8.58±0.45 | 0.0001^#^ |
| P-LCR ( % ) | 22.62±4.14 | 21.42±3.45 | 20.97±5.66 | 18.62±3.53 | 18.25±2.62 | 16.08±3.30 | 15.55±2.25 | 0.0001^#^ |

Abbreviation: ENU, N-ethyl-N-nitrosourea. WBC, white blood cell; GPR, granulocyte percent rate; LPR, lymphocyte percent rate; RBC, red blood cell; Hb, hemoglobin; HCT, hematocrit; MCV, mean corpuscular volume; MCH, mean corpuscular hemoglobin; MCHC, mean corpuscular hemoglobin concentration; RDW-CV, mean corpuscular hemoglobin concentration; RDW-SD, red blood cell distribution width-standard deviation; PLT, platelet; MPV, mean platelet volume; PDW, platelet distribution width; P-LCR, platelet-large cell ratio.

^a^  Mean ± S.D., n = 5.

* Statistically different from those in the vehicle control group (*P* < 0.05).

^#^ *P* < 0.05 in trend analysis.

Table 4 Hematology parameters in rats treated with EMS. ^a^

| **Parameters** | **Doses (mg/kg.bw/d)** | | | | | | | *P* value for Trend |
| --- | --- | --- | --- | --- | --- | --- | --- | --- |
|  | 0 | 5 | 10 | 20 | 40 | 80 | 160 |  |
| WBC ( ×109/L ) | 9.88±1.21 | 9.82±0.66 | 8.65±1.07 | 7.13±0.86* | 5.08±0.94* | 3.73±0.50* | 2.87±0.61* | 0.0001^#^ |
| GPR ( % ) | 26.83±2.86 | 28.67±2.66 | 29.67±2.34 | 30.00±4.98 | 32.75±4.07* | 35.60±3.31* | 37.67±3.39* | 0.0001^#^ |
| LPR ( % ) | 71.83±2.86 | 69.50±3.15 | 68.83±2.14 | 68.50±6.02 | 66.23±4.54 | 61.35±4.77* | 59.83±3.25* | 0.0001^#^ |
| RBC ( ×109/L ) | 8.22±0.36 | 8.37±0.41 | 8.35±0.44 | 8.37±0.25 | 7.93±0.52 | 7.80±0.42 | 6.76±0.42* | 0.0001^#^ |
| HGB ( g/L ) | 160.00±5.97 | 158.00±8.15 | 163.00±5.69 | 159.33±5.75 | 164.00±6.63 | 162.33±7.53 | 142.67±2.42* | 0.0001^#^ |
| HCT ( L/L ) | 45.20±1.53 | 44.98±1.38 | 44.65±1.39 | 44.72±1.05 | 44.65±1.57 | 43.23±1.35 | 38.27±1.13* | 0.0001^#^ |
| MCV ( fL ) | 54.33±1.02 | 54.40±1.05 | 54.17±0.58 | 54.52±1.20 | 54.27±1.02 | 54.20±1.11 | 58.33±1.05* | 0.0001^#^ |
| MCH ( pg ) | 19.50±0.54 | 19.55±0.81 | 19.73±0.72 | 19.45±0.56 | 19.35±0.43 | 19.87±0.54 | 22.17±1.03* | 0.0001^#^ |
| MCHC ( g/L ) | 357.00±4.43 | 357.17±6.05 | 358.33±7.31 | 365.83±3.31* | 369.67±2.58* | 373.50±6.35* | 377.67±6.98* | 0.0001^#^ |
| RDW-CV ( % ) | 15.53±0.39 | 15.37±0.18 | 15.67±0.38 | 15.62±0.33 | 15.88±0.60 | 17.45±0.62* | 19.60±0.77* | 0.0001^#^ |
| RDW-SD ( fL ) | 24.37±0.49 | 24.20±0.54 | 24.30±0.51 | 24.10±0.42 | 24.22±0.42 | 24.58±0.32 | 27.65±1.89* | 0.0001^#^ |
| PLT ( ×109/L ) | 364.83±7.36 | 368.00±6.32 | 366.83±7.19 | 356.67±7.00 | 351.83±11.79 | 336.00±5.18* | 290.50±13.59* | 0.0001^#^ |
| PCT ( % ) | 0.29±0.01 | 0.29±0.01 | 0.29±0.02 | 0.29±0.02 | 0.29±0.01 | 0.27±0.01* | 0.23±0.02* | 0.0001^#^ |
| MPV ( fL ) | 7.62±0.10 | 7.62±0.15 | 7.62±0.10 | 7.55±0.08 | 7.50±0.06 | 7.38±0.04* | 7.25±0.10* | 0.0001^#^ |
| PDW ( % ) | 8.82±0.42 | 8.92±0.38 | 8.78±0.33 | 8.67±0.18 | 8.48±0.08 | 8.37±0.10* | 8.12±0.33* | 0.0001^#^ |
| P-LCR ( % ) | 15.93±0.99 | 16.30±1.18 | 16.15±1.17 | 15.88±0.87 | 15.23±0.62 | 14.78±0.80 | 13.53±1.32* | 0.0001^#^ |

Abbreviation: EMS, Ethyl Methanesulfonate. WBC, white blood cell; GPR, granulocyte percent rate; LPR, lymphocyte percent rate; RBC, red blood cell; Hb, hemoglobin; HCT, hematocrit; MCV, mean corpuscular volume; MCH, mean corpuscular hemoglobin; MCHC, mean corpuscular hemoglobin concentration; RDW-CV, mean corpuscular hemoglobin concentration; RDW-SD, red blood cell distribution width-standard deviation; PLT, platelet; MPV, mean platelet volume; PDW, platelet distribution width; P-LCR, platelet-large cell ratio.

^a^  Mean ± S.D., n = 5.

* Statistically different from those in the vehicle control group (*P* < 0.05).

^#^ *P* < 0.05 in trend analysis.

Table 5 *Pig-a* gene mutation assay with ENU. ^a^

| **Doses (mg/kg.bw/d)** | RBC**^CD59-^** ( log_10_, ×10**^-6^** ) | | | RET**^CD59-^** ( log_10_, ×10**^-6^** ) | | | RET% | | |
| --- | --- | --- | --- | --- | --- | --- | --- | --- | --- |
|  | D0 | D14 | D28 | D0 | D14 | D28 | D0 | D14 | D28 |
| 0 | -0.02±0.50 | 0.01±0.15 | 0.31±0.07 | 0.52±1.22 | 0.70±0.86 | 0.78±0.89 | 8.97±2.05 | 4.05±0.72 | 2.45±0.45 |
| 0.25 | -0.25±0.60 | 0.04±0.13 | 0.50±0.10 | 0.07±1.17 | 1.03±0.09 | 1.20±0.10 | 7.36±0.80 | 3.65±0.28 | 2.31±0.10 |
| 0.5 | -0.05±0.49 | 0.20±0.08 | 0.90±0.19 | 0.45±1.13 | 1.19±0.06 | 1.48±0.06 | 8.33±1.46 | 3.57±0.19 | 2.43±0.22 |
| 1 | -0.25±0.58 | 0.38±0.10 * | 1.08±0.32 | 0.43±1.11 | 1.41±0.11 * | 1.76±0.05 * | 8.45±1.17 | 3.24±0.73* | 2.55±0.29 |
| 2 | -0.11±0.44 | 0.49±0.10 * | 1.36±0.08 * | 0.54±1.20 | 1.46±0.08 * | 2.02±0.08 * | 7.39±0.59 | 2.81±0.18* | 2.18±0.19 |
| 4 | -0.20±0.64 | 0.64±0.09 * | 1.54±0.96 * | 0.92±0.95 | 1.81±0.08 * | 2.32±0.03 * | 7.12±0.33 | 3.22±0.50* | 2.32±0.37 |
| 8 | 0.07±0.54 | 0.94±0.05 * | 1.83±0.10 * | 0.53±1.19 | 1.93±0.05 * | 2.65±0.07 * | 7.39±1.10 | 3.07±0.21* | 2.12±0.29 |
| *P* value for Trend | 0.503 | 0.0001^#^ | 0.0001^#^ | 0.513 | 0.0001^#^ | 0.0001^#^ | 0.058 | 0.005^#^ | 0.041^#^ |

Abbreviation: ENU, N-ethyl-N-nitrosourea.

^a^  Mean ± S.D., n = 5.

* Statistically different from those in the vehicle control group (*P* < 0.05).

^#^ *P* < 0.05 in trend analysis.

Table 6 *Pig-a* gene mutation assay with EMS. ^a^

| **Doses (mg/kg.bw/d)** | RBC**^CD59-^** ( log_10_, ×10**^-6^** ) | | | RET**^CD59-^** ( log_10_, ×10**^-6^** ) | | | RET% | | |
| --- | --- | --- | --- | --- | --- | --- | --- | --- | --- |
|  | D0 | D14 | D28 | D0 | D14 | D28 | D0 | D14 | D28 |
| 0 | -0.47±0.58 | -0.54±0.72 | 0.09±0.24 | 0.75±0.91 | 0.79±0.90 | 1.17±0.24 | 8.27±1.02 | 3.28±0.82 | 3.06±0.20 |
| 5 | -0.45±0.62 | -0.67±0.51 | 0.18±0.19 | 0.44±1.13 | 0.53±1.21 | 0.37±0.88 | 7.98±0.80 | 3.55±0.52 | 2.74±0.49 |
| 10 | -0.60±0.61 | -0.21±0.44 | 0.15±0.37 | 0.29±1.03 | 0.76±0.92 | 1.22±0.18 | 8.80±0.54 | 3.42±0.34 | 2.93±0.35 |
| 20 | -0.44±0.62 | -0.24±0.62 | 0.36±0.18 | 0.46±1.14 | 0.40±1.11 | 1.22±0.22 | 8.57±0.75 | 3.0.2±0.35 | 2.82±0.32 |
| 40 | -0.60±0.63 | -0.28±0.45 | 0.55±0.08 * | 0.44±1.14 | 0.75±0.90 | 1.37±0.37 | 7.67±0.74 | 3.41±0.41 | 2.42±0.31 |
| 80 | -0.18±0.63 | 0.08±0.18 | 0.86±0.15 * | 0.50±1.16 | 1.34±0.23 | 1.41±0.31 | 7.89±1.15 | 2.83±0.37 | 3.13±0.65 |
| 160 | 0.54±0.72 | 0.15±0.20 * | 1.70±0.07 * | 0.74±0.08 | 1.77±0.08 | 2.00±0.02 * | 8.22±0.94 | 1.86±0.77 * | 5.09±1.22 * |
| *P* value for Trend | 0.8588 | 0.0046^#^ | 0.0001^#^ | 0.6373 | 0.0037^#^ | 0.0001^#^ | 0.5427 | 0.0001^#^ | 0.0001^#^ |

Abbreviation: EMS, Ethyl Methanesulfonate.

^A^  Mean ± S.D., n = 5.

* Statistically different from those in the vehicle control group (*P* < 0.05).

^#^ *P* < 0.05 in trend analysis.

Table 7 Peripheral blood micronucleus assay with ENU. ^a^

| **Doses (mg/kg.bw/d)** | **MN-RET ( log_10_ ×10^-3^ )** | | | | **RET%** | | | |
| --- | --- | --- | --- | --- | --- | --- | --- | --- |
|  | D0 | D4 | D14 | D28 | D0 | D4 | D14 | D28 |
| 0 | -0.75±0.22 | -0.65±0.12 | -0.54±0.13 | -0.60±0.11 | 2.60±0.39 | 2.32±0.48 | 1.73±0.26 | 0.84±0.15 |
| 0.25 | -0.78±0.24 | -0.47±0.18 | -0.52±0.18 | -0.57±0.19 | 2.69±0.62 | 2.94±0.43 | 1.51±0.26 | 1.05±0.30 |
| 0.5 | -0.70±0.23 | -0.57±0.20 | -0.40±0.07 | -0.44±0.13 | 2.68±0.45 | 2.92±0.57 | 1.58±0.35 | 0.85±0.11 |
| 1 | -0.80±0.22 | -0.53±0.16 | -0.41±0.16 | -0.42±0.19 | 2.78±0.67 | 2.80±0.53 | 1.69±0.21 | 0.91±0.23 |
| 2 | -0.71±0.25 | -0.49±0.33 | -0.31±0.07 * | -0.33±0.07 * | 2.49±0.50 | 2.32±0.80 | 1.09±0.37 * | 0.67±0.17 |
| 4 | -0.62±0.25 | -0.38±0.09 | -0.27±0.09 * | -0.23±0.11 * | 2..71±0.61 | 2.42±0.42 | 1.25±0.34 * | 0.51±0.09 * |
| 8 | -0.82±0.14 | -0.29±0.06 * | -0.24±0.20 * | -0.15±0.12 * | 2.73±0.58 | 1.77±0.59 | 0.97±0.33 * | 0.40±0.13 * |
| *P* value for Trend | 0.821 | 0.002^#^ | 0.0001^#^ | 0.0001^#^ | 0.835 | 0.001^#^ | 0.0001^#^ | 0.0001^#^ |

Abbreviation: ENU, N-ethyl-N-nitrosourea.

^a^  Mean ± S.D., n = 5.

* Statistically different from those in the vehicle control group (*P* < 0.05).

^#^ *P* < 0.05 in trend analysis.

Table 8 Bone marrow micronucleus assay with ENU. ^a^

| **Doses (mg/kg.bw/d)** | **MN-RET ( log_10_ ×10^-3^ )** | **RET%** |
| --- | --- | --- |
| 0 | 0.21±0.07 | 17.89±2.58 |
| 0.25 | 0.25±0.07 | 16.52±2.45 |
| 0.5 | 0.31±0.10 | 15.63±2.63 |
| 1 | 0.34±0.05 * | 14.96±1.70 |
| 2 | 0.58±0.05 * | 13.55±2.27 * |
| 4 | 0.62±0.15 * | 13.14±2.07 * |
| 8 | 0.82±0.06 * | 13.78±0.97 * |
| *P* value for Trend | 0.0001^#^ | 0.004^#^ |

Abbreviation: ENU, N-ethyl-N-nitrosourea.

^a^  Mean ± S.D., n = 5.

* Statistically different from those in the vehicle control group (*P* < 0.05).

^#^ *P* < 0.05 in trend analysis.

Table 9 Peripheral blood micronucleus assay with EMS. ^a^

| **Doses (mg/kg.bw/d)** | **MN-RET ( log_10_ ×10^-3^ )** | | | | **RET%** | | | |
| --- | --- | --- | --- | --- | --- | --- | --- | --- |
|  | D0 | D4 | D14 | D28 | D0 | D4 | D14 | D28 |
| 0 | -0.55±0.23 | -0.53±0.28 | -0.39±0.29 | -0.46±0.28 | 1.83±0.44 | 1.82±0.40 | 0.68±0.18 | 0.51±0.14 |
| 5 | -0.55±0.22 | -0.53±0.20 | -0.49±0.23 | -0.65±0.19 | 1.88±0.42 | 1.58±0.40 | 0.73±0.16 | 0.44±0.20 |
| 10 | -0.42±0.36 | -0.34±0.13 | -0.45±0.18 | -0.44±0.32 | 1.85±0.54 | 1.54±0.34 | 0.65±0.13 | 0.46±0.18 |
| 20 | -0.37±0.24 | -0.30±0.06 | -0.46±0.30 | -0.55±0.29 | 2.19±0.48 | 1.24±0.20 | 0.67±0.21 | 0.42±0.12 |
| 40 | -0.26±0.31 | -0.49±0.34 | -0.44±0.27 | -0.48±0.34 | 1.89±0.55 | 1.25±0.38 * | 0.76±0.17 | 0.41±0.13 |
| 80 | -0.43±0.19 | -0.42±0.25 | -0.49±0.26 | -0.10±0.12 * | 2..12±0.64 | 1.26±0.25 * | 0.64±0.18 | 0.79±0.45 |
| 160 | -0.43±0.33 | -0.09±0.20 * | 0.19±0.09 * | 0.16±0.09 * | 2.19±0.41 | 0.55±0.18 * | 0.41±0.17 * | 1.20±0.30 * |
| *P* value for Trend | 0.6433 | 0.0045^#^ | 0.0001^#^ | 0.0001^#^ | 0.1608 | 0.0001^#^ | 0.0023^#^ | 0.0001^#^ |

Abbreviation: EMS, Ethyl Methanesulfonate.

^a^  Mean ± S.D., n = 5.

* Statistically different from those in the vehicle control group (*P* < 0.05).

^#^ *P* < 0.05 in trend analysis.

Table 10 Bone marrow micronucleus assay with EMS. ^a^

| **Doses (mg/kg.bw/d)** | **MN-RET ( log_10_ ×10^-3^ )** | **RET%** |
| --- | --- | --- |
| 0 | -0.32±0.18 | 17.05±0.80 |
| 5 | -0.32±0.28 | 17.34±0.64 |
| 10 | -0.24±0.12 | 16.99±0.82 |
| 20 | -0.28±0.16 | 16.66±0.80 |
| 40 | 0.03±0.09 * | 16.11±0.85 |
| 80 | 0.13±0.10 * | 17.20±0.94 |
| 160 | 0.18±0.07 * | 19.80±1.00 * |
| *P* value for Trend | 0.0001^#^ | 0.0001^#^ |

Abbreviation: EMS, Ethyl Methanesulfonate.

^a^  Mean ± S.D., n = 5.

* Statistically different from those in the vehicle control group (*P* < 0.05).

^#^ *P* < 0.05 in trend analysis.

Table 11 Comet assay with ENU. ^a^

| **Doses (mg/kg.bw/d)** | **PB Tail DNA( log_10_, % )** | | | **Liver Tail DNA( log_10_, % )** |
| --- | --- | --- | --- | --- |
|  | D4 | D14 | D28 | D29 |
| 0 | 0.69±0.03 | 0.79±0.05 | 0.79±0.04 | 0.89±0.02 |
| 0.25 | 0.71±0.06 | 0.83±0.12 | 0.79±0.05 | 0.89±0.03 |
| 0.5 | 0.75±0.03 | 0.90±0.04 * | 0.94±0.03 * | 0.96±0.06 * |
| 1 | 0.98±0.03 * | 0.98±0.04 * | 1.10±0.05 * | 1.17±0.03 * |
| 2 | 1.10±0.04 * | 1.12±0.05 * | 1.09±0.04 * | 1.22±0.01 * |
| 4 | 1.19±0.06 * | 1.17±0.04 * | 1.16±0.08 * | 1.29±0.01 * |
| 8 | 1.26±0.04 * | 1.24±0.05 * | 1.31±0.05 * | 1.31±0.03 * |
| *P* value for Trend | 0.0001^#^ | 0.0001^#^ | 0.0001^#^ | 0.0001^#^ |

Abbreviation: ENU, N-ethyl-N-nitrosourea. PB, Peripheral blood.

^a^  Mean ± S.D., n = 5.

* Statistically different from those in the vehicle control group (*P* < 0.05).

^#^ *P* < 0.05 in trend analysis.

Table 12 Comet assay with EMS. ^a^

| **Doses (mg/kg.bw/d)** | **PB Tail DNA( log_10_, % )** | | **Liver Tail DNA( log_10_, % )** |
| --- | --- | --- | --- |
|  | D4 | D28 | D29 |
| 0 | 0.64±0.10 | 0.71±0.09 | 0.70±0.08 |
| 5 | 0.68±0.07 | 0.66±0.17 | 0.73±0.07 |
| 10 | 0.74±0.06 | 0.65±0.08 | 0.79±0.09 |
| 20 | 0.76±0.09 | 0.74±0.11 | 0.82±0.05 |
| 40 | 1.10±1.14 * | 1.11±0.13 * | 1.08±1.15 * |
| 80 | 1.21±0.08 * | 1.18±0.10 * | 1.52±0.08 * |
| 160 | 1.50±0.07 * | 1.43±0.10 * | 1.71±0.07 * |
| *P* value for Trend | 0.0001^#^ | 0.0001^#^ | 0.0001^#^ |

Abbreviation: EMS, Ethyl Methanesulfonate. PB, Peripheral blood.

^a^  Mean ± S.D., n = 5.

* Statistically different from those in the vehicle control group (*P* < 0.05).

^#^ *P* < 0.05 in trend analysis.

**Supplementary material 2. The analysis results of the PROAST**

1. **The PoDs of genotoxic endpoints of ENU**
   1. ***Pig-a* gene mutation assay**
      1. **RBC at day 14 with CES0.05**


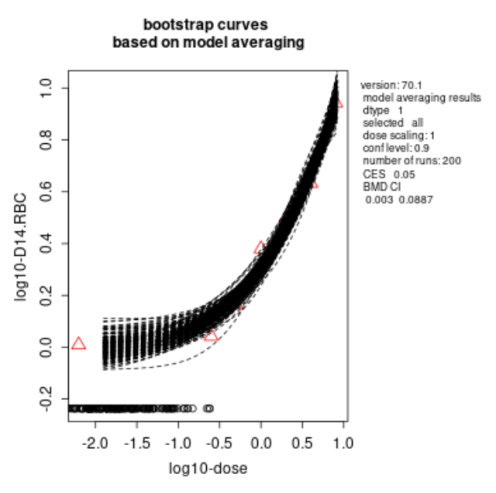

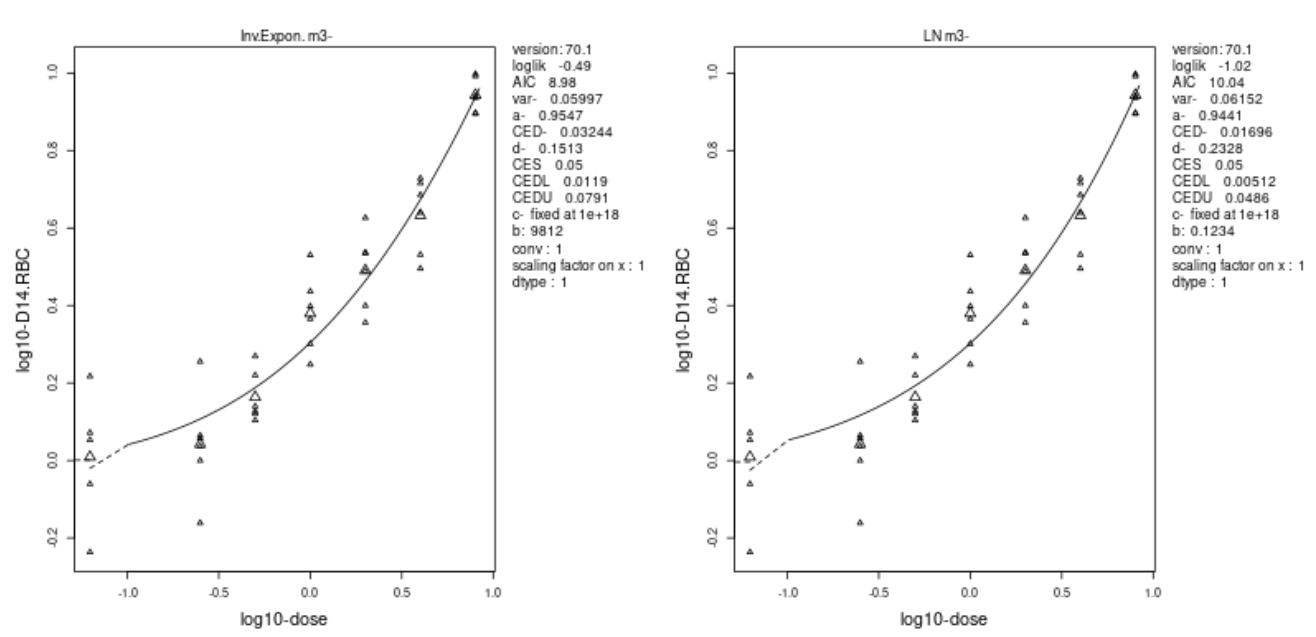


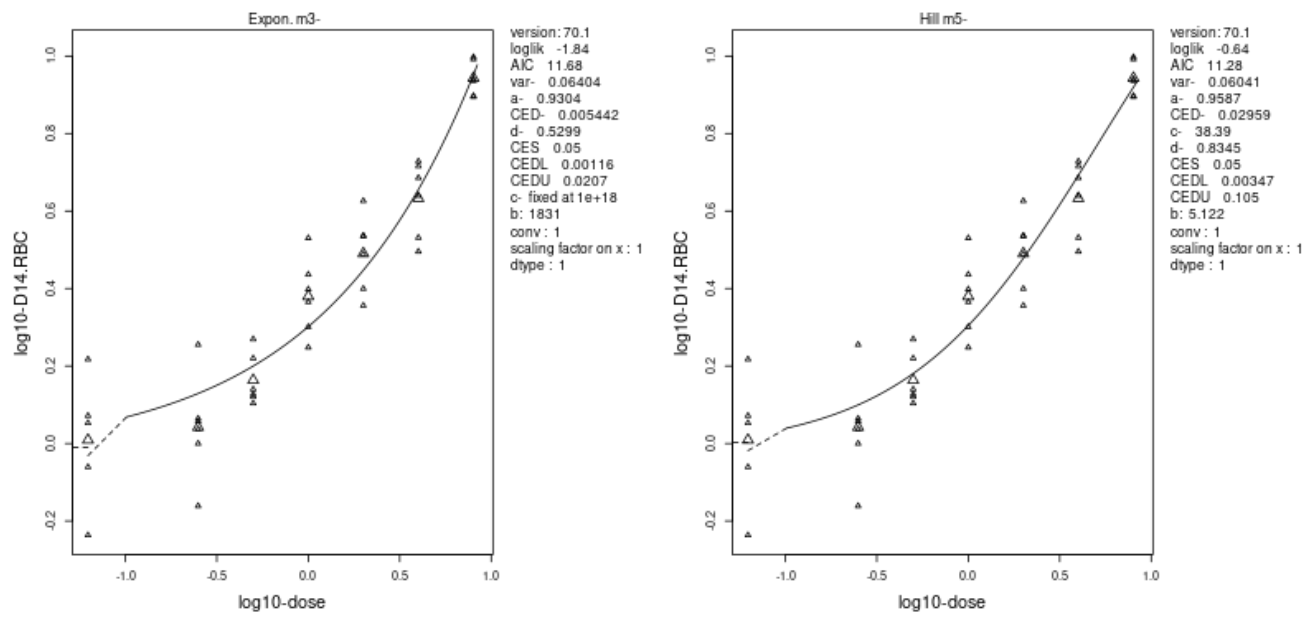


| CES | BMDL (mg/kg.bw) | BMDU (mg/kg.bw) |
| --- | --- | --- |
| 0.05 | 0.003 | 0.0887 |

- - 1. **RBC at day 14 with CES0.1**


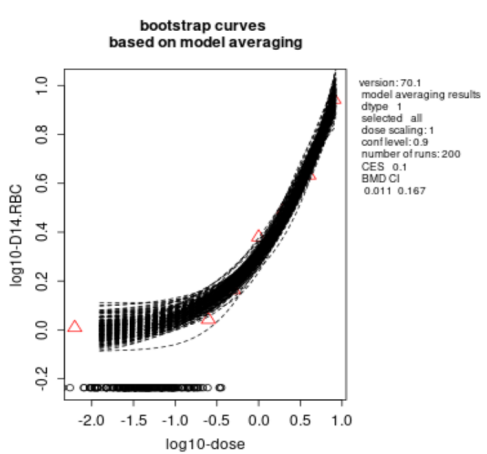

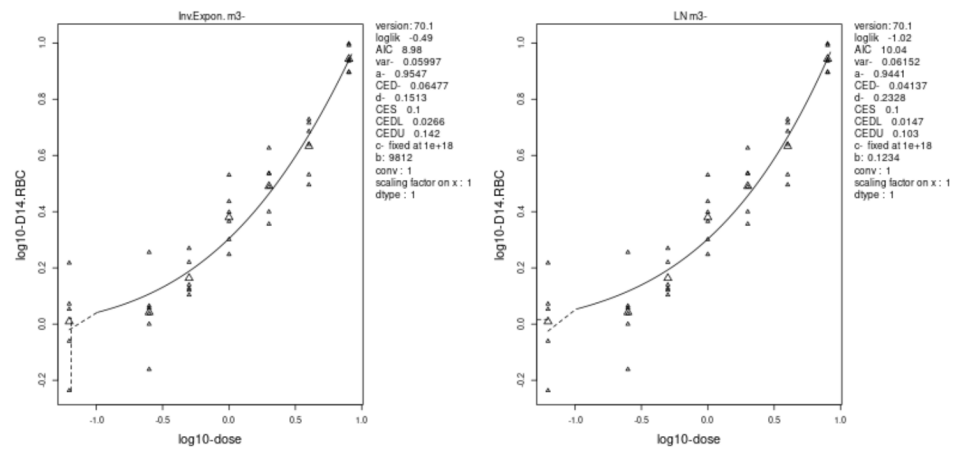


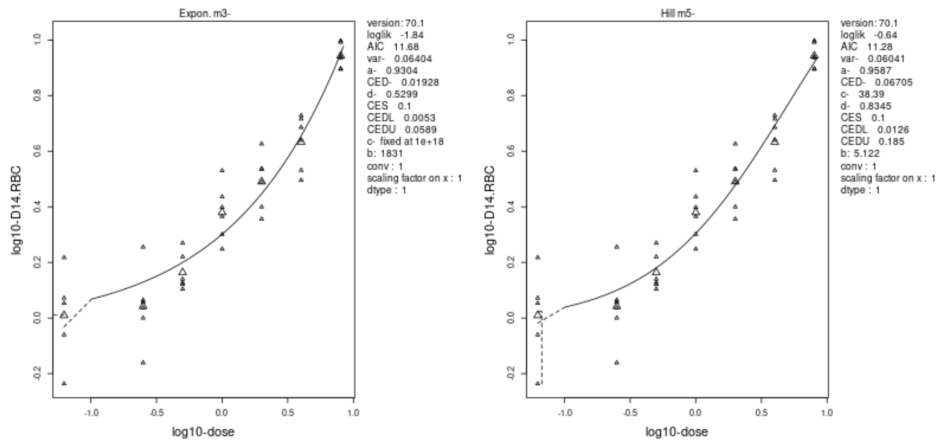


| CES | BMDL (mg/kg.bw) | BMDU (mg/kg.bw) |
| --- | --- | --- |
| 0.1 | 0.011 | 0.167 |

- - 1. **RBC at day 14 with CES0.5**


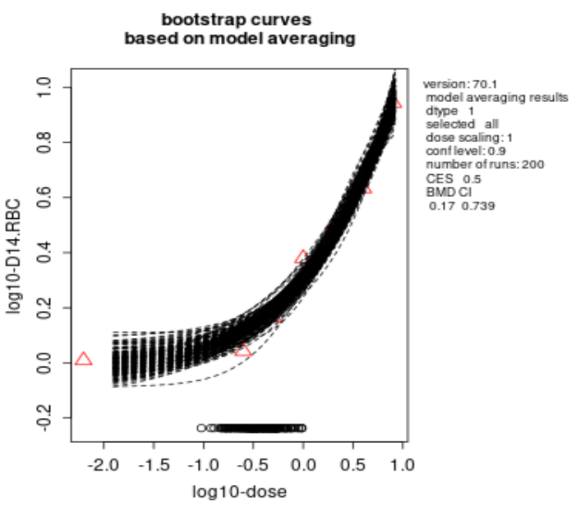

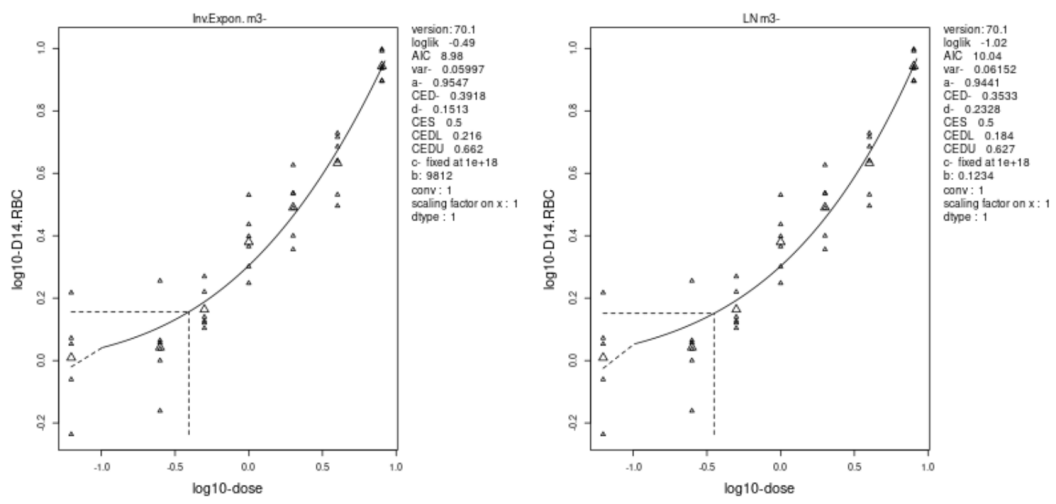


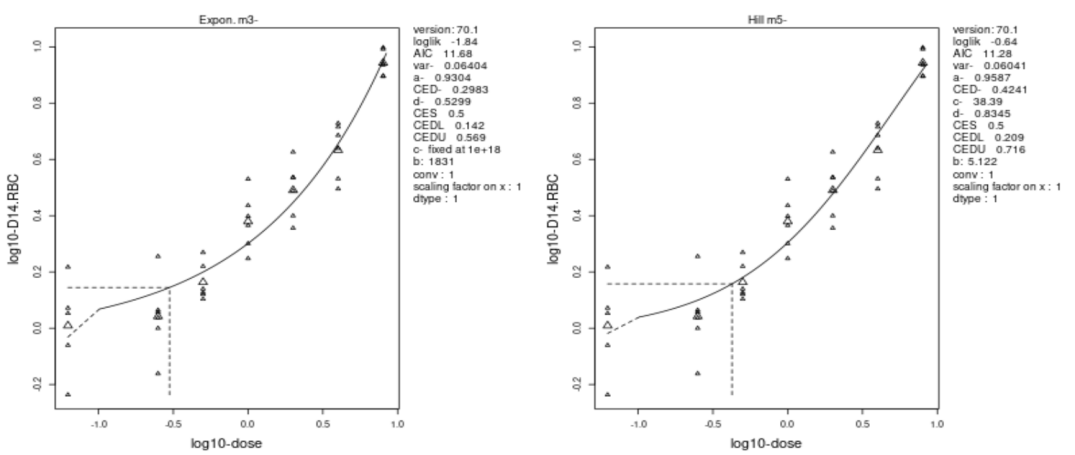


| CES | BMDL (mg/kg.bw) | BMDU (mg/kg.bw) |
| --- | --- | --- |
| 0.5 | 0.17 | 0.739 |

- - 1. **RBC at day 14 with CES1SD**


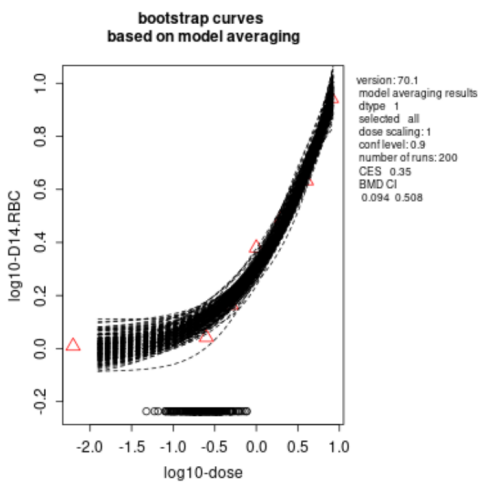

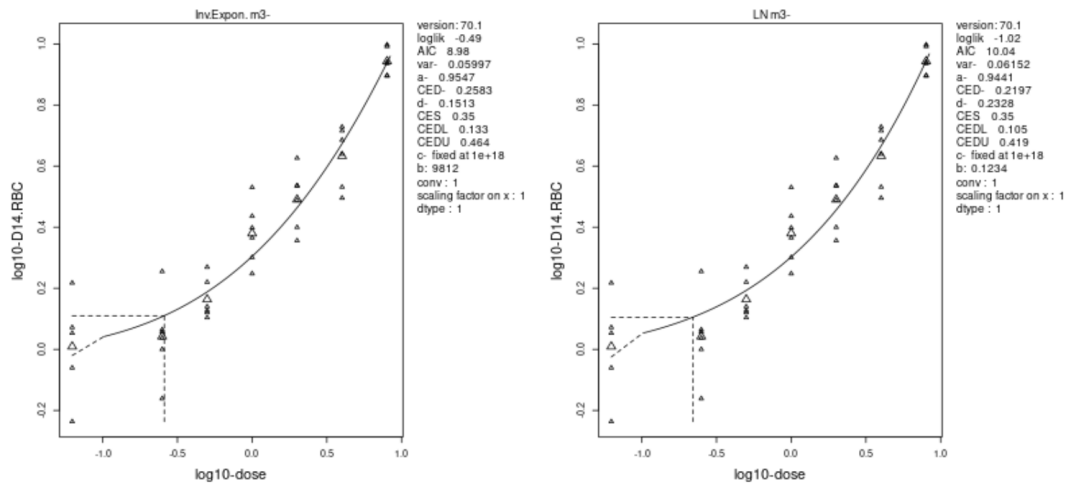


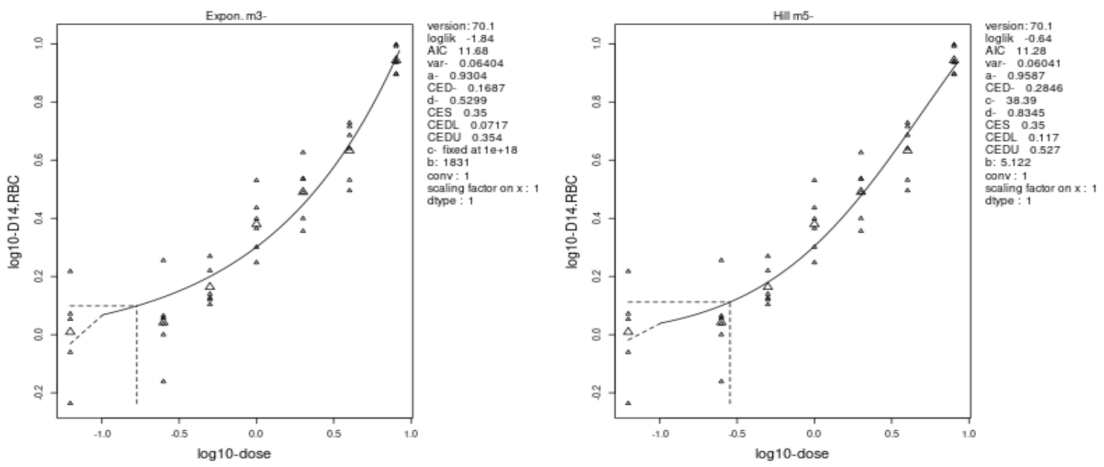


| CES | BMDL (mg/kg.bw) | BMDU (mg/kg.bw) |
| --- | --- | --- |
| 1SD | 0.094 | 0.508 |

- - 1. **RET at day 14 with CES0.05**


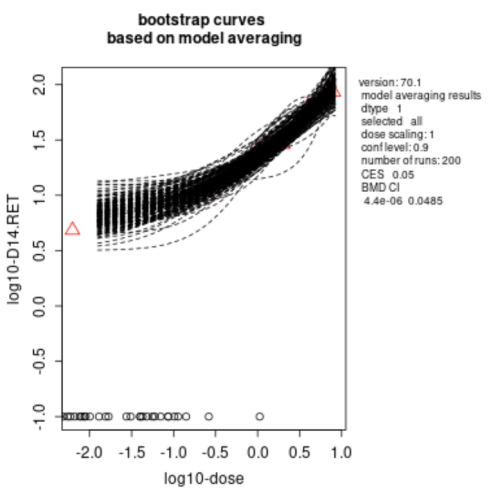

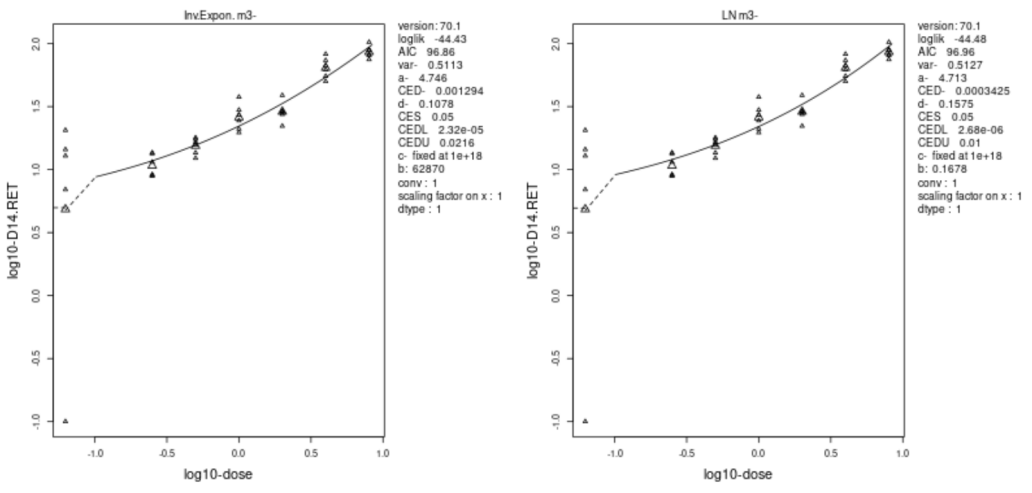


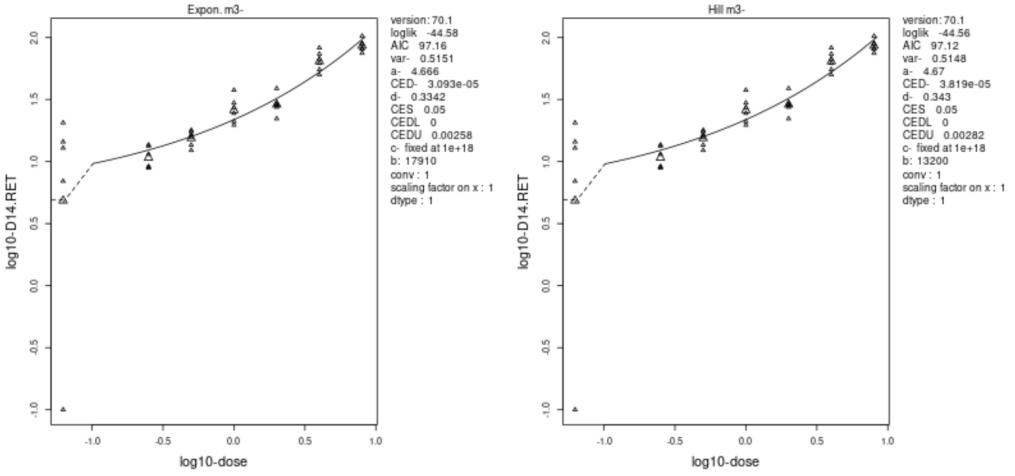


| CES | BMDL (mg/kg.bw) | BMDU (mg/kg.bw) |
| --- | --- | --- |
| 0.05 | 4.42e-06 | 0.0485 |

- - 1. **RET at day 14 with CES0.1**


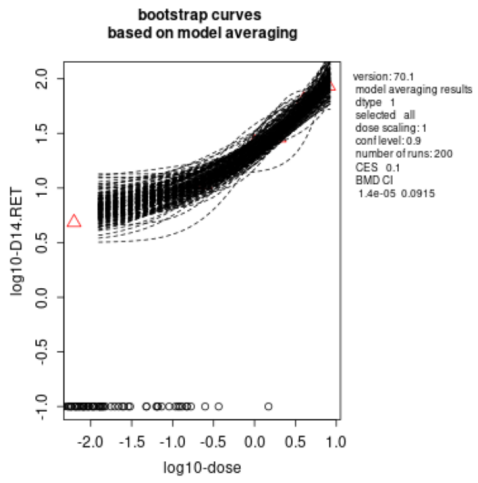

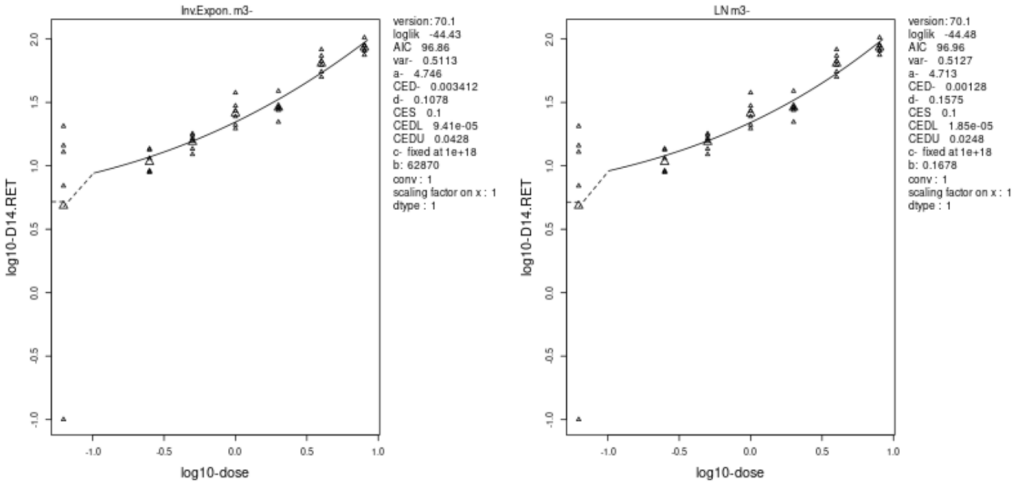


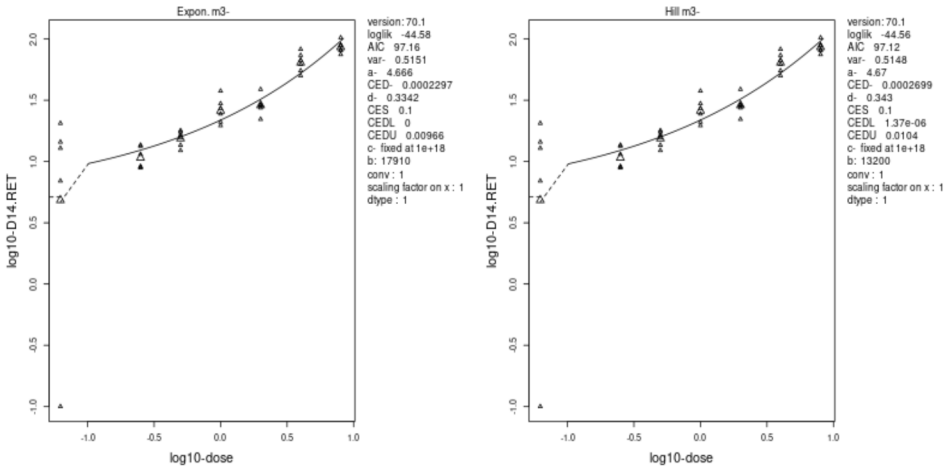


| CES | BMDL (mg/kg.bw) | BMDU (mg/kg.bw) |
| --- | --- | --- |
| 0.1 | 1.4e-05 | 0.0915 |

- - 1. **RET at day 14 with CES0.5**


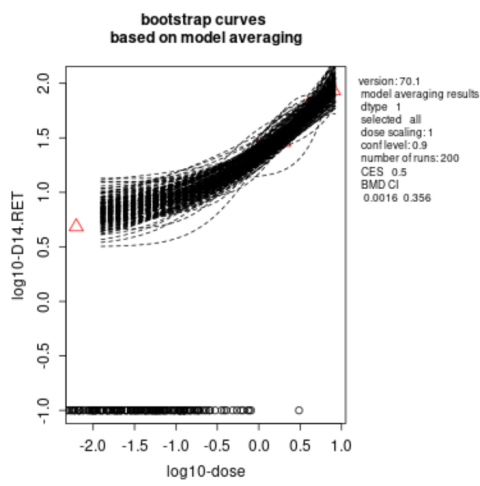

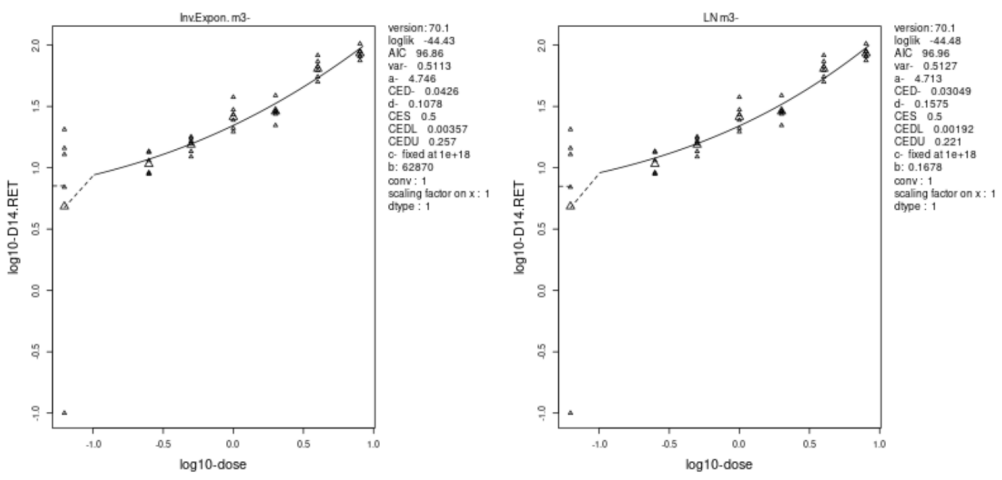


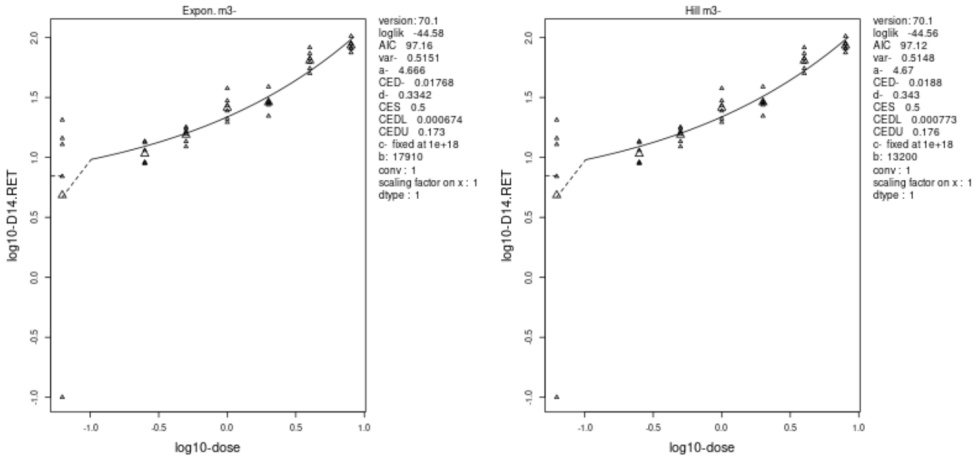


| CES | BMDL (mg/kg.bw) | BMDU (mg/kg.bw) |
| --- | --- | --- |
| 0.5 | 0.0016 | 0.356 |

- - 1. **RET at day 14 with CES1SD**


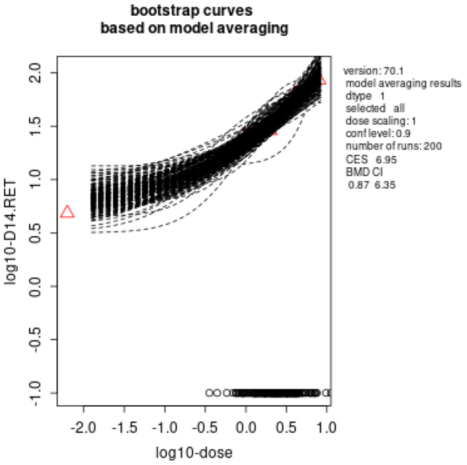

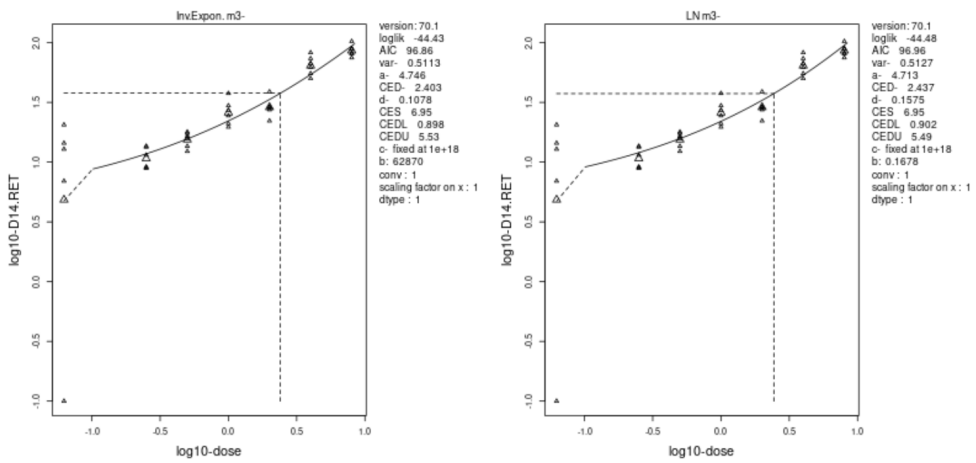


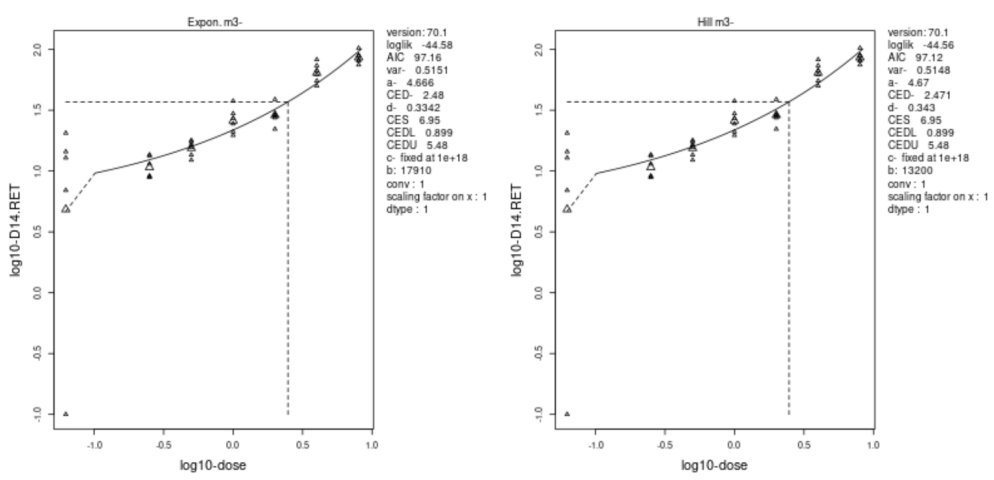


| CES | BMDL (mg/kg.bw) | BMDU (mg/kg.bw) |
| --- | --- | --- |
| 1SD | 0.87 | 6.35 |

- - 1. **RBC at day 28 with CES0.05**


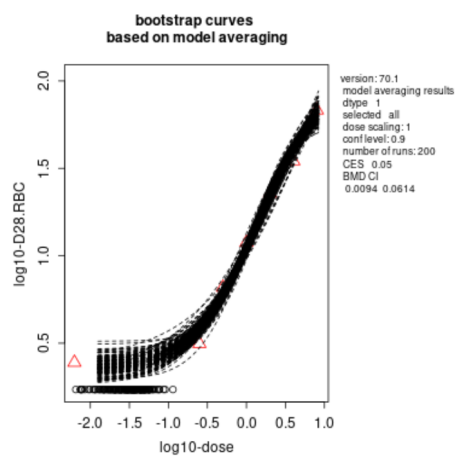

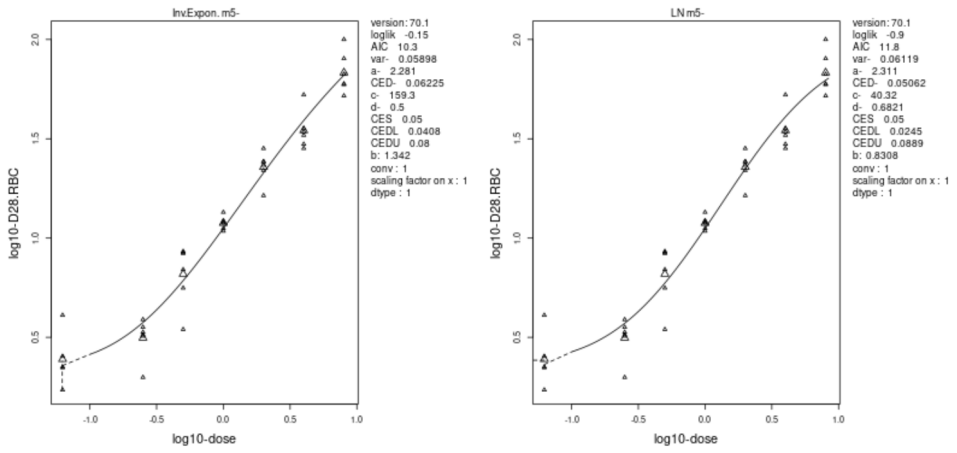


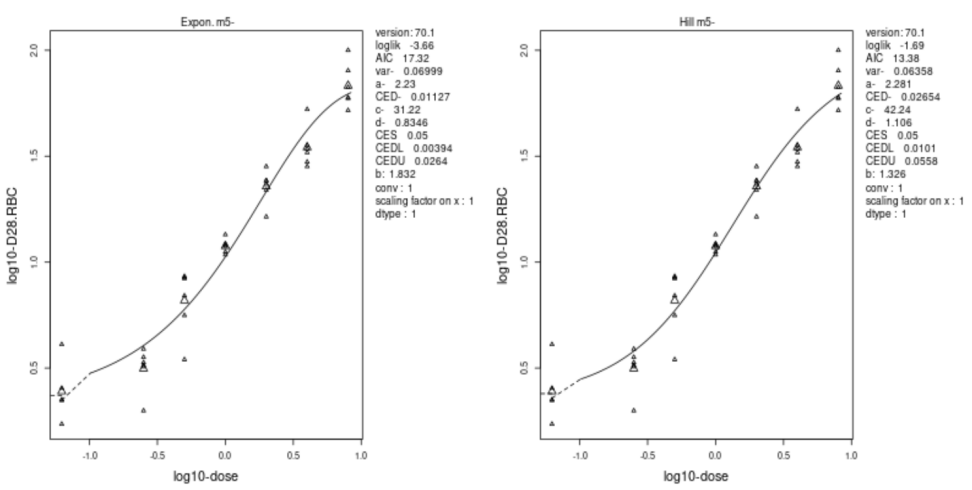


| CES | BMDL (mg/kg.bw) | BMDU (mg/kg.bw) |
| --- | --- | --- |
| 0.05 | 0.0094 | 0.0614 |

- - 1. **RBC at day 28 with CES0.1**


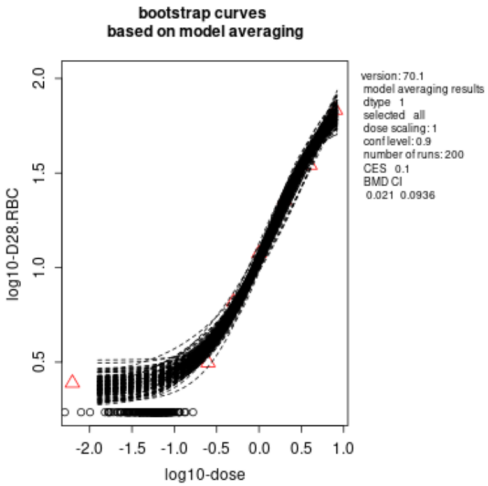

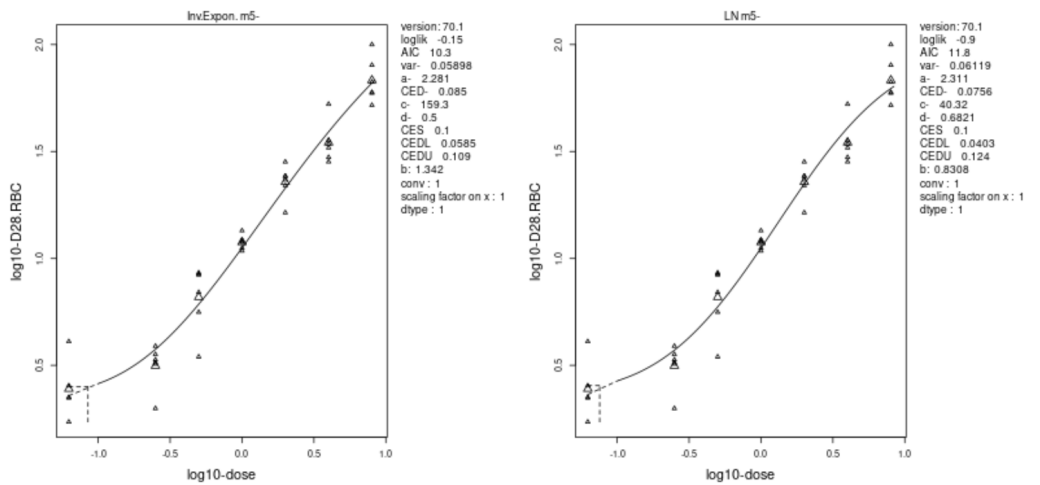


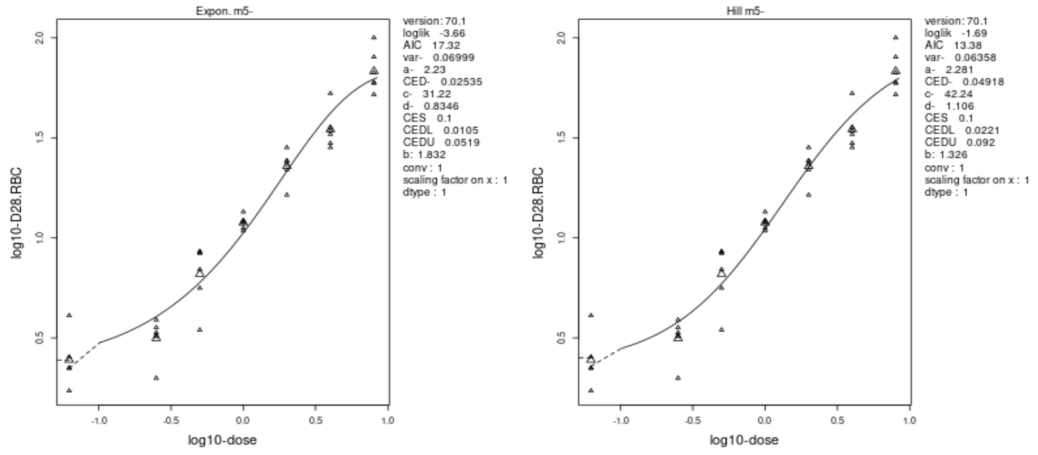


| CES | BMDL (mg/kg.bw) | BMDU (mg/kg.bw) |
| --- | --- | --- |
| 0.1 | 0.021 | 0.0936 |

- - 1. **RBC at day 28 with CES0.5**


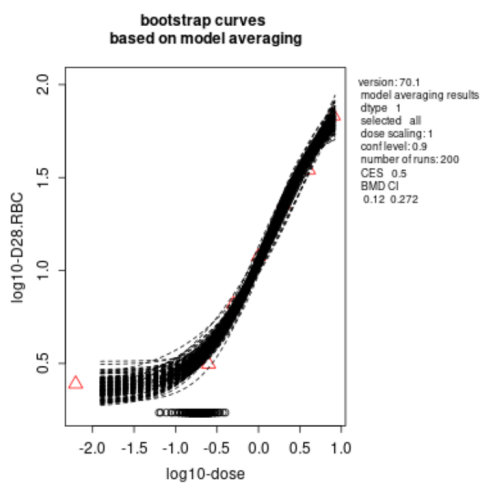

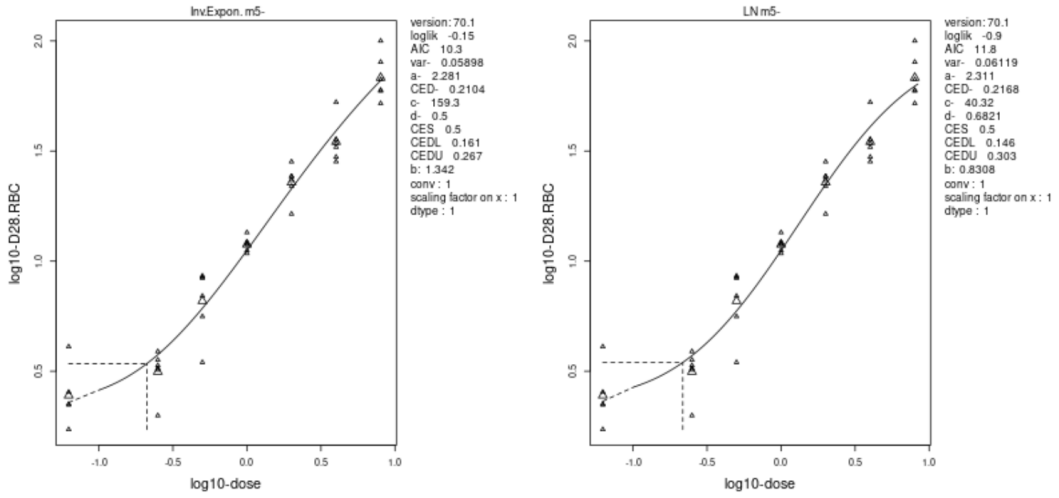


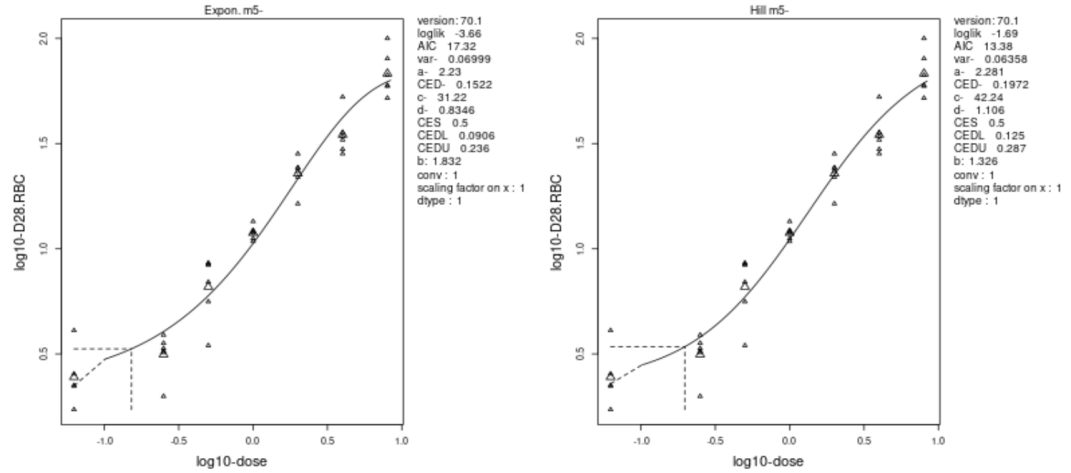


| CES | BMDL (mg/kg.bw) | BMDU (mg/kg.bw) |
| --- | --- | --- |
| 0.5 | 0.12 | 0.272 |

- - 1. **RBC at day 28 with CES1SD**


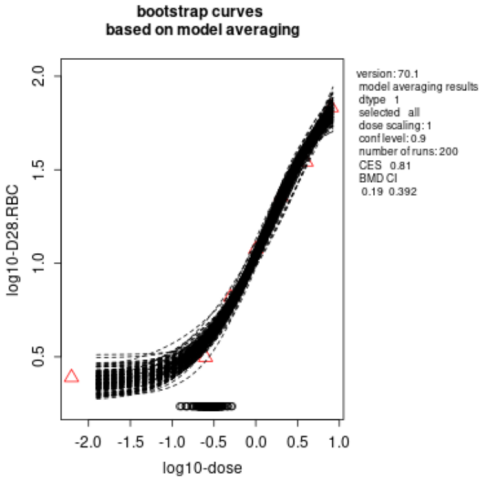

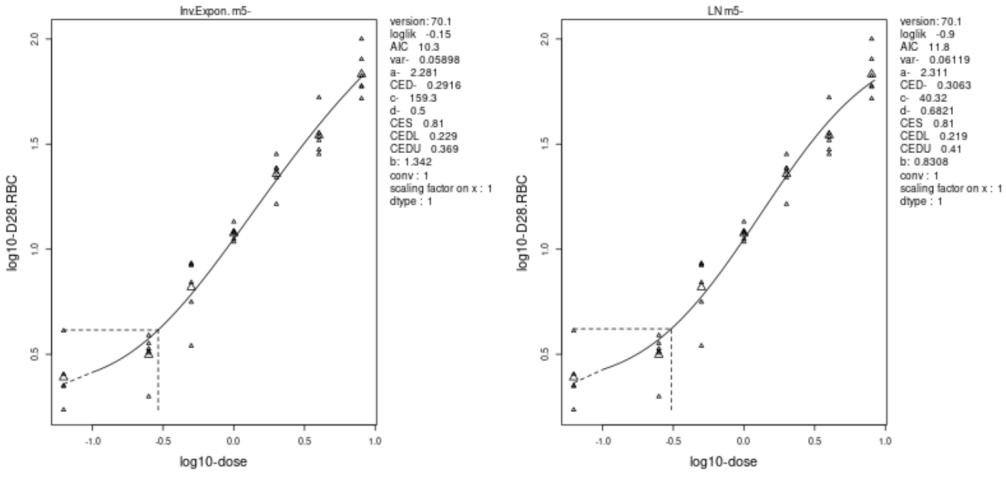


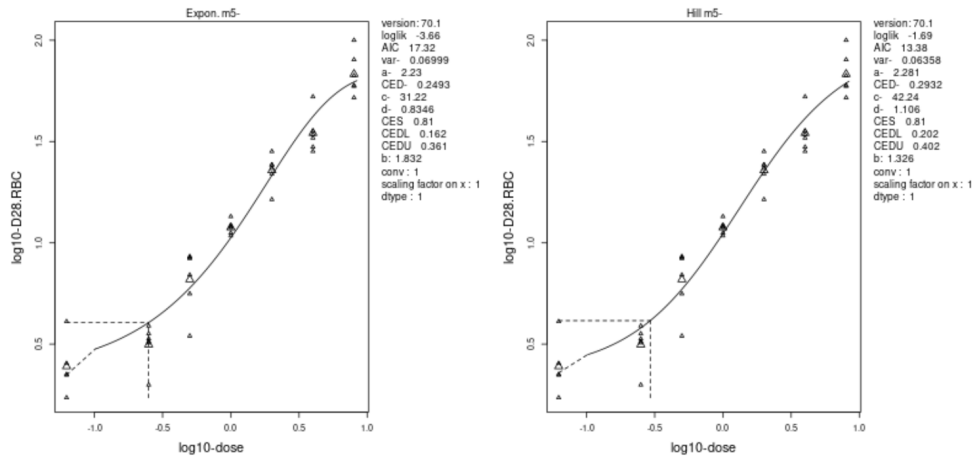


| CES | BMDL (mg/kg.bw) | BMDU (mg/kg.bw) |
| --- | --- | --- |
| 1SD | 0.19 | 0.392 |

- - 1. **RET at day 28 with CES0.05**


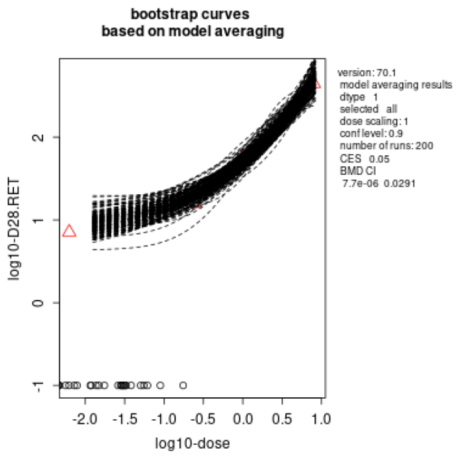

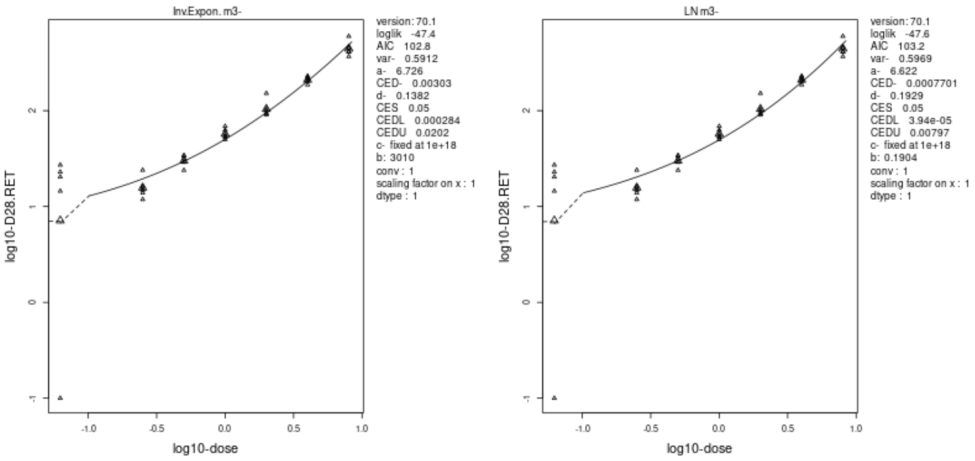


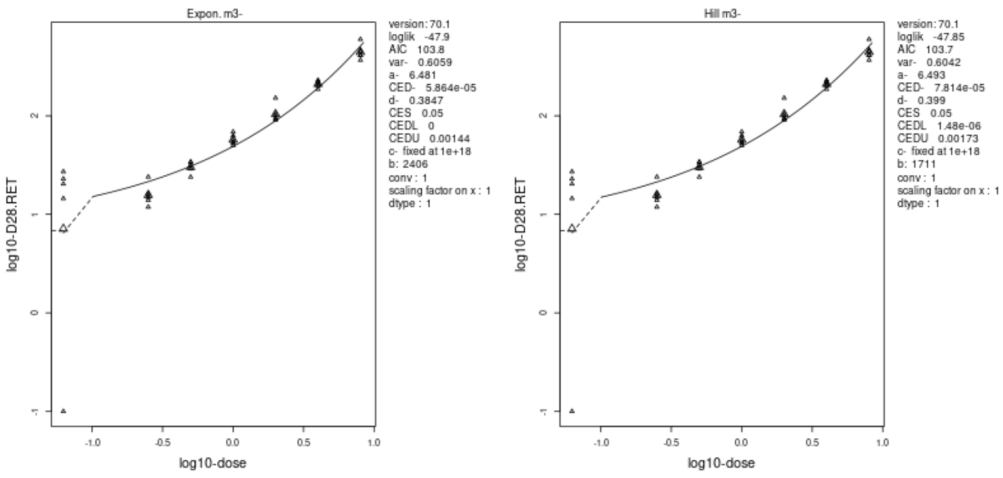


| CES | BMDL (mg/kg.bw) | BMDU (mg/kg.bw) |
| --- | --- | --- |
| 0.05 | 7.7e-06 | 0.0291 |

- - 1. **RET at day 28 with CES0.1**


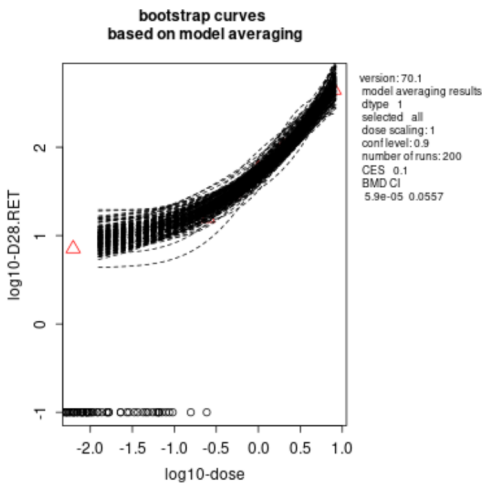

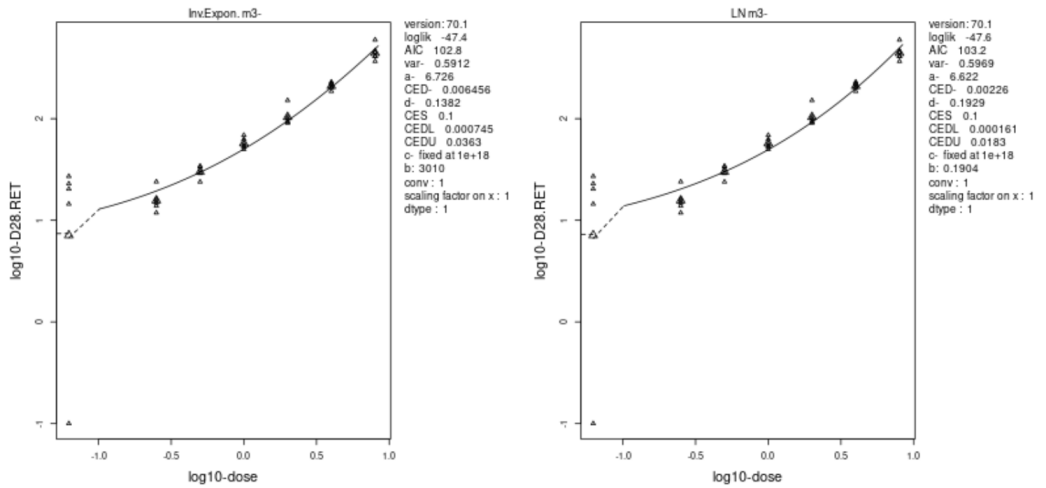


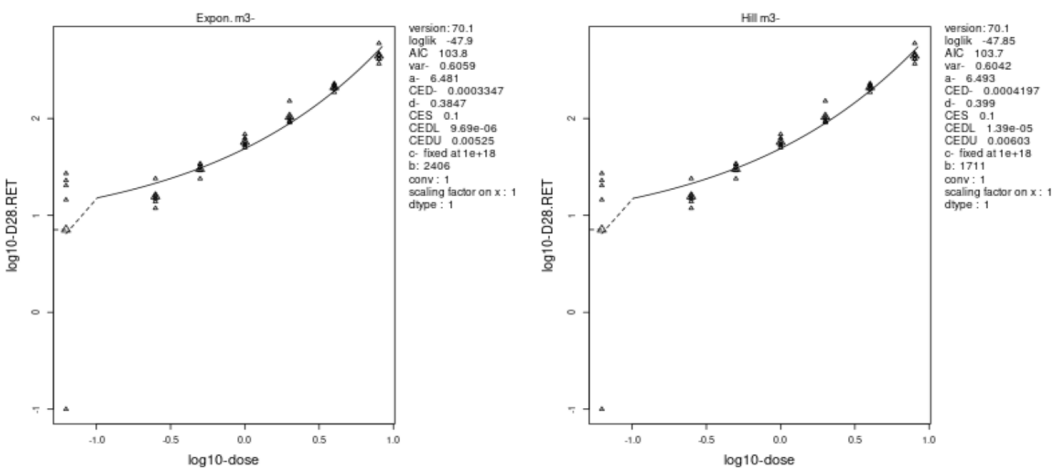


| CES | BMDL (mg/kg.bw) | BMDU (mg/kg.bw) |
| --- | --- | --- |
| 0.1 | 5.9e-05 | 0.0557 |

- - 1. **RET at day 28 with CES0.5**


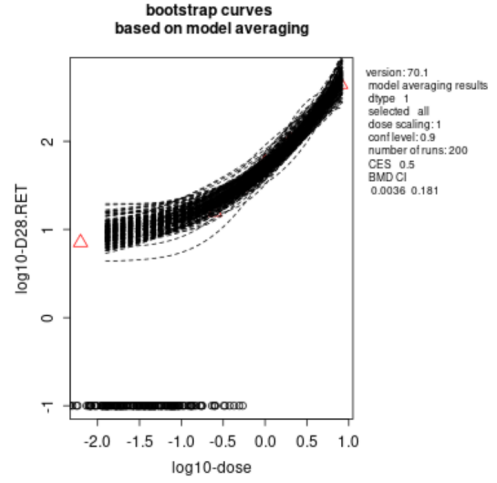

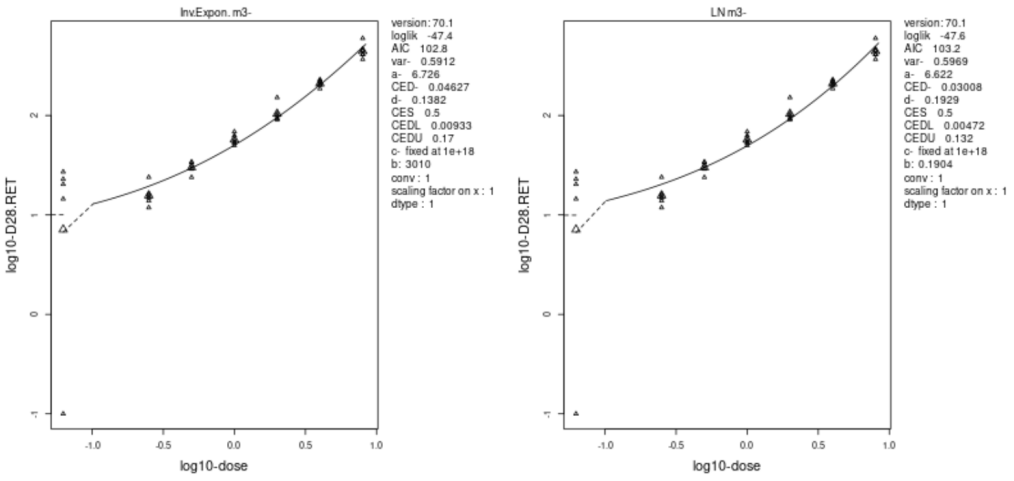


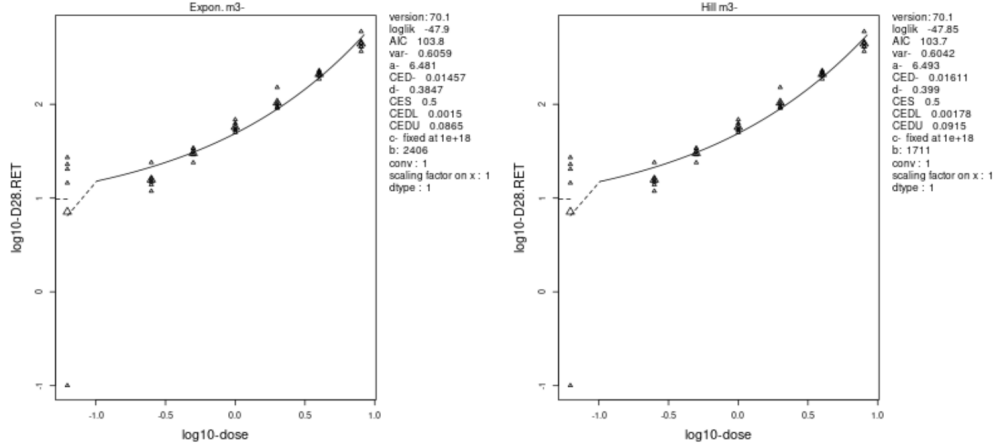


| CES | BMDL (mg/kg.bw) | BMDU (mg/kg.bw) |
| --- | --- | --- |
| 0.5 | 0.0036 | 0.181 |

- - 1. **RET at day 28 with CES1SD**


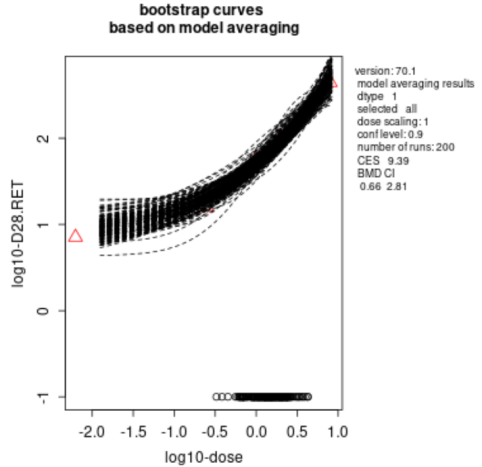

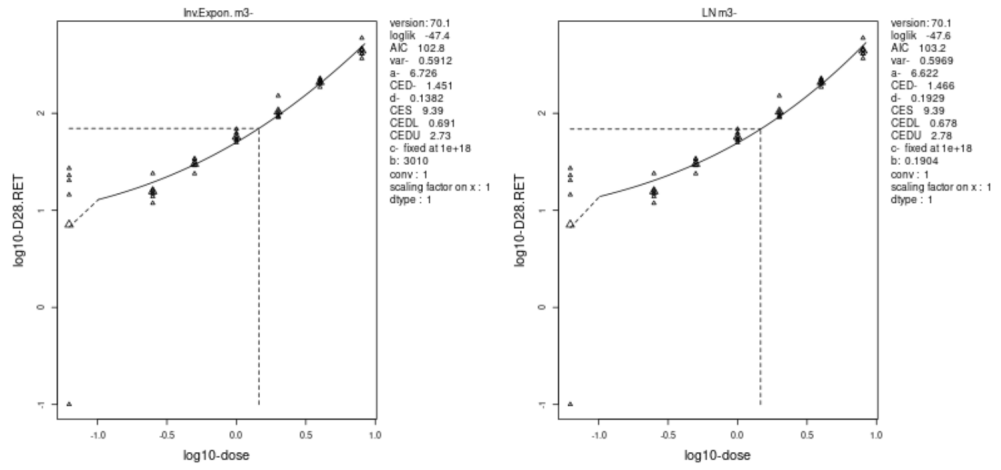


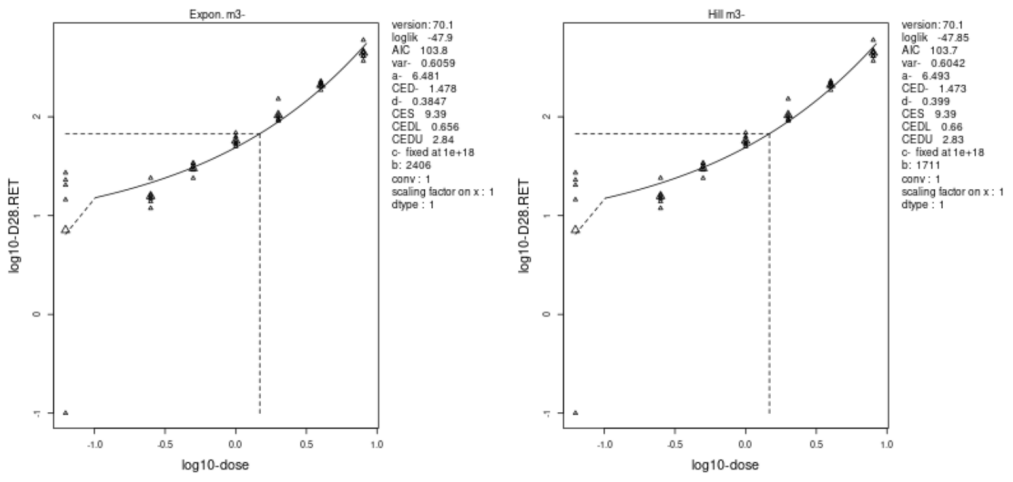


| CES | BMDL (mg/kg.bw) | BMDU (mg/kg.bw) |
| --- | --- | --- |
| 1SD | 0.66 | 2.81 |

- 1. **Micronucleus assay**
     1. **RET at day 4 with CES0.05**


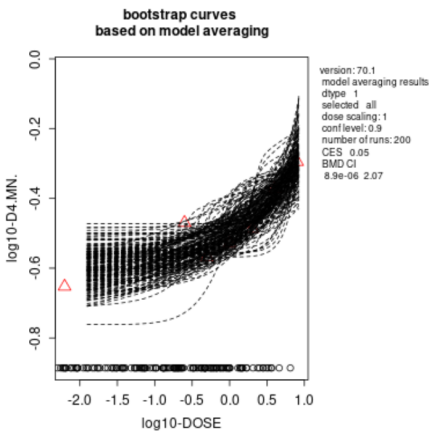

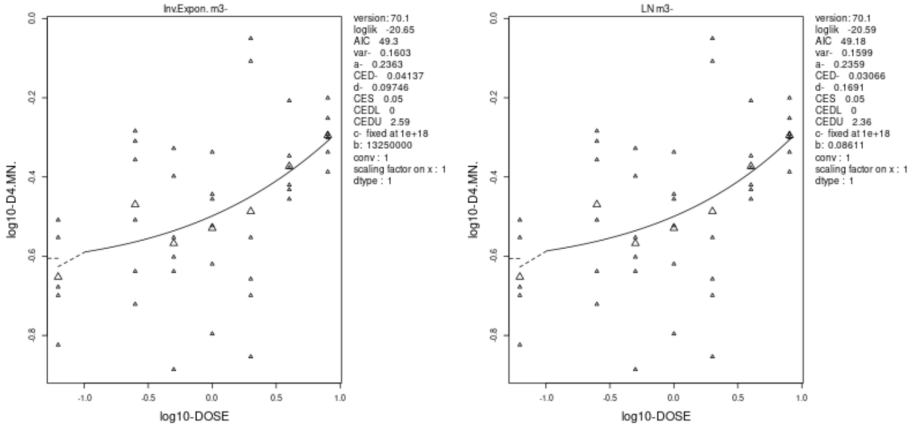


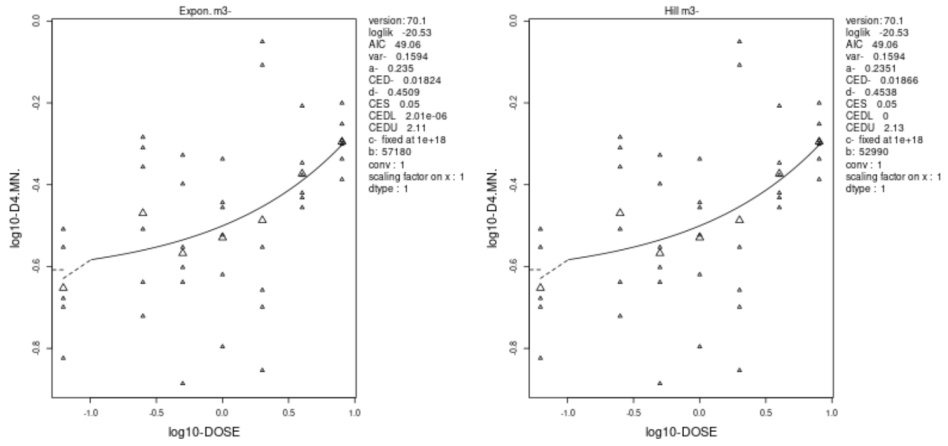


| CES | BMDL (mg/kg.bw) | BMDU (mg/kg.bw) |
| --- | --- | --- |
| 0.05 | 8.9e-06 | 2.07 |

- - 1. **RET at day 4 with CES0.1**


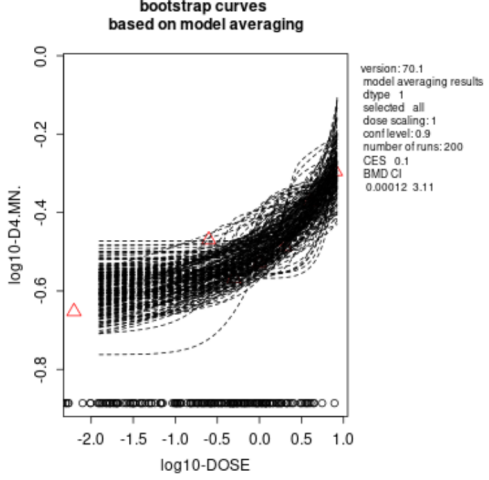

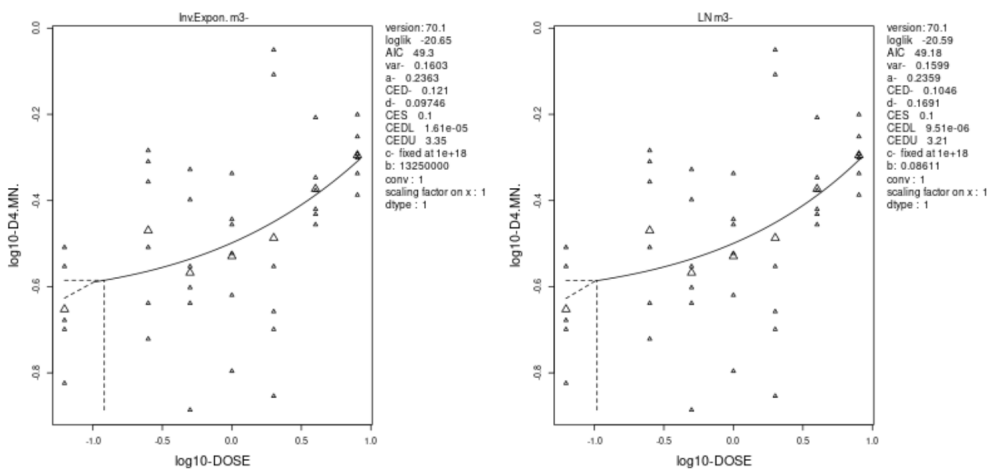


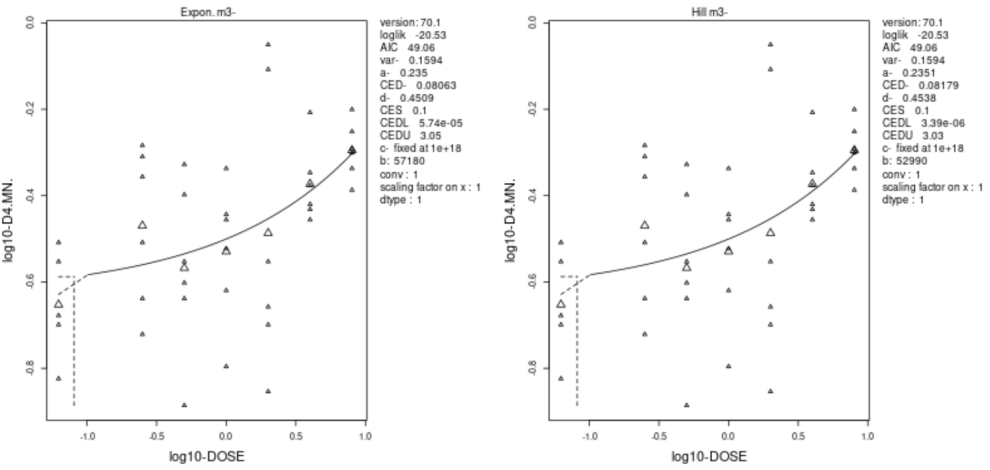


| CES | BMDL (mg/kg.bw) | BMDU (mg/kg.bw) |
| --- | --- | --- |
| 0.1 | 0.00012 | 3.11 |

- - 1. **RET at day 4 with CES0.5**


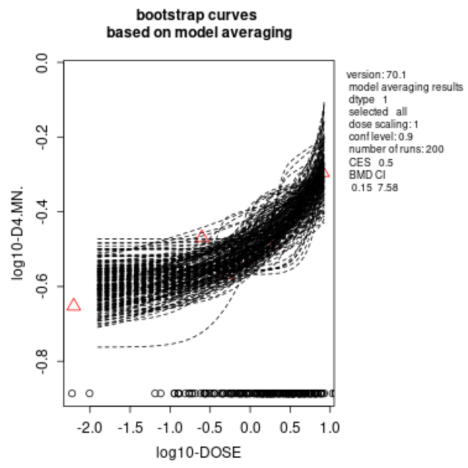

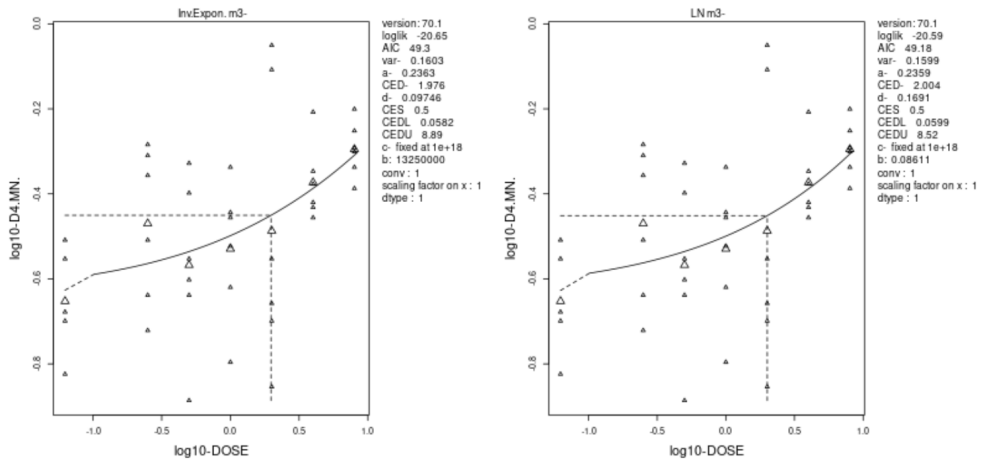


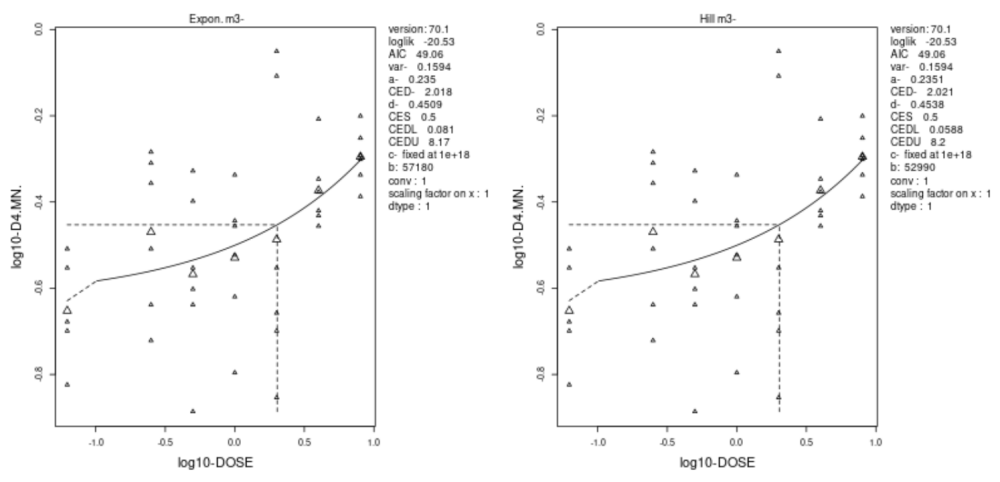


| CES | BMDL (mg/kg.bw) | BMDU (mg/kg.bw) |
| --- | --- | --- |
| 0.5 | 0.15 | 7.58 |

- - 1. **RET at day 4 with CES1SD**


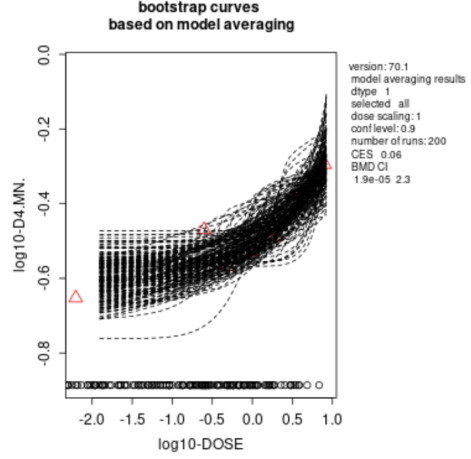

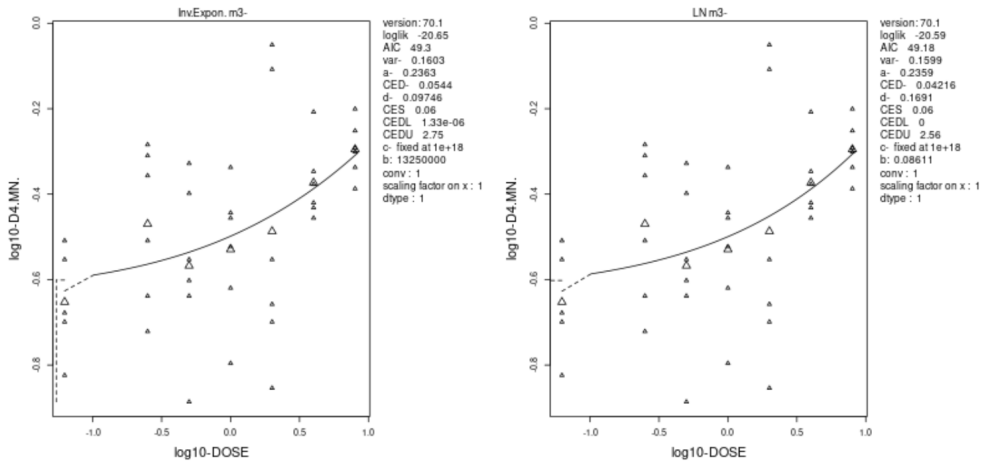


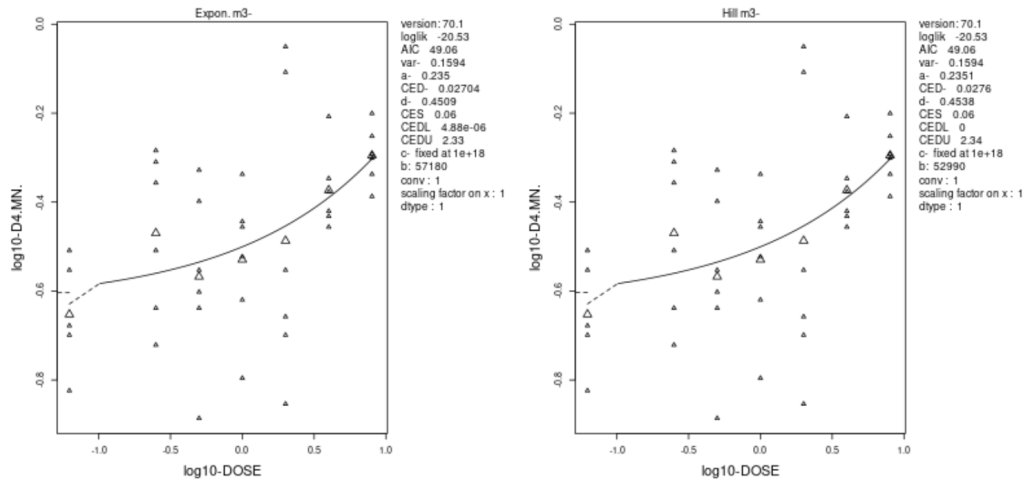


| CES | BMDL (mg/kg.bw) | BMDU (mg/kg.bw) |
| --- | --- | --- |
| 1SD | 1.9e-05 | 2.3 |

- - 1. **RET at day 14 with CES0.05**


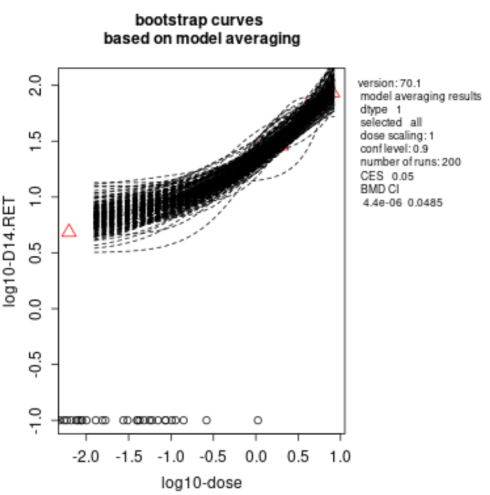

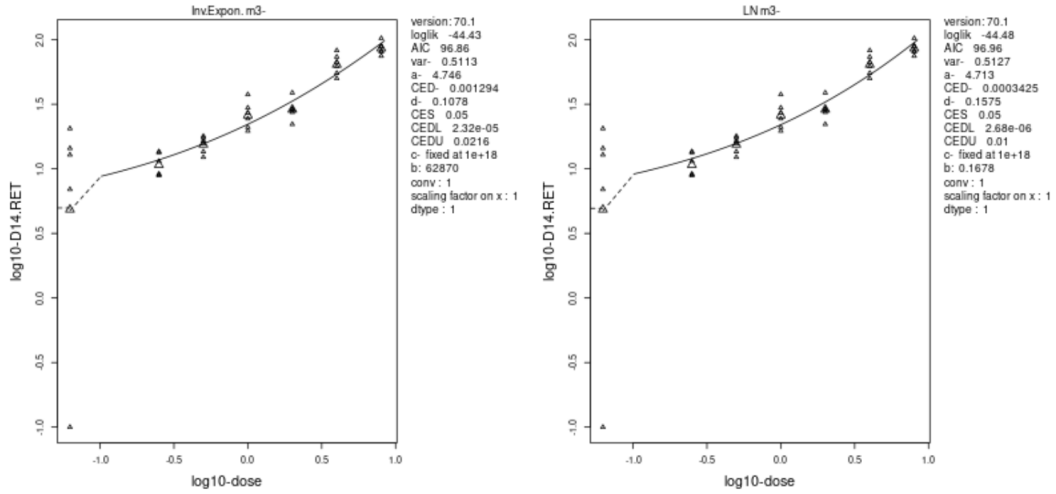


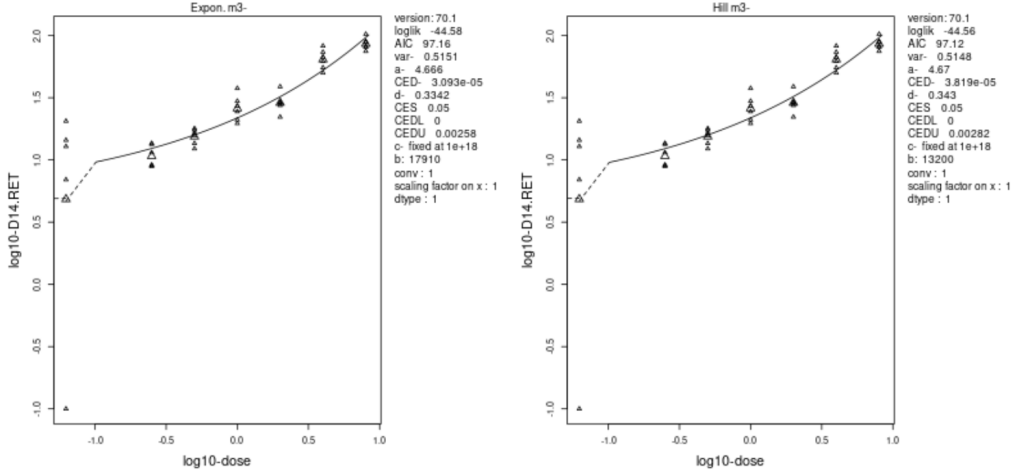


| CES | BMDL (mg/kg.bw) | BMDU (mg/kg.bw) |
| --- | --- | --- |
| 0.05 | 4.4e-06 | 0.0485 |

- - 1. **RET at day 14 with CES0.1**


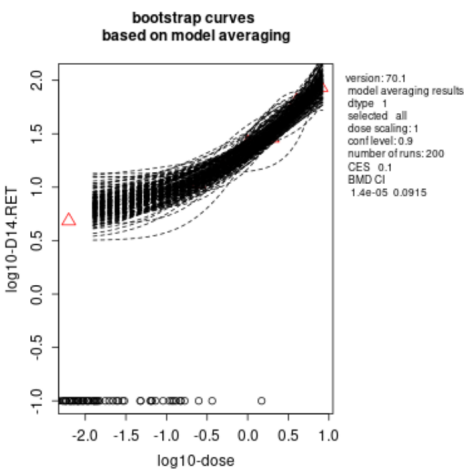

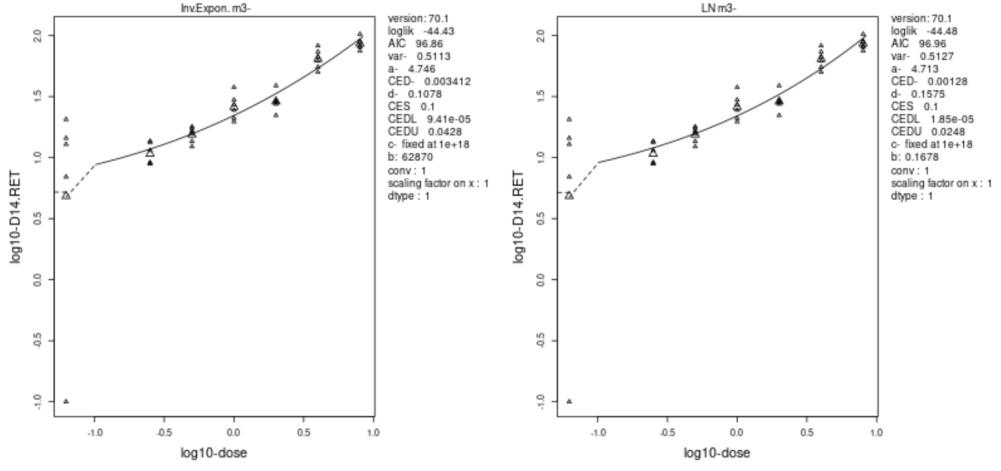


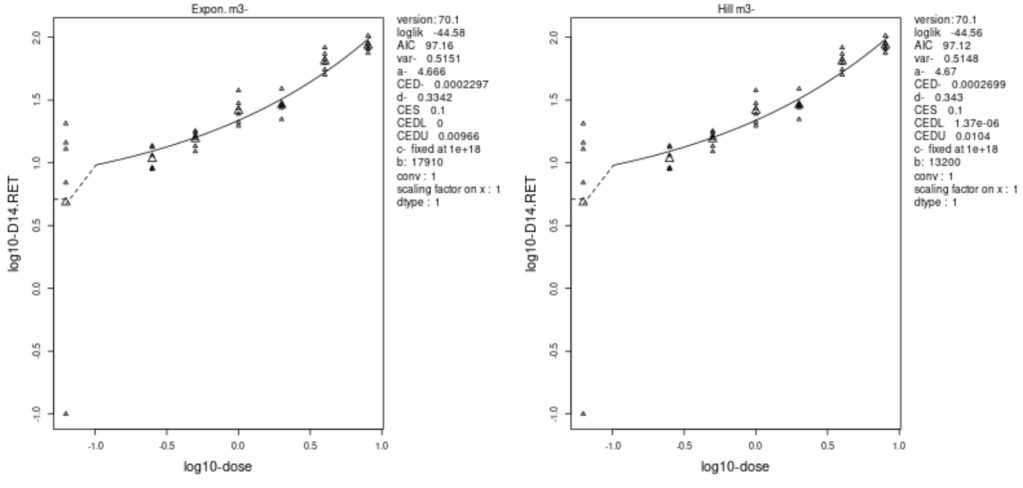


| CES | BMDL (mg/kg.bw) | BMDU (mg/kg.bw) |
| --- | --- | --- |
| 0.1 | 1.4e-05 | 0.0915 |

- - 1. **RET at day 14 with CES0.5**


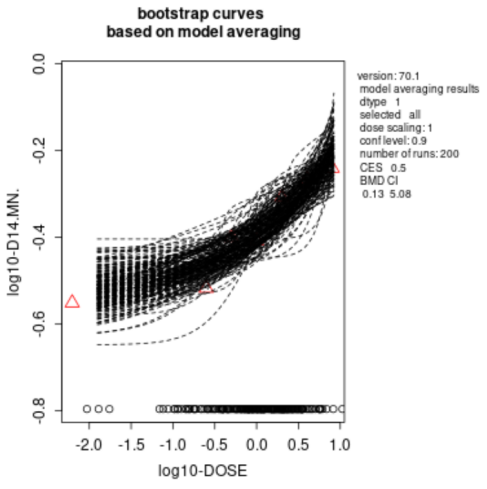

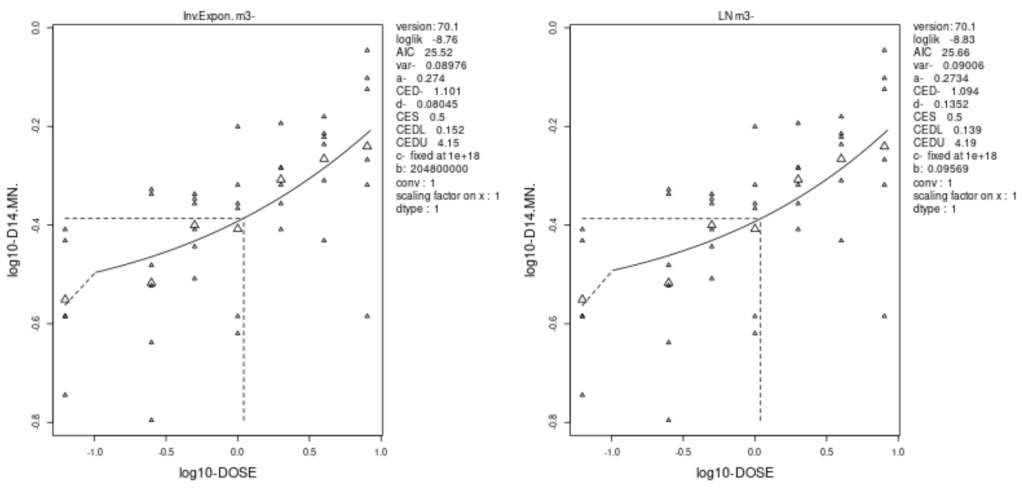


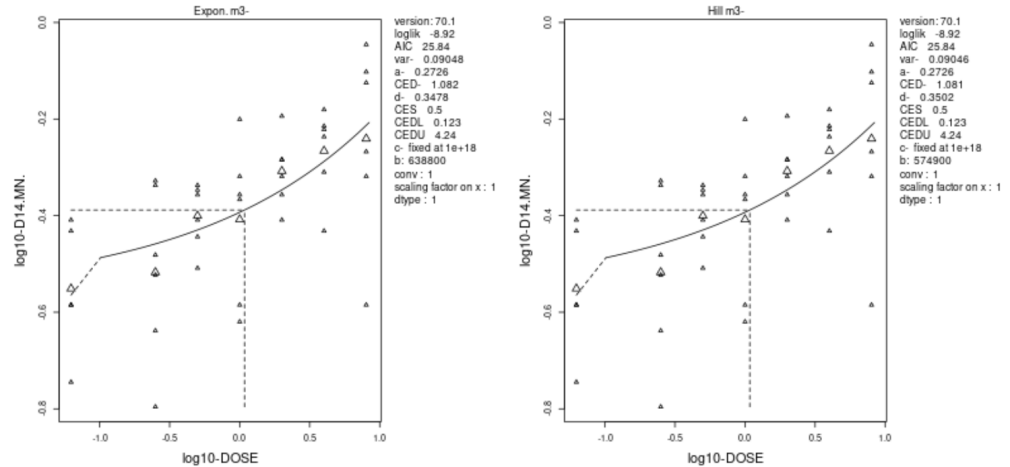


| CES | BMDL (mg/kg.bw) | BMDU (mg/kg.bw) |
| --- | --- | --- |
| 0.5 | 0.13 | 5.08 |

- - 1. **RET at day 14 with CES1SD**


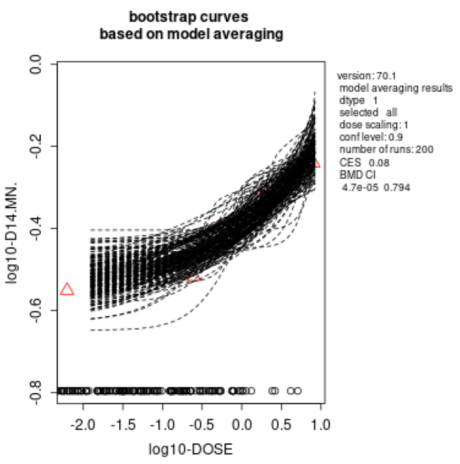

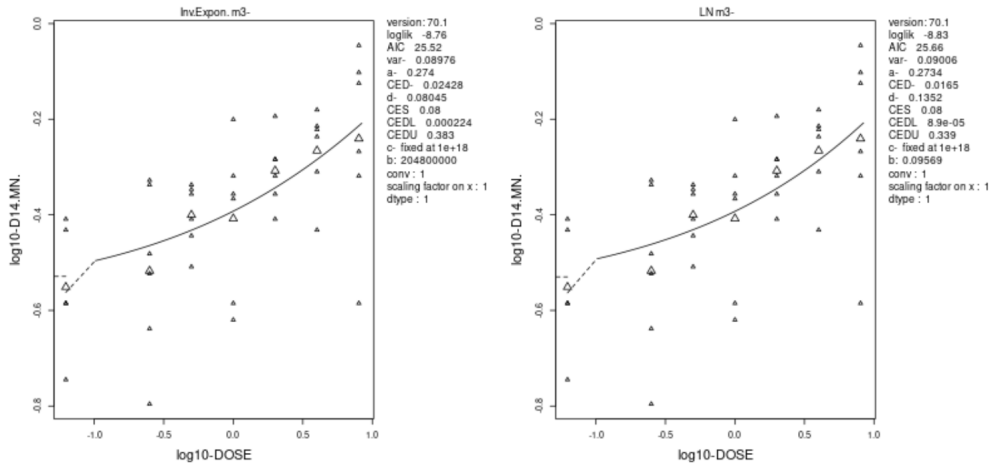


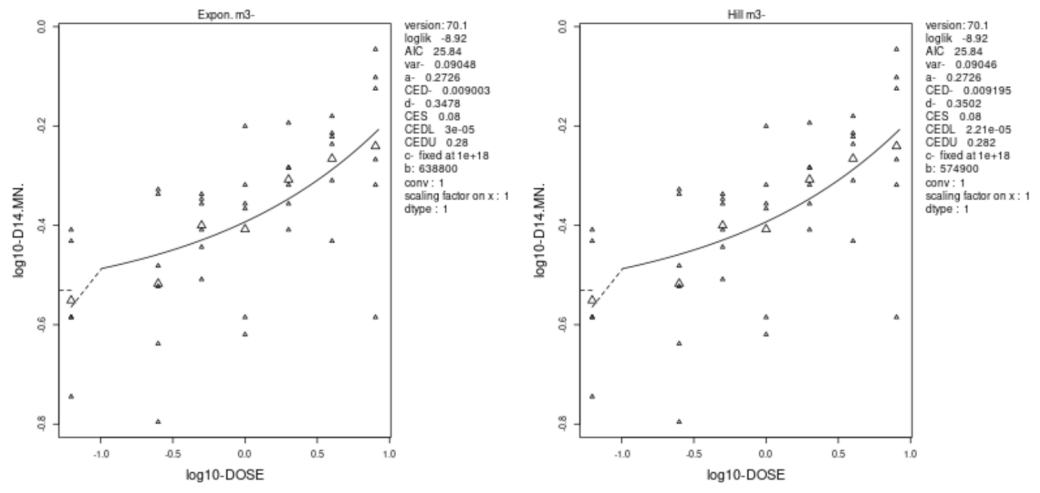


| CES | BMDL (mg/kg.bw) | BMDU (mg/kg.bw) |
| --- | --- | --- |
| 1SD | 4.7e-05 | 0.794 |

- - 1. **RET at day 28 with CES0.05**


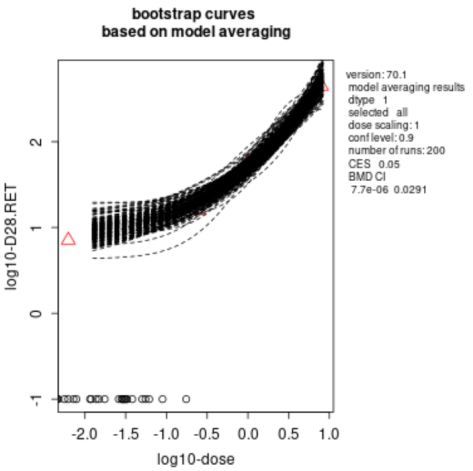

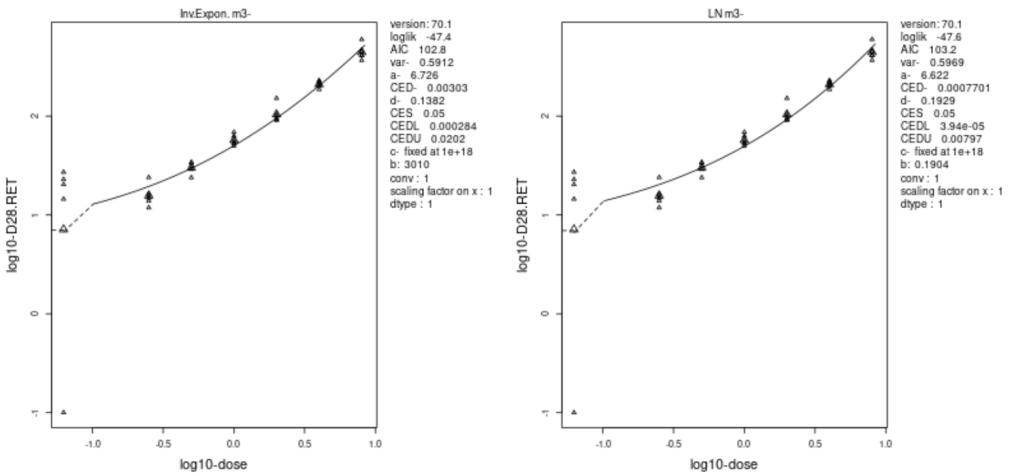


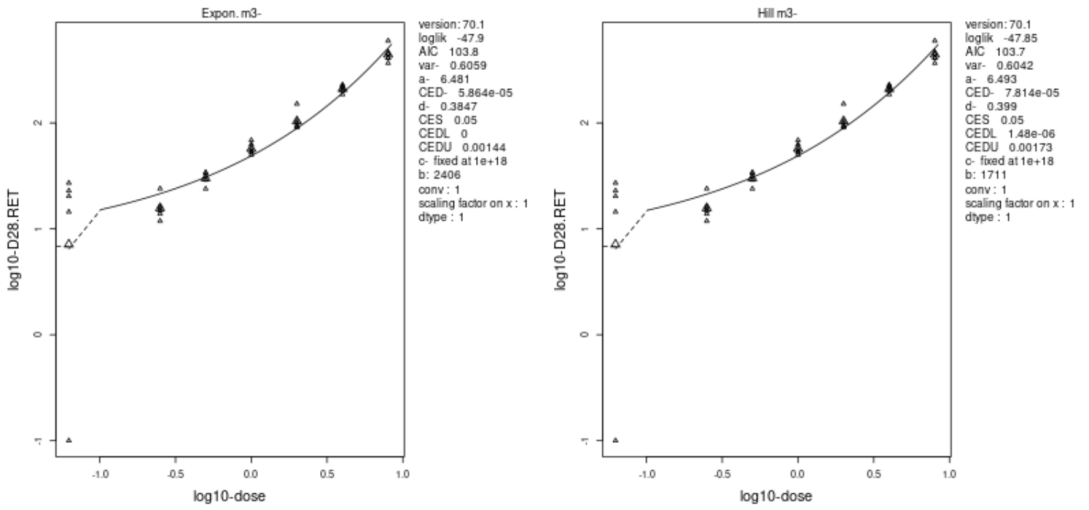


| CES | BMDL (mg/kg.bw) | BMDU (mg/kg.bw) |
| --- | --- | --- |
| 0.05 | 7.7e-06 | 0.0291 |

- - 1. **RET at day 28 with CES0.1**


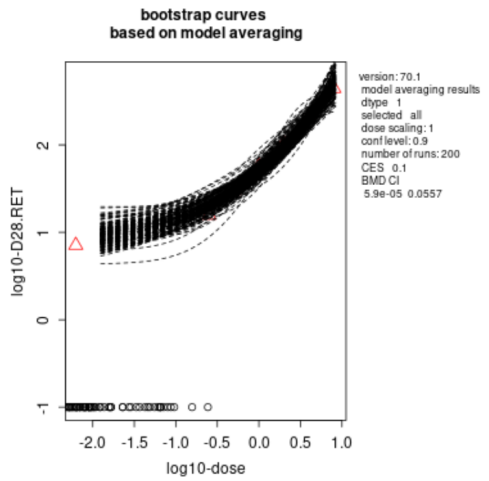

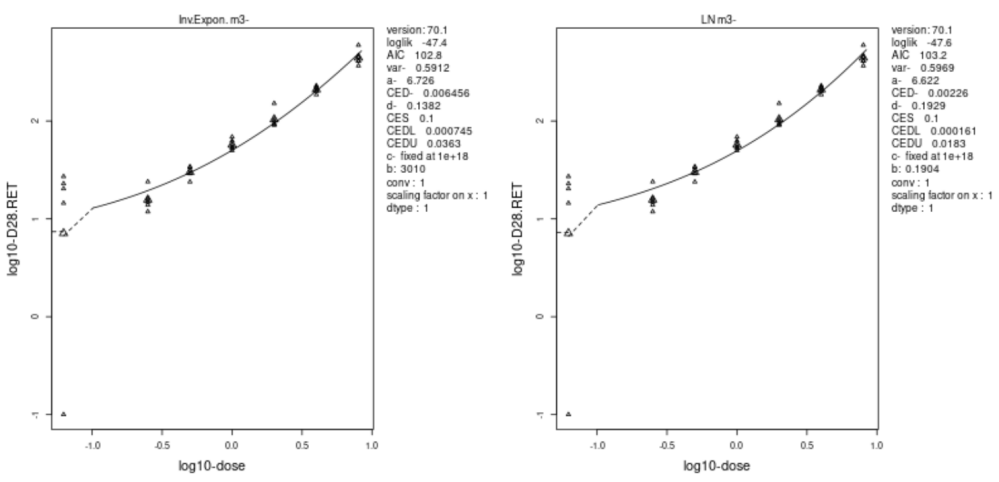


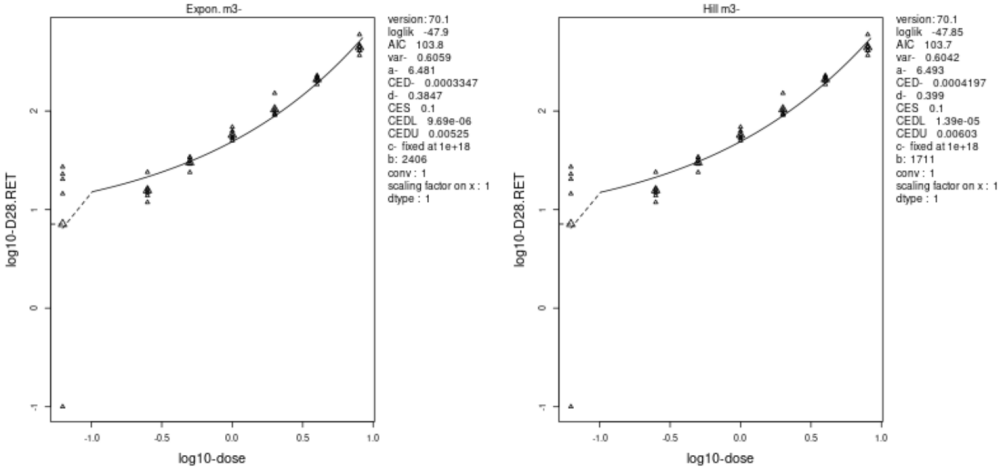


| CES | BMDL (mg/kg.bw) | BMDU (mg/kg.bw) |
| --- | --- | --- |
| 0.1 | 5.9e-05 | 0.0557 |

- - 1. **RET at day 28 with CES0.5**


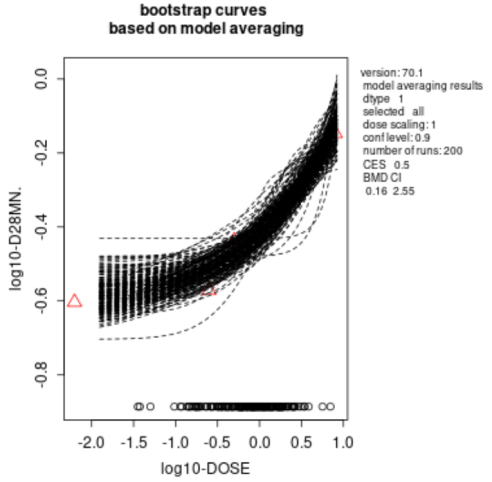

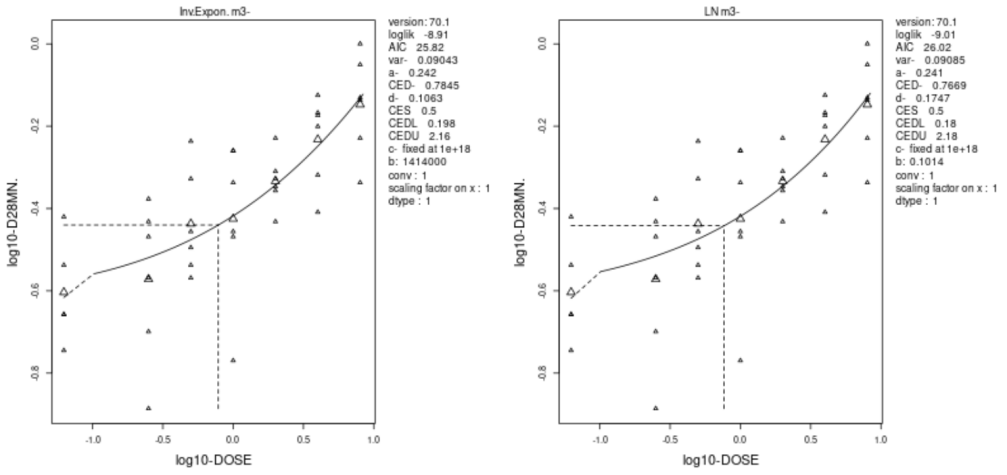


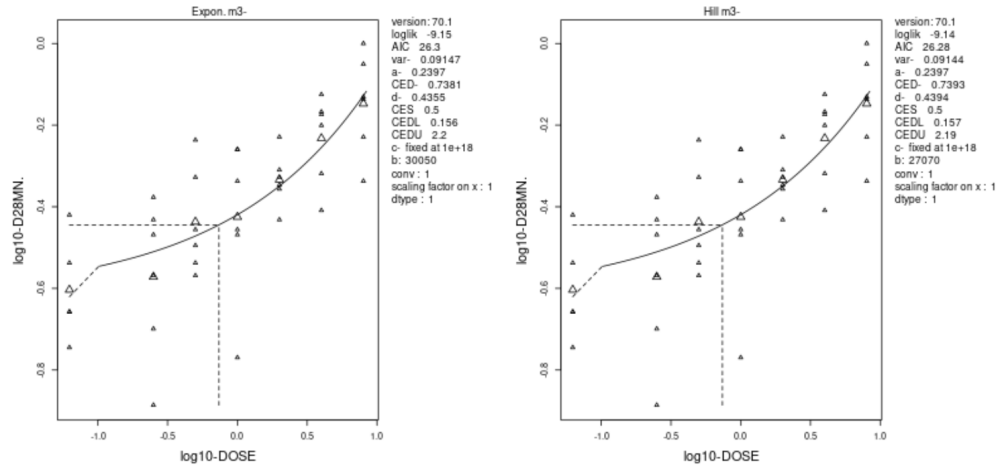


| CES | BMDL (mg/kg.bw) | BMDU (mg/kg.bw) |
| --- | --- | --- |
| 0.5 | 0.16 | 2.55 |

- - 1. **RET at day 28 with CES1SD**


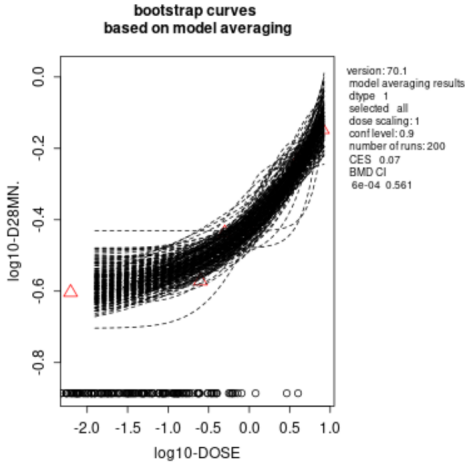

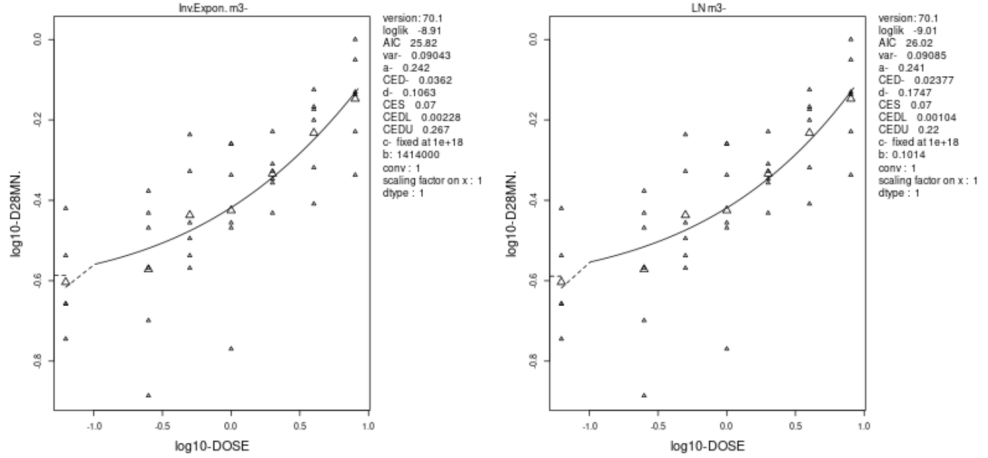


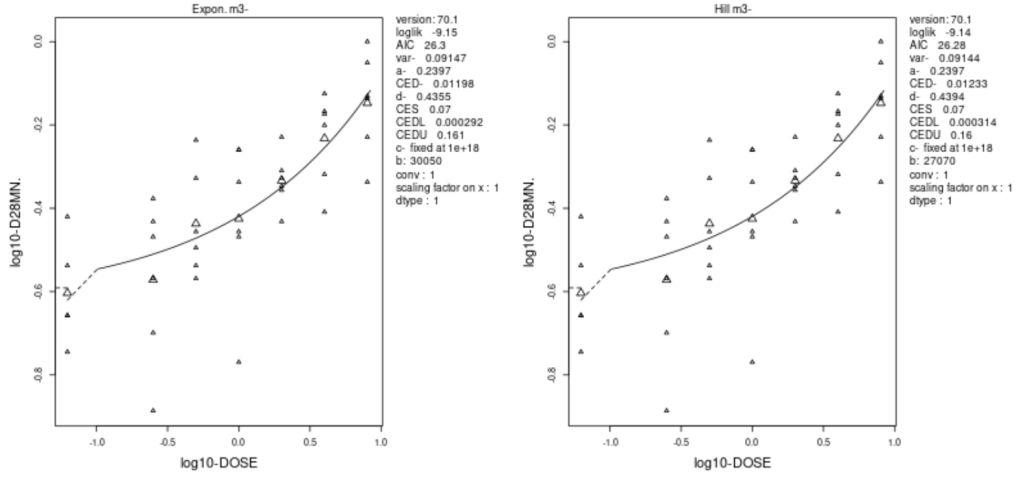


| CES | BMDL (mg/kg.bw) | BMDU (mg/kg.bw) |
| --- | --- | --- |
| 1SD | 6e-04 | 0.561 |

- - 1. **RET at day 29 with CES0.05**


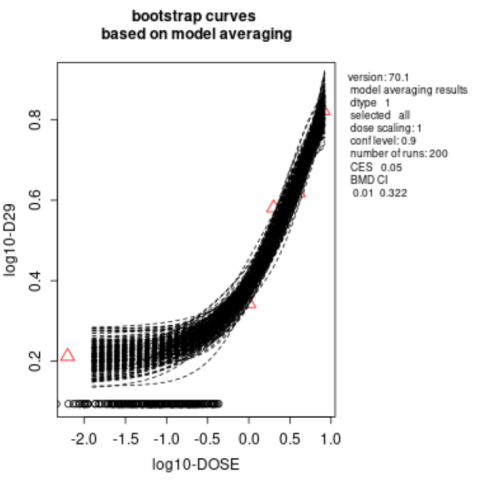

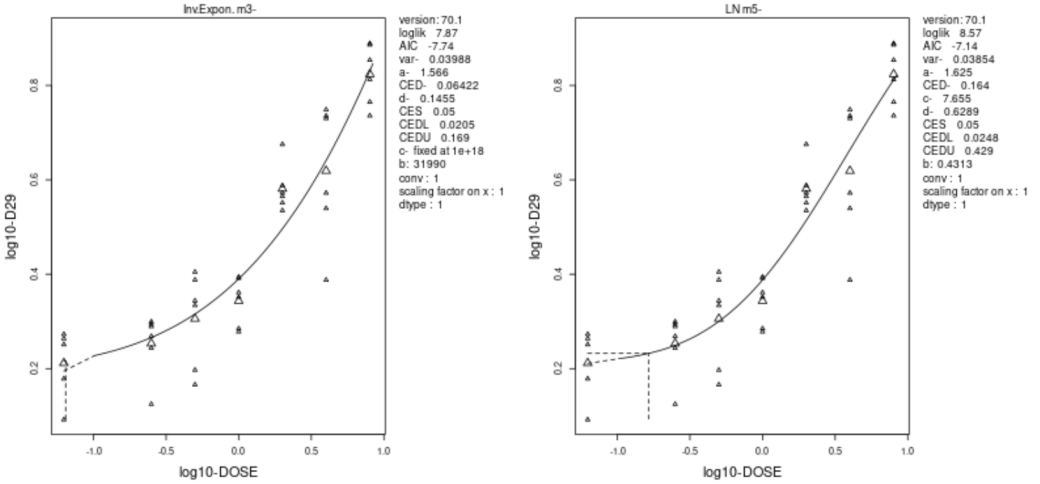


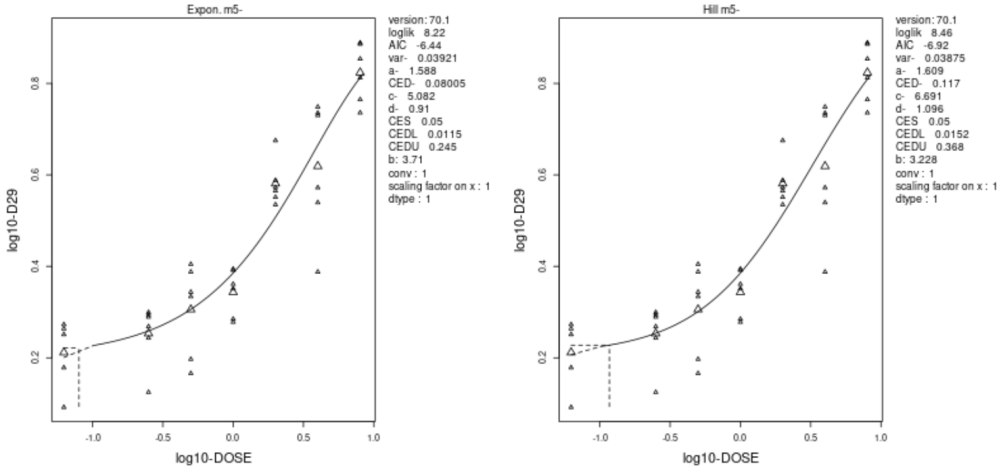


| CES | BMDL (mg/kg.bw) | BMDU (mg/kg.bw) |
| --- | --- | --- |
| 0.05 | 0.01 | 0.322 |

- - 1. **RET at day 29 with CES0.1**


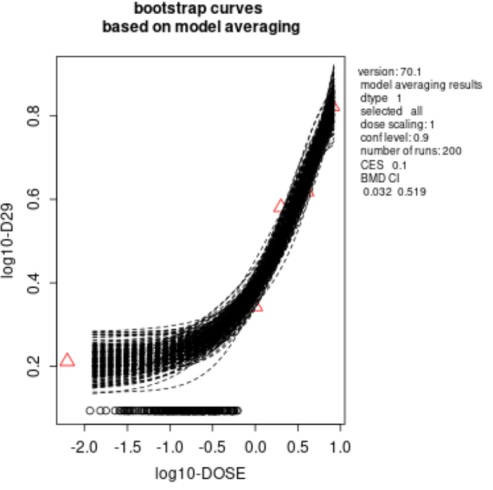

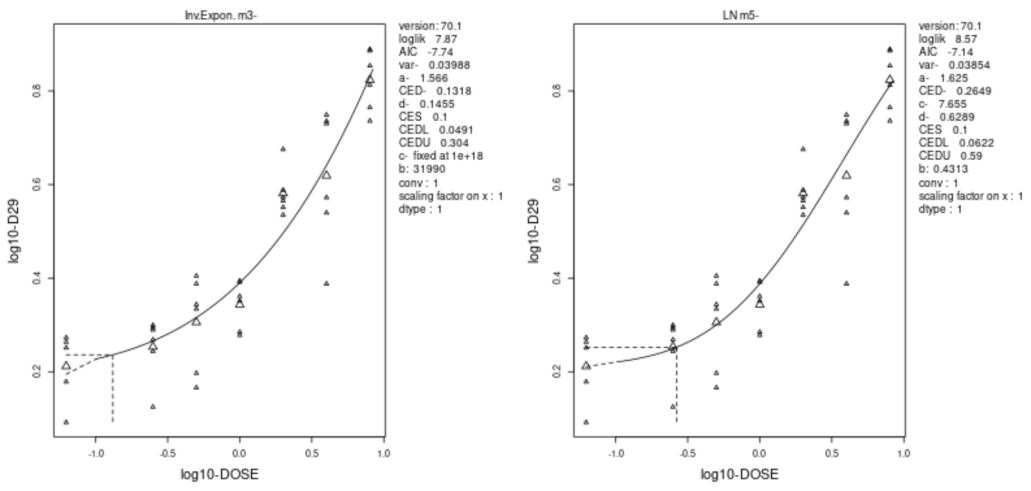


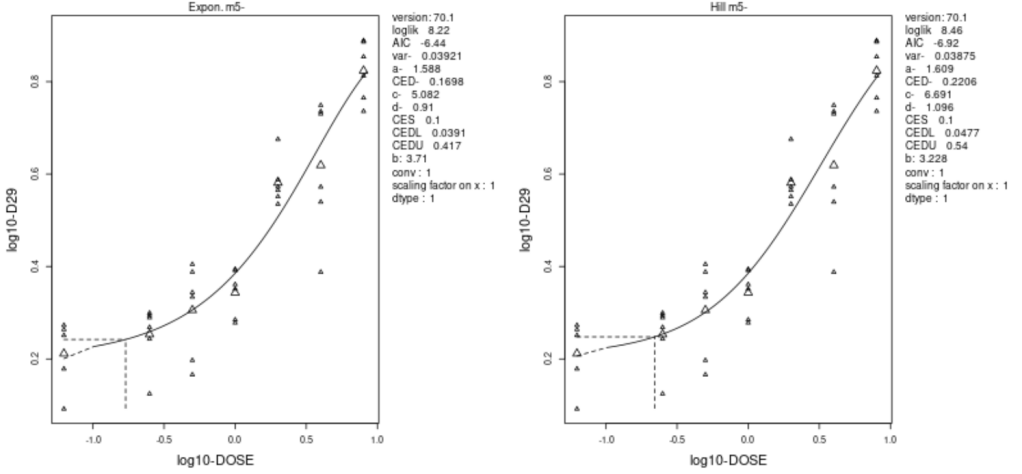


| CES | BMDL (mg/kg.bw) | BMDU (mg/kg.bw) |
| --- | --- | --- |
| 0.1 | 0.032 | 0.519 |

- - 1. **RET at day 29 with CES0.5**


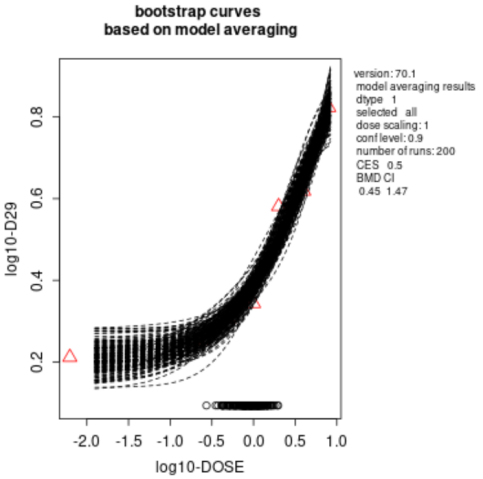

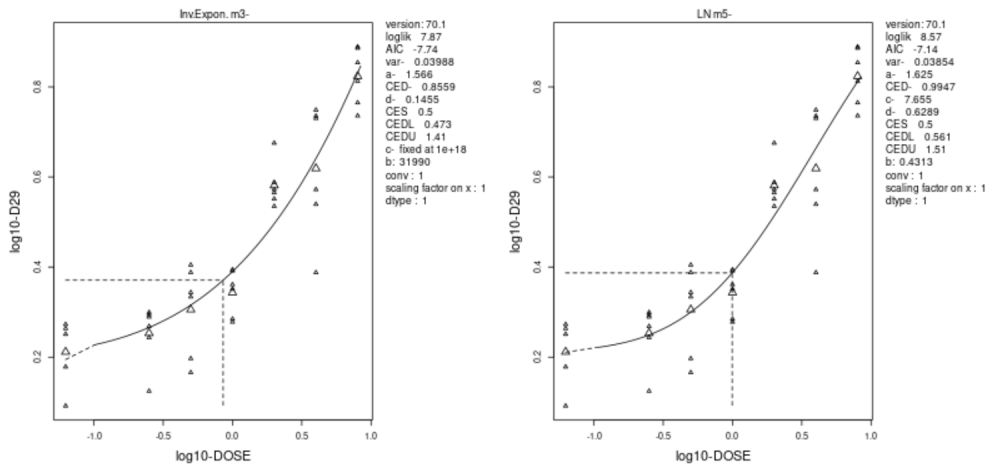


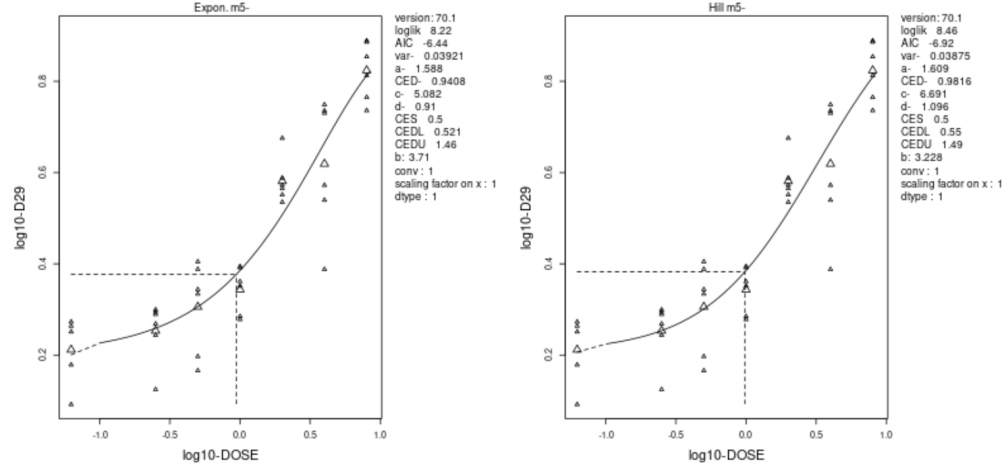


| CES | BMDL (mg/kg.bw) | BMDU (mg/kg.bw) |
| --- | --- | --- |
| 0.5 | 0.45 | 1.47 |

- - 1. **RET at day 29 with CES1SD**


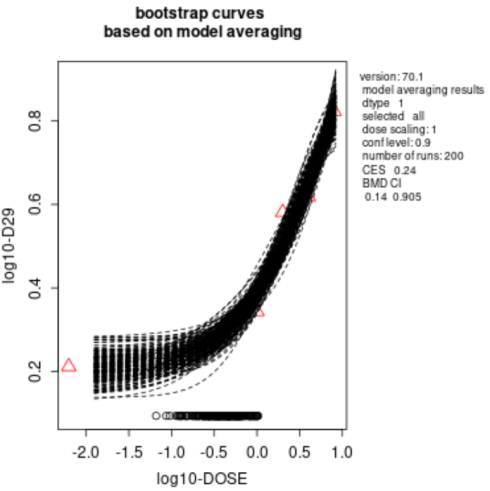

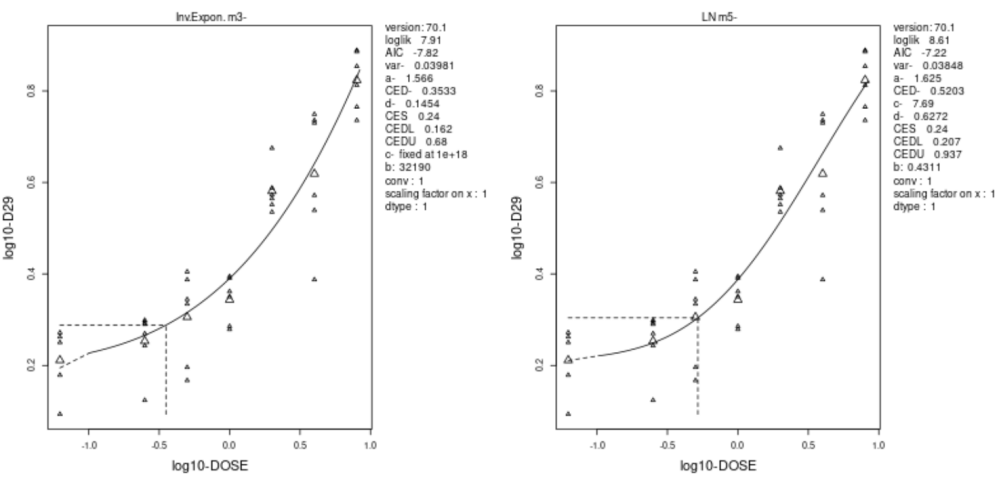


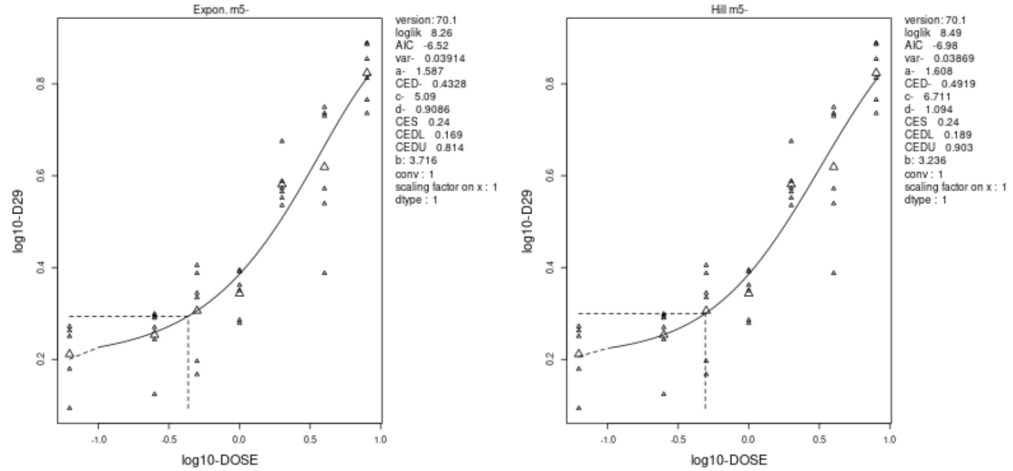


| CES | BMDL (mg/kg.bw) | BMDU (mg/kg.bw) |
| --- | --- | --- |
| 1SD | 0.14 | 0.905 |

- 1. **Comet assay**
     1. **TI at day 4 with CES0.05**


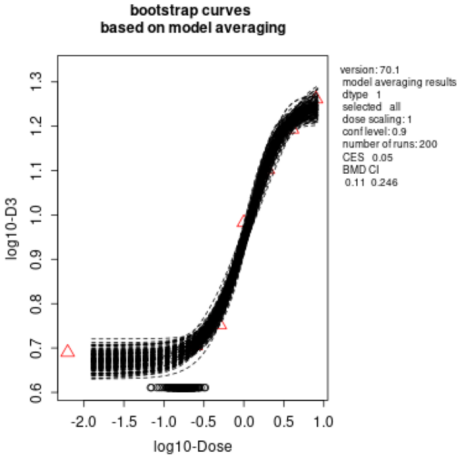

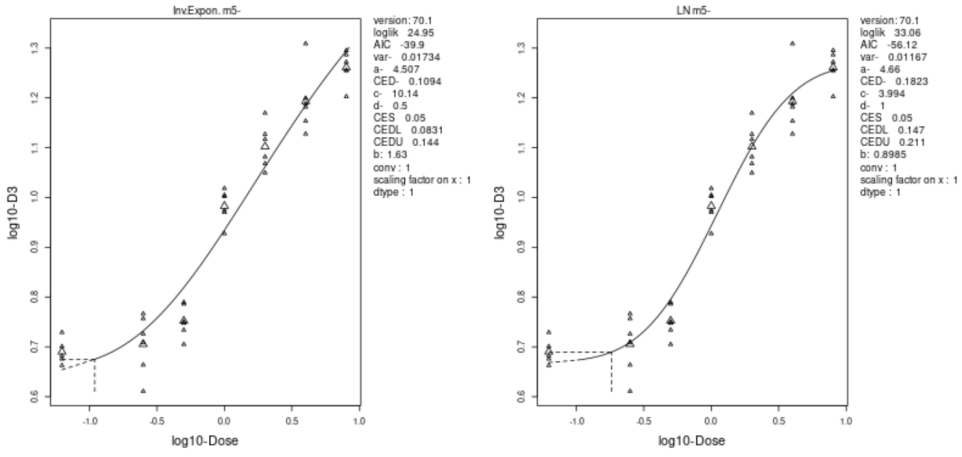


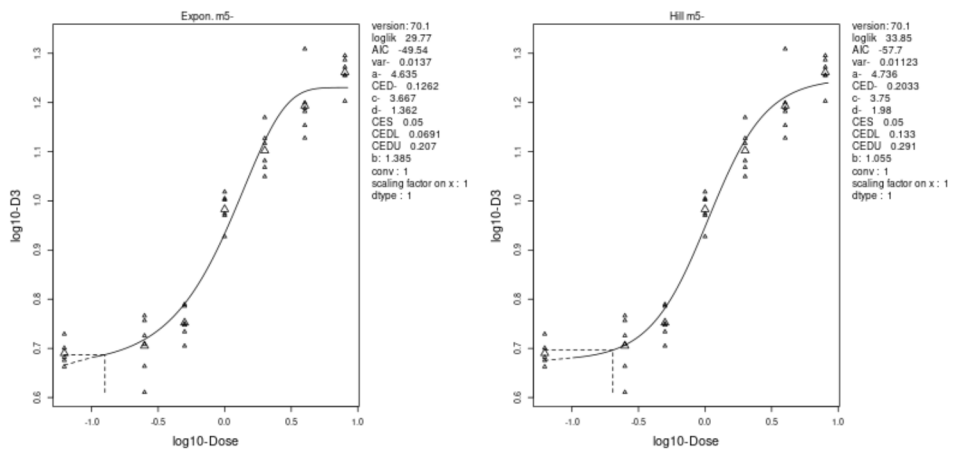


| CES | BMDL (mg/kg.bw) | BMDU (mg/kg.bw) |
| --- | --- | --- |
| 0.05 | 0.11 | 0.246 |

- - 1. **TI at day 4 with CES0.1**


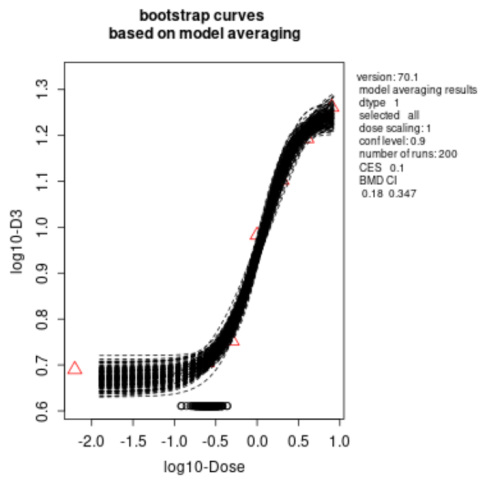


| CES | BMDL (mg/kg.bw) | BMDU (mg/kg.bw) |
| --- | --- | --- |
| 0.1 | 0.18 | 0.347 |

- - 1. **TI at day 4 with CES0.5**

| CES | BMDL (mg/kg.bw) | BMDU (mg/kg.bw) |
| --- | --- | --- |
| 0.5 | 0.55 | 0.813 |

- - 1. **TI at day 4 with CES1SD**

| CES | BMDL (mg/kg.bw) | BMDU (mg/kg.bw) |
| --- | --- | --- |
| 1SD | 0.34 | 0.568 |

- - 1. **TI at day 14 with CES0.05**

| CES | BMDL (mg/kg.bw) | BMDU (mg/kg.bw) |
| --- | --- | --- |
| 0.05 | 0.038 | 0.274 |

- - 1. **TI at day 14 with CES0.1**

| CES | BMDL (mg/kg.bw) | BMDU (mg/kg.bw) |
| --- | --- | --- |
| 0.1 | 0.087 | 0.392 |

- - 1. **TI at day 14 with CES0.5**

| CES | BMDL (mg/kg.bw) | BMDU (mg/kg.bw) |
| --- | --- | --- |
| 0.5 | 0.56 | 1.17 |

- - 1. **TI at day 14 with CES1SD**

| CES | BMDL (mg/kg.bw) | BMDU (mg/kg.bw) |
| --- | --- | --- |
| 1SD | 0.85 | 1.66 |

- - 1. **TI at day 28 with CES0.05**

| CES | BMDL (mg/kg.bw) | BMDU (mg/kg.bw) |
| --- | --- | --- |
| 0.05 | 0.004 | 0.159 |

- - 1. **TI at day 28 with CES0.1**

| CES | BMDL (mg/kg.bw) | BMDU (mg/kg.bw) |
| --- | --- | --- |
| 0.1 | 0.018 | 0.259 |

- - 1. **TI at day 28 with CES0.5**

| CES | BMDL (mg/kg.bw) | BMDU (mg/kg.bw) |
| --- | --- | --- |
| 0.5 | 0.34 | 0.871 |

- - 1. **TI at day 28 with CES1SD**

| CES | BMDL (mg/kg.bw) | BMDU (mg/kg.bw) |
| --- | --- | --- |
| 1SD | 0.32 | 0.842 |

- - 1. **TI at day 29 with CES0.05**

| CES | BMDL (mg/kg.bw) | BMDU (mg/kg.bw) |
| --- | --- | --- |
| 0.05 | 0.17 | 0.323 |

- - 1. **TI at day 29 with CES0.1**

| CES | BMDL (mg/kg.bw) | BMDU (mg/kg.bw) |
| --- | --- | --- |
| 0.1 | 0.25 | 0.415 |

- - 1. **TI at day 29 with CES0.5**

| CES | BMDL (mg/kg.bw) | BMDU (mg/kg.bw) |
| --- | --- | --- |
| 0.5 | 0.62 | 0.806 |

- - 1. **TI at day 29 with CES1SD**

| CES | BMDL (mg/kg.bw) | BMDU (mg/kg.bw) |
| --- | --- | --- |
| 1SD | 0.52 | 0.699 |

1. **The PoDs of genotoxic endpoints of EMS**
   1. ***Pig-a* gene mutation assay**
      1. **RBC at day 14 with CES0.05**

| CES | BMDL (mg/kg.bw) | BMDU (mg/kg.bw) |
| --- | --- | --- |
| 0.05 | 6.6e-06 | 39.4 |

- - 1. **RBC at day 14 with CES0.1**

| CES | BMDL (mg/kg.bw) | BMDU (mg/kg.bw) |
| --- | --- | --- |
| 0.1 | 7.5e-05 | 50.4 |

- - 1. **RBC at day 14 with CES0.5**

| CES | BMDL (mg/kg.bw) | BMDU (mg/kg.bw) |
| --- | --- | --- |
| 0.5 | 0.081 | 97.6 |

- - 1. **RBC at day 14 with CES1SD**

| CES | BMDL (mg/kg.bw) | BMDU (mg/kg.bw) |
| --- | --- | --- |
| 1SD | 2.1 | 131 |

- - 1. **RET at day 14 with CES0.05**

| CES | BMDL (mg/kg.bw) | BMDU (mg/kg.bw) |
| --- | --- | --- |
| 0.05 | 0.045 | 40.2 |

- - 1. **RET at day 14 with CES0.1**

| CES | BMDL (mg/kg.bw) | BMDU (mg/kg.bw) |
| --- | --- | --- |
| 0.1 | 0.15 | 52.1 |

- - 1. **RET at day 14 with CES0.5**

| CES | BMDL (mg/kg.bw) | BMDU (mg/kg.bw) |
| --- | --- | --- |
| 0.5 | 1.7 | 87.2 |

- - 1. **RET at day 14 with CES1SD**

| CES | BMDL (mg/kg.bw) | BMDU (mg/kg.bw) |
| --- | --- | --- |
| 1SD | 53 | 248 |

- - 1. **RBC at day 28 with CES0.05**

| CES | BMDL (mg/kg.bw) | BMDU (mg/kg.bw) |
| --- | --- | --- |
| 0.05 | 1.1 | 9.98 |

- - 1. **RBC at day 28 with CES0.1**

| CES | BMDL (mg/kg.bw) | BMDU (mg/kg.bw) |
| --- | --- | --- |
| 0.1 | 2.3 | 14.3 |

- - 1. **RBC at day 28 with CES0.5**

| CES | BMDL (mg/kg.bw) | BMDU (mg/kg.bw) |
| --- | --- | --- |
| 0.5 | 12 | 32.9 |

- - 1. **RBC at day 28 with CES1SD**

| CES | BMDL (mg/kg.bw) | BMDU (mg/kg.bw) |
| --- | --- | --- |
| 1SD | 14 | 37.8 |

- - 1. **RET at day 28 with CES0.05**

| CES | BMDL (mg/kg.bw) | BMDU (mg/kg.bw) |
| --- | --- | --- |
| 0.05 | 0.083 | 35.2 |

- - 1. **RET at day 28 with CES0.1**

| CES | BMDL (mg/kg.bw) | BMDU (mg/kg.bw) |
| --- | --- | --- |
| 0.1 | 0.3 | 44.7 |

- - 1. **RET at day 28 with CES0.5**

| CES | BMDL (mg/kg.bw) | BMDU (mg/kg.bw) |
| --- | --- | --- |
| 0.5 | 4.7 | 80.1 |

- - 1. **RET at day 28 with CES1SD**

| CES | BMDL (mg/kg.bw) | BMDU (mg/kg.bw) |
| --- | --- | --- |
| 1SD | 110 | 268 |

- 1. **Micronucleus assay**
     1. **RET at day 4 with CES0.05**

| CES | BMDL (mg/kg.bw) | BMDU (mg/kg.bw) |
| --- | --- | --- |
| 0.05 | 3.8 | 78.6 |

- - 1. **RET at day 4 with CES0.1**

| CES | BMDL (mg/kg.bw) | BMDU (mg/kg.bw) |
| --- | --- | --- |
| 0.1 | 12 | 96.2 |

- - 1. **RET at day 4 with CES0.5**

| CES | BMDL (mg/kg.bw) | BMDU (mg/kg.bw) |
| --- | --- | --- |
| 0.5 | 52 | 152 |

- - 1. **RET at day 4 with CES1SD**

| CES | BMDL (mg/kg.bw) | BMDU (mg/kg.bw) |
| --- | --- | --- |
| 1SD | 25 | 119 |

- - 1. **RET at day 14 with CES0.05**

| CES | BMDL (mg/kg.bw) | BMDU (mg/kg.bw) |
| --- | --- | --- |
| 0.05 | 19 | 63.4 |

- - 1. **RET at day 14 with CES0.1**

| CES | BMDL (mg/kg.bw) | BMDU (mg/kg.bw) |
| --- | --- | --- |
| 0.1 | 27 | 77.4 |

- - 1. **RET at day 14 with CES0.5**

| CES | BMDL (mg/kg.bw) | BMDU (mg/kg.bw) |
| --- | --- | --- |
| 0.5 | 63 | 118 |

- - 1. **RET at day 14 with CES1SD**

| CES | BMDL (mg/kg.bw) | BMDU (mg/kg.bw) |
| --- | --- | --- |
| 1SD | 43 | 98.1 |

- - 1. **RET at day 28 with CES0.05**

| CES | BMDL (mg/kg.bw) | BMDU (mg/kg.bw) |
| --- | --- | --- |
| 0.05 | 1.1 | 32.1 |

- - 1. **RET at day 28 with CES0.1**

| CES | BMDL (mg/kg.bw) | BMDU (mg/kg.bw) |
| --- | --- | --- |
| 0.1 | 2.6 | 40.3 |

- - 1. **RET at day 28 with CES0.5**

| CES | BMDL (mg/kg.bw) | BMDU (mg/kg.bw) |
| --- | --- | --- |
| 0.5 | 16 | 78.9 |

- - 1. **RET at day 28 with CES1SD**

| CES | BMDL (mg/kg.bw) | BMDU (mg/kg.bw) |
| --- | --- | --- |
| 1SD | 7.9 | 59.3 |

- - 1. **RET at day 29 with CES0.05**

| CES | BMDL (mg/kg.bw) | BMDU (mg/kg.bw) |
| --- | --- | --- |
| 0.05 | 0.99 | 18.5 |

- - 1. **RET at day 29 with CES0.1**

| CES | BMDL (mg/kg.bw) | BMDU (mg/kg.bw) |
| --- | --- | --- |
| 0.1 | 2.6 | 22.8 |

- - 1. **RET at day 29 with CES0.5**

| CES | BMDL (mg/kg.bw) | BMDU (mg/kg.bw) |
| --- | --- | --- |
| 0.5 | 15 | 37.9 |

- - 1. **RET at day 29 with CES1SD**

| CES | BMDL (mg/kg.bw) | BMDU (mg/kg.bw) |
| --- | --- | --- |
| 1SD | 5.8 | 27.4 |

- 1. **Comet assay**
     1. **TI at day 4 with CES0.05**

| CES | BMDL (mg/kg.bw) | BMDU (mg/kg.bw) |
| --- | --- | --- |
| 0.05 | 0.86 | 7.43 |

- - 1. **TI at day 4 with CES0.1**

| CES | BMDL (mg/kg.bw) | BMDU (mg/kg.bw) |
| --- | --- | --- |
| 0.1 | 1.9 | 10.8 |

- - 1. **TI at day 4 with CES0.5**

| CES | BMDL (mg/kg.bw) | BMDU (mg/kg.bw) |
| --- | --- | --- |
| 0.5 | 11 | 26.6 |

- - 1. **TI at day 4 with CES1SD**

| CES | BMDL (mg/kg.bw) | BMDU (mg/kg.bw) |
| --- | --- | --- |
| 1SD | 22 | 38.2 |

- - 1. **TI at day 28 with CES0.05**

| CES | BMDL (mg/kg.bw) | BMDU (mg/kg.bw) |
| --- | --- | --- |
| 0.05 | 1 | 10.8 |

- - 1. **TI at day 28 with CES0.1**

| CES | BMDL (mg/kg.bw) | BMDU (mg/kg.bw) |
| --- | --- | --- |
| 0.1 | 2.1 | 14.8 |

- - 1. **TI at day 28 with CES0.5**

| CES | BMDL (mg/kg.bw) | BMDU (mg/kg.bw) |
| --- | --- | --- |
| 0.5 | 13 | 31.2 |

- - 1. **TI at day 28 with CES1SD**

| CES | BMDL (mg/kg.bw) | BMDU (mg/kg.bw) |
| --- | --- | --- |
| 1SD | 23 | 44.2 |

- - 1. **TI at day 29 with CES0.05**

| CES | BMDL (mg/kg.bw) | BMDU (mg/kg.bw) |
| --- | --- | --- |
| 0.05 | 6.2 | 13.3 |

- - 1. **TI at day 29 with CES0.1**

| CES | BMDL (mg/kg.bw) | BMDU (mg/kg.bw) |
| --- | --- | --- |
| 0.1 | 8.9 | 17.2 |

- - 1. **TI at day 29 with CES0.5**

| CES | BMDL (mg/kg.bw) | BMDU (mg/kg.bw) |
| --- | --- | --- |
| 0.5 | 22 | 31.1 |

- - 1. **TI at day 29 with CES1SD**

| CES | BMDL (mg/kg.bw) | BMDU (mg/kg.bw) |
| --- | --- | --- |
| 1SD | 28 | 38 |
